# *In-Vivo* Expression Profiling of *Pseudomonas aeruginosa* Infections Reveals Niche-Specific and Strain-Independent Transcriptional Programs

**DOI:** 10.1371/journal.pone.0024235

**Published:** 2011-09-12

**Authors:** Piotr Bielecki, Jacek Puchałka, Melissa L. Wos-Oxley, Holger Loessner, Justyna Glik, Marek Kawecki, Mariusz Nowak, Burkhard Tümmler, Siegfried Weiss, Vítor A. P. Martins dos Santos

**Affiliations:** 1 Systems and Synthetic Biology, Helmholtz Centre for Infection Research (HZI), Braunschweig, Germany; 2 Environmental Microbiology, Helmholtz Centre for Infection Research (HZI), Braunschweig, Germany; 3 Microbial Interactions and Processes, Helmholtz Centre for Infection Research (HZI), Braunschweig, Germany; 4 Molecular Immunology Research Group, Helmholtz Centre for Infection Research (HZI), Braunschweig, Germany; 5 Klinische Forschergruppe, Medizinische Hochschule Hannover, Hannover, Germany; 6 Center for Burn Treatment, Siemianowice Śląskie, Poland; 7 Department of Health Sciences, Technical-Humanistic Academy, Bielsko-Biała, Poland; 8 Laboratory of Systems and Synthetic Biology, Agrotechnology and Food Sciences, Wageningen University, Wageningen, Netherlands; 9 Department of Pathophysiology of Bacterial Biofilms, Centre for Experimental and Clinical Infection Research, Twincore, Hanover, Germany; University of Edinburgh, United Kingdom

## Abstract

*Pseudomonas aeruginosa* is a threatening, opportunistic pathogen causing disease in immunocompromised individuals. The hallmark of *P. aeruginosa* virulence is its multi-factorial and combinatorial nature. It renders such bacteria infectious for many organisms and it is often resistant to antibiotics. To gain insights into the physiology of *P. aeruginosa* during infection, we assessed the transcriptional programs of three different *P. aeruginosa* strains directly after isolation from burn wounds of humans. We compared the programs to those of the same strains using two infection models: a plant model, which consisted of the infection of the midrib of lettuce leaves, and a murine tumor model, which was obtained by infection of mice with an induced tumor in the abdomen. All control conditions of *P. aeruginosa* cells growing in suspension and as a biofilm were added to the analysis. We found that these different *P. aeruginosa* strains express a pool of distinct genetic traits that are activated under particular infection conditions regardless of their genetic variability. The knowledge herein generated will advance our understanding of *P. aeruginosa* virulence and provide valuable cues for the definition of prospective targets to develop novel intervention strategies.

## Introduction

Many opportunistic microbial pathogens are capable of adapting to multiple niches. However, it is not yet clear whether they adapt to different niches through a similar set of mechanisms and virulence factors or whether they express a unique set of factors for each particular environment. *Pseudomonas aeruginosa* is a paradigm of such an opportunistic pathogen [1]. It is found ubiquitously in the environment and can be isolated from water and soil but it is also a prevalent member in infections of immunocompromised patients such as burn wound victims or in the lungs of patients suffering from cystic fibrosis [2]. In addition, it is capable of infecting a range of non-vertebrate eukaryotic organisms like *Caenorhabditis elegans* [3] and *Drosophila melanogaster* [4], as well as plants such as *Arabidopsis thaliana* and lettuce [5]. The genome of several strains has been sequenced (www.pseudomonas.com) and the comparison thereof has revealed that many virulence factors are encoded in accessory genetic elements - genomic islands – that have been acquired as a strategy for survival under particular conditions [6]. In general, virulence of *P. aeruginosa* is assumed to be combinatorial, i.e., divergent infectious potential may be based on the presence or absence of particular pathogenicity islands [7]. This suggests that genome plasticity is important for its survival in different niches [8]. However, this does not suffice to explain the remarkable capability of this organism to infect a range of hosts under many conditions. Furthermore, we lack knowledge pertaining to the genetic programs resulting from different infection settings and the degree to which strain-specificity determines such genetic programs.

Therefore, we tested this paradigm at the level of gene expression. We compared the *in vivo* transcriptional profiles of three different *P. aeruginosa* strains from burn wound victims, with those determined in two alternative infection systems, namely a plant (lettuce) [9] and a solid murine tumor (Komor et al, submitted), and two *in vitro* culture conditions (planktonic and sessile) using microarrays designed on the genome of *P. aeruginosa* PAO1. This allowed comparing the expression profiles of the core genome that is conserved among all *P. aeruginosa.* Comparison of transcriptional profiles of the same clinical strains over this broad range of conditions revealed that the investigated *P. aeruginosa* strains express a pool of distinct genetic traits from their core genome that are activated under particular infection settings regardless of their genetic variability. Furthermore, the expression profiling for each of the individual conditions provided valuable insights into the underlying transcriptional programs. Importantly, the specific analysis of *in vivo* transcription of *P. aeruginosa* infecting burn wound patients pinpointed a large number of genes coding for hypothetical proteins that are logical candidates for subsequent functional characterization.

## Results and Discussion

### *Pseudomonas aeruginosa* isolates from burn wound patients

We recovered and characterized *P. aeruginosa* strains from the exudates of three burn wound patients. The isolates were tested for antibiotic resistance (Table S1) and for clonal variability using binary arrays [10]. The isolates of the three exudates for which sufficient material was obtained for subsequent determination of *in vivo* gene expression were named *P. aeruginosa* PBCLOp10, PBCLOp11 and PBCLOp17. All three isolates were clonally distinct. The three strains belong to clonal complexes of variable abundance in the global population. The genotype of isolate CLOPBp10 is yet unique in a collection of currently 1,600 genotyped *P. aeruginosa* strains of independent origin, whereas the isolate CLOPBp17 belongs to a clone of intermediate occurrence (0.6% of the population). Isolate CLOPBp11 is a representative of the frequent clone D that worldwide is highly prevalent among keratitis isolates [11]. The antibiotic resistance profiles also differ between the clinical isolates and the control laboratory strains PAO1 and PA14 used as controls for susceptibility test. Strain PBCLOp10 had higher levels of resistance to piperacilin, as well as to antibiotics from the group of meropenems and aminoglicosydes, strain PBCLOp11 show resistance to ciprofloxacin. Strain PBCLOp17 emerged as being multi-resistant, with resistance to all antibiotics in our analysis except carbapenem – meropenem (full results Table S1). The antibiotics resistance pattern suggests that these strains were already specialized as nosocomial pathogens. Taking into account both the difference in the antibiotic resistance phenotype and the difference in the multimarker genotype, we conclude that the isolates are genetically distinct from each other, even though they were isolated from the same habitat and the same geographical site (albeit different patients at different times).

### Genomic plasticity or niche specificity?

**Figure 1:**
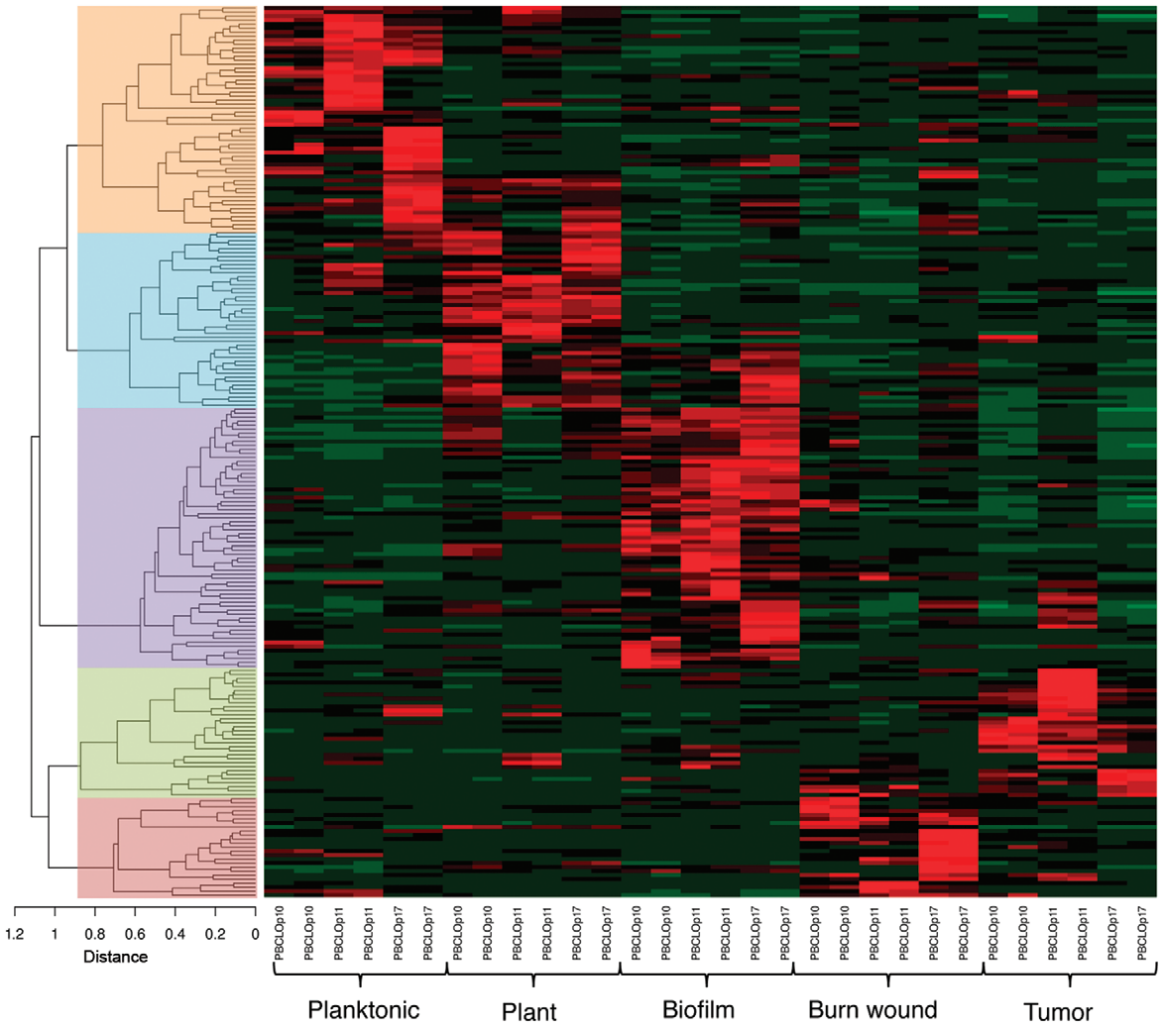
Hierarchical clustering of expressed genes, where gene expression is presented as a heat map. Five broad groups of genes at a cutoff of 0.9 (indicated by the colored boxes) on the hierarchical cluster dendrogram represent signature genes in each of the five conditions. These genes are listed in Tables S2, S3, S4, S5, S6. Distance was measured by the 1-correlation algorithm.

We compared the transcriptional profiles *in vivo* of the three different *P. aeruginosa* isolates from burn wound patients, with those determined in two alternative infection systems, namely a plant (lettuce) and a solid murine tumor, and two *in vitro* culture conditions (planktonic and sessile). The transcriptional profiles for each of the three independent strains under each of the five conditions were performed in duplicate. By treating the three *P. aeruginosa* strains as one data set for the purpose of the analysis (See Materials and Methods), a picture of general *P. aeruginosa* features emerged rather than those specific for the single strain. Genes that showed a significant expression difference (percentage of false-positives pfp<0.05) in at least one pairwise comparison (1734 genes) between each of the five conditions were grouped using hierarchical clustering with a 1-correlation algorithm as the distance metric. As a result, 213 groups of genes were resolved (at a cutoff of 0.15) (Fig. 1). These groups of genes subsequently assembled into five broader groups (at a cutoff at 0.9), where each of the five groups represented genes that were unique to each of the five studied conditions (the entire list of genes is presented in the tables S2, S3, S4, S5, S6 of the SI; pairwise comparisons between the various conditions are shown in table S9). The level of expression is depicted in the hierarchical cluster dendrogram as a heat map (Fig. 1) to facilitate the visualization and comparison of the degree of gene expression under the various conditions. These data clearly indicate that these three independent *P. aeruginosa* isolates regulate their genes in a similar manner when subjected to the same conditions. Altogether, these analyses indicate that while the genomic contents and plasticity of nosocomial *P. aeruginosa* strains are important in conferring specific capabilities upon infection, the niche where they thrive largely determines the prevalent transcriptional programs and, most likely, the resulting phenotypes.

### Assessing models of infection

**Figure 2:**
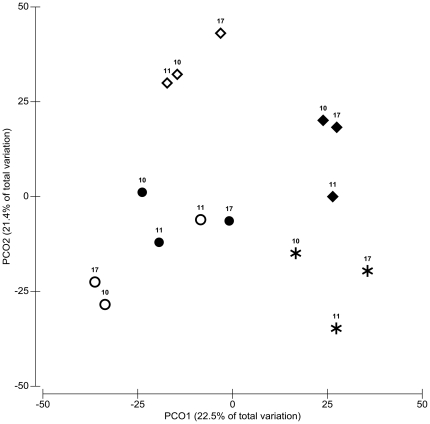
Global expression patterns of *P. aeruginosa* strains in the tumor infection (open circles) and those of the burn wound infection (closed circles) co-localized at the centre left of the PCO plot, while the global expression of the strains from the planktonic growth (asterisks) ordinated to the lower right of the plot, biofilm (open diamonds) ordinated upper of the plot and lettuce infection model (closed diamonds) ordinated at the right of the plot. This result was confirmed by PERMANOVA, where all conditions were significantly different from each other (*p*<0.05) except the burn wound and tumor infection (*p* = 0.1108).

Permutational multivariate analysis of variance (PERMANOVA) showed that the condition, under which each independent strain was subjected, significantly influenced its transcriptional profile (Pseudo *F* = 3.863, *p* = 0.0001). The five studied conditions were all significantly different to each other, with the exception of the burn wound and mouse tumor conditions, which were not significantly different (PERMANOVA Pseudo *F* = 1.5182, *p* = 0.1108) (Table 1). This is also clearly seen in the principal coordinate analysis (PCO) plot (Fig. 2), which shows that the transcriptional patterns of both the burn wound and murine tumor infections partially overlap. This underscores the view that the murine tumors represent a more appropriate model for a human infection with *P. aeruginosa*. By contrast, the *in vivo* infection of lettuce had a largely different expression pattern as compared to that of the burn wound or the murine tumor (*p* = 0.0316 and *p* = 0.0275), respectively. This multi-condition analysis shows that non-mammalian infection models still only partly reflect the real human infection condition and highlights the importance of *in vivo* transcription profiling of a pathogen directly from the infection site for the better understanding of the infection process and the underlying mechanisms.

**Table 1:** Pairwise comparison of the global expression patterns of *P. aeruginosa* strains between each condition.

	PERMANOVA*	
Pairwise comparison between each condition	test-statistic (*t*-value)†	*p*-value (MC)‡
Burn - Tumor	1.5182	0.1108
Planktonic - Lettuce	1.8634	0.0490
Planktonic - Burn	1.8058	0.0486
Planktonic - Tumor	1.8553	0.0416
Burn - Biofilm	1.9430	0.0353
Biofilm - Tumor	2.0592	0.0334
Burn - Lettuce	2.0103	0.0316
Tumor - Lettuce	2.2231	0.0275
Biofilm - Lettuce	2.2047	0.0221
Planktonic - Biofilm	2.2104	0.0219

*Permutational multivariate analysis of variance (PERMANOVA) on unrestricted permutation of raw data was conducted on the gene expression data after a resemblance-similarity matrix was generated using Euclidean distance.

† Pairwise *a posteriori* tests among conditions using the *t* statistic.

‡ As the number of unique values under permutations was very low (possible unique permutations = 10), *p*-values were obtained using 9999 Monte Carlo (MC) samples from the asymptotic permutation distribution. Otherwise, the *p*-value could only ever be 0.1 if the total number of possible unique permutations when comparing 3 replicate strains across a pair of conditions was 10. Significance was set at alpha α = 0.05.

### Niche-specific transcriptional signatures

The analysis of the *in vivo* gene expression profiles of the groups of genes mentioned above pinpoints sets of genes and circuits (signatures) specifically involved in the virulence and survival of *P. aeruginosa* in different settings (Tables S2, S3, S4, S5, S6, S9). Below we will focus on the *in vivo* infection settings and address some of the most prominent features for each of these.

#### Burn wound infection

Burn wound infections trigger the overexpression of common virulence factors such as proteases, exopolysaccharides or iron acquisition systems (Table S2). A large number of genes are known to be up-regulated during iron starvation [12], such as pyoverdin, proteins responsible for heme acquisition, as well as *sodM* and *fumC1* encoding superoxide dismutase and fumarate hydratase, respectively. The similarity of the bacterial transcriptome upon burn wound infection with that of *P. aeruginosa* grown under iron starvation *in vitro* confirmed this finding (Table S7). Thus, the expression of genes involved in iron starvation responses appears to be a hallmark for burn wound infection, as *P. aeruginosa* needs to overcome iron limitation to successfully colonize this niche. It is also striking that the whole group of genes PA2134 – PA2190 is expressed among the genes that are hypothesized to be specific for burn wound infections. This region contains genes with possible roles in the accumulation and breakdown of storage materials such as glycogen and trehalose (*glgA, glgB, glgP*) [13], in protection against oxidative stress like the catalase encoding gene *katE,* or in general stress response (PA2190).

#### Plant infection

We found that the up-regulation of sulfate reduction in *P. aeruginosa* (*cysAWT*, *cysND* and *cysI*,) was a prominent feature of the genetic program active upon infection of lettuce leaves (Table S3). Similar findings were reported by Weir and colleagues [14]. The differential regulation of the sulfate reduction system to overcome sulfate limitation seems thus to be characteristic of the plant infection. The up-regulation of the assimilatory nitrate reductase genes (*nasC, nirB, nirD*) and of the transporter encoding gene *nasA*, as well as the putative glutamate synthase (PA0296, PA0298) suggest that nitrogen is also limitedly available for *P. aeruginosa* infecting lettuce leaves.

Another important difference between the two *in vivo* infection settings is a clear shift in metabolism. For instance, the gene *cbrB*, which codes for the response regulator of the two-component system CbrA/CbrB, was consistently up-regulated and expressed only under plant infection conditions. This system controls several catabolic pathways and the utilization of a variety of aromatic compounds as sole carbon source [15]. CbrAB, together with NtrBC, is important to maintain the carbon-to-nitrogen balance and serves as catabolic repression system in *P. aeruginosa* [15], [16]. A cascade of CbrAB and CrcZ, a small RNA that acts on the Crc protein, also controls the expression of XylS (BenR), which is a major regulator of the degradation of aromatic compounds [17]. The observations suggest that *P. aeruginosa* uses plant cell materials (debris) upon infection as carbon and energy source.

#### Murine tumor infection

The murine tumor infection model (Komor et al., submitted) can be distinguished by the up-regulation of genes (Table S4) belonging to the type III secretion system (T3SS). The operons *pscSPO, pcr123R, exsD-pscDEFGHIJK* encode the secretion apparatus, *pcrVH-popBD* the protein translocation system and *exsEB,* a specific regulator. The genes *exoT*, *exoS* and *exoY*, which encode exoenzymes secreted via the T3SS, were also over-expressed. Owing to their role in inhibiting host-cell protein synthesis, hindering phagocytosis and disruption of cytoskeleton, these exoenzymes are important in the arsenal of *P. aeruginosa* to combat the mammalian host defenses [18]. The presence of the T3SS and secreted enzymes thus clearly reflects the virulent state of *P. aeruginosa* residing in the tumor. Another relevant observation was the up-regulation of most of the genes that belong to the cluster responsible for the synthesis of the B-band of O-antigen of the lipopolisacharide: *wzz*, *wbpABDE*,*wzx*, *wzy* and *wbpGHIJ*. The B-band O-antigen unit is a critical virulence factor that has been shown to play a key role in host colonization and evasion of immune defenses [19], [20]. The *cupA1* (PA2128) gene, which belongs to the *cup* operon, was also upregulated. This operon codes for the different components and assembly factors of a putative fimbrial structure required for biofilm formation [21]. The ability to form biofilms is one of the factors for successful chronic infection by *P. aeruginosa* [22]. The expression of the *cupA1* gene has been also shown to be linked to anaerobiosis [23]. The capacity of *P. aeruginosa* to thrive under anoxic conditions is paramount for its success as a pathogen [24]. The overexpression of the genes responsible for nitrite reduction, *nirSMCDE* and regulator *nirQ*, as well of the genes PA3417, PA3415 and PA0836, which code for enzymes involved in pyruvate fermentation indicate that the *P. aeruginosa* strains were indeed subjected to anoxia. The induction of the arginine deaminase pathway, central in the fermentation of arginine under anoxic conditions, and of the *arcDABC* genes, which have been recently shown to be co-regulated with genes responsible for pyruvate fermentation supports this hypothesis [25]. The up-regulation of PA3309 and PA4352, which code for two universal stress proteins linked to anaerobic survival with pyruvate fermentation, underscores this further.

#### Multi-level gene regulation

The genome-wide gene expression of *P. aeruginosa* is controlled through an extensive network of transcriptional regulators, two-component regulatory systems and sigma factors [1]. Our results shed some light onto the complexity of gene regulation in the various infection settings. For example, the quorum sensing (QS) network has become a paradigm in *P. aeruginosa* for the association between cell-density-dependent gene expression and virulence [26], [27]. The QS systems Las and Rhl, however, were found to be expressed most strongly during planktonic growth *in vitro*, but QS-dependent targets of virulence like exotoxin A or protease IV were more strongly up-regulated in the burn wound, which only showed moderate activation of QS systems as compared to planctonic growth. Similarly, the QS elements VqsR [28] and PQS were identified in our analysis to be strongly activated in biofilms *in vitro*, but again the targets of VqsR and PQS were more strongly expressed in the burn wounds *in vivo* than in biofilms *in vitro*. Thus, it is striking to note that various QS systems were more expressed in inanimate habitats than in the presence of an infected host, but that still the known downstream targets of pathogenicity factors are more strongly up-regulated in the infection setting. Moreover, virulence and global metabolism appear to be co-regulated, as illustrated by activation of the CbrA/CbrB two-component system in the *P. aeruginosa* infecting lettuce. This two-component system is involved in maintaining the carbon-nitrogen balances in check. To enable further exploration of the many interactions underlying specific signatures, we present the pairwise comparisons between all the studied conditions in the SI table S9. By enabling to place the large number of differentially expressed genes onto accurate genome-scale scaffolds, the development of genome-scale mathematical models of metabolism and regulation [29], [30] will greatly facilitate the future interpretation of such datasets.

#### Testing specific signature genes

**Figure 3:**
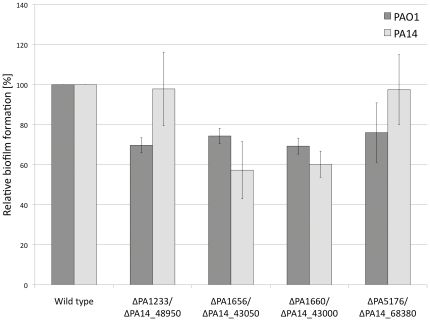
Relative biofilm formation of *P. aeruginosa* PAO1 and PA14 strains and their transposon mutants evaluated by crystal violet assay. An error bars were calculated from eight replicates and two independent experiments.

**Figure 4:**
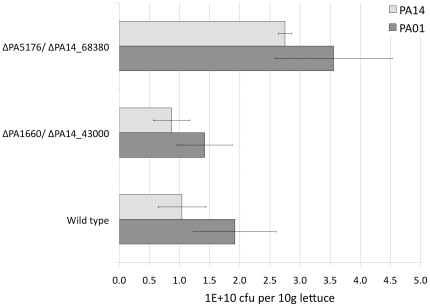
Virulence of the *P. aeruginosa* PAO1 and PA14 wild type strains compared to their transposon mutants, PA5176 (PA14_68380) and PA1660 (PA14_43000) in the lettuce leaf assay. The number of bacterial cells (as colony forming units, cfu) present in 10 g of lettuce midrib 3 days post injection is shown. Error bars were calculated from three independent experiment.

Among the genes expressed in all conditions, we filtered those that were expressed in one condition only and absent in the others. We obtained a highly specific list of genes expressed only in one condition in our experiments limited to only three up to eleven in number. Those genes are given in SI, figures S2, S3, S4, S5, S6. We tested representative unique genes from two amenable conditions, the plant infection and the biofilm. We used the transposon mutants of *P. aeruginosa* PAO1 and PA14 strains [31], [32] to verify if the filtered genes were indeed important for one condition and did not influence growth in other conditions. Figure 3 shows the biofilm formation assay results with mutants of the genes predicted to be part of the signature of biofilm formation (Figure S4), namely: PA1656 (being PA14_43050 the PA14 strain homologue of this mutant), PA1660 (PA14_43000), encoding part of a putative type VI secretion system and the hypothetical gene PA1123 (PA14_49850), as well as a gene specifically expressed in the plant infection PA5176 (PA14_68380). The mutants encoding the putative type VI secretion system were defective in the biofilm formation of up to 42% in the wild type strains (p-value<0.05, t values between 6.8–14.9). The mutant PA1123 was significantly impaired in its biofilm formation capability (p<0.05, t = 8.9), whereas PA14_49850 showed no significant change. Similarly, the mutant PA5176, which was shown to be specifically expressed in the plant infection, was slightly affected in its biofilm formation ability (*p* = 0.045, t = 2.65), whereas its counterpart PA14_68380 in the PA14 showed essentially no change. We also performed plant infection assays with representative mutants of genes predicted to be part of the: (i) plant infection signature, the conserved hypothetical PA5176 (PA14_68380) and (ii) biofilm formation signature, the gene from putative type VI secretion encoding operon PA1660 (PA14_43000) (Figure S6). The results show that the mutant strain PA5176 was twice more effective in plant infection (p-value<0.05, t = 5.1) than the wild type PAO1 (Fig. 4). The PA14 mutant PA14_68380 (PA5176 homologue) was almost three times more effective in proliferation in lettuce leaf (p<0.05, t = 8.6). When testing the biofilm signature mutant PA1660 (PA14_43000) in plant infection there was no significant difference in comparison to wild type strains (Figure 4). These results support the hypothesis formulated above on the genetic signatures for different infection conditions, which are largely independent of the strain variability and more strongly determined by the specific environments.

We had a greater number of relevant mutants available in the PAO1 transposon library than in that of PA14 and we thus extended our testing with them. The selected biofilm signature genes, namely PA0263 – *hcpC* coding for a hemolysin co-regulated protein; PA1661 – another member of the putative type VI secretion system; and the hypothetical gene PA3906 were all impaired biofilm formation (*p*<0.05, t values between 10.2–15.4), but had no significant change in the plant infection (Figures S7 and S8). The plant infection specific gene PA0734 did not show significant difference in either plant or biofilm infection (Figures S7 and S8).

We thus showed that the predicted condition-specific genes are indeed important under these conditions for both PAO1 and PA14. The putative type VI secretion system here tested is yet unknown and will require further elucidation but it is clear from our results that it is required for biofilm formation on the abiotic surface. For the plant infection per se, this system is not needed. Quite interestingly, however, the mutant of the gene PA5176 (PA14_68380), which belongs to the plant infection signature, was more virulent to plants than the wild type counterparts. It is uncertain at this stage why would it have increased virulence, but it is clearly involved in plant infection. Sequence analysis of the protein encoded by this gene indicates that it has a conserved NUDIX hydrolase domain, typical of ADP-ribose hydrolases, which hydrolyze the ADP-ribose to AMP and ribose- 5-phosphate and is believed to be involved in maintaining the level of ADP-ribose in the cell. Some enzymes from this family have the ability to degrade potentially mutagenic, oxidized nucleotides whereas other control the levels of metabolic intermediates and signaling compounds [33]. Further research will have to be done to unravel the function and role of this gene in virulence.

Regardless of the minor differences between the strains, the joint expression profile analysis of these and the “control” conditions underscores the proposition that each “niche signature” consists of a “core” gene set common to all conditions (e.g. those responsible for DNA replication, ribosomal structure, see house keeping genes in SI Table S8), and a set of niche specific genes whose wiring support proliferation of *P. aeruginosa* in those various environments (See Tables S2, S3, S4, S5, S6). This observation hints at a “modular” structure of the transcriptional programs in this bacterium, which underscores its ability to thrive ubiquitously in the environment and opportunistic potential.

### Conclusions

We report here the global analysis of *P. aeruginosa* gene expression under *in vitro* and *in vivo* conditions. We identified signature genes encoding proteins expressed specifically under each setting tested. Our study is also the first report of *in vivo* measurement of global gene expression by *P. aeruginosa* upon burn wound infection and sheds light on the genetic programs used by the pathogen under these conditions. One of the most valuable insights herein is that the main stress *P. aeruginosa* has to overcome is iron limitation. Although this is common to other microbial infections, it had not been previously shown for humans infected by *P. aeruginosa*, thereby opening possible new avenues for combating this threatening, ubiquitous pathogen. We also compared, for the first time, the *in vivo* behavior of genetically distinct *P. aeruginosa* strains under different infection conditions, namely burn wounds, solid tumors and lettuce, as well as under *in vitro* conditions. We showed for our set of strains that the *P. aeruginosa* core genome encodes a pool of genetic traits that are activated similarly under particular conditions regardless of the strain-to-strain genetic variability. The astonishingly high number of hypothetical genes that were differentially expressed only under *in vivo* conditions underscores the importance of *in vivo* studies as compared to *in vitro* studies for the understanding of the infection processes. These genes are obvious candidates for subsequent functional characterization and for research aiming at determining their function within the infection processes. Altogether, our data suggest that *P. aeruginosa* has a wide variety of conserved virulence mechanisms at its disposal, enabling this bacterium to effectively adapt to and survive in vastly divergent environments.

While focusing on *P. aeruginosa*, the insights obtained in this work point to general principles – the combination of general and niche-specific signatures, and the importance of *in vivo* expression profiling - that are likely to be relevant for the global understanding of pathogenicity in opportunistic infections.

## Materials and Methods

### Ethics Statement

Samples from burn wound patients were collected during regular wound debridement. The sampling was performed as a part of standard care procedure in the Clinic. The sample provided for this research was subtracted from the samples collected for routine microbiological tests, which are made on the regular basis, therefore no additional procedures were carried on the patients. Three samples were taken for preliminary studies in order to evaluate the possibility to extract bacterial RNA. Verbal informed consent for the isolation of bacterial RNA from those routine patient samples was considered sufficient and was obtained from patients in presence of two members of the clinic. The verbal informed consent was documented by the clinic member. Ultimately, for further research and prior to sample analysis the sampling procedure was duly approved by the Ethical Board of Silesian Medical Academy, which also suggested and provided the form for written informed consent in future.

### Bacterial strains

Three independent *P. aeruginosa* strains were isolated from 3 different burn wound patients with a monoclonal infection of *P. aeruginosa* being treated at the Centre for Burn Treatment (CLO) in Siemianowice Śląskie, Poland. Only patients with a clinically confirmed *P. aeruginosa* burn wound infection were chosen for sample collection. Liquid exudates were taken from the burn wound surface using swabs and kept at room temperature in transport medium. The 3 *P. aeruginosa* isolates herein named PBCLOp10, PBCLOp11 and PBCLOp17 were verified to be unrelated clonal complexes by binary array genotyping [10]. The wild type *P. aeruginosa* strain PAO1 and its transposon mutants (PW2332, PW3030, PW3947, PW3955, PW3957, PW7598, PW9706) were obtained from Department of Genome Sciences at the University of Washington [31]. The wild type *P. aeruginosa* PA14 and its transposon mutants (IDs – 31367, 23266, 33144, 41430, 36721) were obtained from the mutant library from the Harvard Medical School (http://ausubellab.mgh.harvard.edu/cgi-bin/pa14/home.cgi) [32].

### *In vitro* bacterial growth

The 3 *P. aeruginosa* strains were grown separately in 100 ml LB broth at 37°C and at 160 rpm until a 1∶10 dilution measures an optical density of 0.6 at 600 nm (equating to early stationary phase). Afterwards, cells were harvested for RNA isolation as described below. For biofilm growth, overnight pre-inoculum of each *P. aeruginosa* strain was diluted to an optical density of 0.05 at 600 nm in 10% LB medium, where plastic Permanox® slides (Nunc, Rochester, NY, USA) were suspended into 100 ml of the diluted culture. Flasks were kept at 37°C for 24 h without shaking. Once biofilm formation was observed on the plastic slides, slides were then washed 2 times in fresh LB medium then immersed into RNAprotect Bacteria Reagent (Qiagen, Hilden, Germany) and biofilms removed by a cell scraper. Cells were then harvested for RNA as described below.

### Biofilm formation assay

A modified crystal violet microtiter plate test, as described before [34], was used to asses the biofilm formation of wild type *P. aeruginosa* PAO1/PA14 and its transposon mutants. Briefly, overnight LB cultures were diluted to an A_600_ = 0.05 and 100 µl was inoculated in 96-well microtiter plate (PVC, BD Biosciences). Each strain was inoculated in 8 wells and the experiment was repeated with exchanging the position of strains on the plate. After 24 h incubation the cultures were withdrawn and wells washed with H_2_O. 150 µl of crystal violet staining solution (0.1% m/v H_2_O) was added to the wells and the plate was incubated for 30 min at room temperature. After incubation the staining solution was removed, wells washed with H_2_O, and left to dry. The crystal violet was resolubilized with 200 µl Ethanol (95%) for 30 min at RT. The 150 µl of Ethanol solution was transferred to fresh plate and the absorbance was measured at the 550 nm.

### Burn wound sample collection

From the same 3 burn wound patients (described above), liquid exudates were also taken from each burn wound surface prior to the wound cleansing, which is a regular treatment, using sterile forceps and immediately transferred to vials containing RNAprotect Bacteria Reagent. Each sample containing the buffer was vortexed, incubated for 10–15 min at room temperature and centrifuged for 15 min at 4000 *x g*. The supernatant was discarded and the pellet immediately frozen at −20°C. Frozen samples were transported on dry ice and further stored at −70°C until RNA extraction.

### Murine tumor infection

Six-week-old female BALB/c mice were purchased from Harlan (Germany). All animal experiments were approved by the appropriate ethical board (approval ID 33.9.42502-04-050/09 obtained from Niedersächsisches Landesamt für Verbraucherschutz und Lebensmittelsicherheit, Oldenburg, Germany). Cells of the colon adenocarcinoma cell line CT26 (ATCC CRL-2638) were grown in IMDM medium (Gibco, Karslruhe, Germany) supplemented with 10% (v/v) fetal calf serum, 2 mM L-glutamine and 10 mM HEPES. Prior to their injection into mice, CT26 cells were trypsinized, washed and finally resuspended in phosphate-buffered saline (PBS). Cells at a concentration of 10^6^ were injected subcutaneously in the abdomen. Mice bearing tumors of approximately 4–6 mm diameter in size were intravenously injected with 5×10^6^ CFU of *P. aeruginosa* suspended in PBS. Three days post-infection (p.i.) mice were necrotized and infected tumors prepared for stabilization of bacterial RNA in the following way: the tumors were removed and excised into 2–4 pieces, put onto nylon filters for cell culture (70 µm pore size) and suspended in 2 ml of RNAproctect Bacteria Reagent in 2 cm petri dishes. The tumor was spent through the membrane with a sterile spatula. The resulting mixture in the RNAprotect reagent was collected and centrifuged for 5 min at maximum speed 16,000 *x g*. The pellet was immediately frozen at −70°C for further RNA extraction (described below).

### Lettuce infection model

The protocol for using romaine lettuce leaves as a model of *P. aeruginosa* infection was performed as previously described [35], [36]. *P. aeruginosa* strains were grown aerobically overnight at 37°C in LB broth, washed twice with 10 mM MgSO_4_, and diluted in sterile MgSO_4_ to a cell concentration of 1×10^8^ CFU/ml. Lettuce leaves (Mini-Roma lettuce purchased commercially) were prepared by washing with distilled H_2_O containing 0.1% bleach (sodium hypochlorite) followed by additional wash pure distilled H_2_O. Lettuce mid-ribs were inoculated with 10 µl of bacterial suspension at a concentration of 1×10^8^ CFU/ml (corresponding to ∼1×10^6^ bacteria in total) by injecting the end of the plastic pipette tip into the rib and then all leaves were placed in plastic containers containing Whatman paper moistened with 10 mM MgSO_4_. Lettuce was incubated at 37°C or 30°C, and symptoms were monitored daily over the course of 5 days. As a negative control, lettuce leaves were inoculated with 10 mM MgSO_4_. A separate lettuce leaf was used for each strain. The experiments were repeated three times. Five days p.i. a 2 cm^2^ piece from the original place of injection was excised from the leaf and immersed into 3 ml RNAprotect Bacteria Reagent. The sample was vortexed for 30 sec and the solid parts of the plant tissue discarded. The resulting mixture was incubated for 5 min at room temperature and centrifuged for 15 min at 4000 *x g*. The supernatant was discarded and the pellet frozen at −70°C for further RNA extraction performed as described below. For the PAO1 mutant infection assay the number of bacterial cells in the midrib were determined by CFU after 3 days of the incubation period.

### Total RNA isolation

Analysis of gene expression of the pathogen directly at the infection site is limited by various factors associated with RNA extraction. The RNA extracted from clinical samples will most likely be a mixture of bacterial RNA with host RNA. Prior to RNA extraction samples were thawed on ice. RNA isolation was performed using RNAeasy Mini Kit (Qiagen) according to manufacturer's instructions with some minor modifications: samples were treated with 600 µl of TE buffer containing 1 mg/ml Lysozyme, incubated for 10 min with periodic vortexing every 2 min for 15 sec. Then, 1050 µl of RLT buffer containing 1% β-mercaptoethanol was added. The sample was vortexed and centrifuged for 2 min at maximum speed (16000 *x g*). The supernatant was transferred to a fresh 15 ml tube and 750 µl of absolute ethanol was added. The sample was loaded onto a spin column where the DNA was digested using RNase-free DNase I. The RNA was then eluted twice from each column with 50 µl and then 30 µl of RNase-free water. Eluted RNA was treated a second time with DNase I to ensure that all traces of genomic DNA were removed. The isolated RNA was stored at −70°C. The yield of the isolated RNA was measured by light absorption at 260 nm in an Eppendorf photometer. Integrity and purity was checked by formaldehyde gel electrophoresis or by 2100 BioAnalyzer (Agilent Technologies, Santa Clara, CA, USA).

### Bacterial RNA enrichment

Since the extracted RNA from the *in vivo* samples contained both bacterial and eukaryotic RNA (i.e. human or mouse), the samples had to be enriched for bacterial RNA (Fig. S1). This was achieved with the MicrobEnrich Kit® (Ambion, Austin, TX, USA). The basis of the kit is to hybridize the eukaryotic ribosomal RNA and the messenger RNA to the magnetic beads. Hybridization occurs between specific 18S and 28S rRNA regions and polyA tails of eukaryotic mRNA. Since there are reports that bacterial mRNA including that of *P. aeruginosa* possesses polyadenylated (polyA) tails to some extent [37] all of the samples including the controls were treated for enrichment.

### RNA amplification

One of the main bottlenecks in the transcriptomic analysis of host-pathogen interactions is the amount of bacterial RNA that can be isolated from the sample taken from the site of infection. The well established *P. aeruginosa* GeneChip® from Affymetrix (Santa Clara, CA, USA) requires 10 µg of RNA per microarray. Even with burn wound infections, where the amount of bacterial cells is relatively large this amount was never obtained. A promising way to overcome this obstacle was with the use of bacterial RNA amplification. This was performed using the MessageAmp Bacteria Kit (Ambion). The procedure consists of the following steps: i) total enriched bacterial RNA is treated with an enzyme polyadenylation polymerase to produce polyA tails, ii) single stranded cDNA is produced using reverse transcriptase and oligo dT primers, iii) second strain cDNA is produced, and iv) double stranded cDNA serves as a template for *in vitro* transcription using T7 RNA polymerase and T7 oligonucleotides. During the *in vitro* transcription reaction modified nucleotides were used: biotin-11-CTP (PerkinElmer Life Sciences, Waltham, MA, USA) and biotin-16-UTP (Roche Applied Science, Basel, Switzerland). As a result we obtained antisense biotinylated RNA, ready to use for GeneChip® hybridization.

### Microarray hybridization

The use of amplified RNA is common with eukaryotic microarrays, but the original procedure for *P. aeruginosa* GeneChip® (Affymetrix) was for single stranded terminally labeled cDNA. Comparing to the standard procedure (without amplification), amplifying RNA for microarray analysis does not change the final outcome as previously reported [38]. The hybridization and washing steps were performed in the Affymetrix Array facility at Helmoholtz Centre for Infection Research (HZI) Braunschweig (Dr. Robert Geffers). Since the RNA had been amplified, some changes were introduced to the original Affymetrix protocol, such as that the RNA was fragmented using 5x fragmentation buffer instead of DNaseI treatment. The total amount of amplified and fragmented RNA used per chip was 7.5 µg. The process of sample preparation for each chip was as follows: the RNA from each single condition was pooled together after the enrichment; amplification step was performed on each condition; amplified RNA was hybridized in duplicate onto microarray chips in order to have technical replicates. Initial steps of data analysis were done at the Array facility using Affymetrix Microarray Suite Software 5.0 with default parameters. Once raw data files of scanned pictures were obtained further bioinformatic analysis was made as described below.

### Microarray data normalisation

Data normalization and calculation of differential expression was performed with software from Bioconductor microarray analysis suite [39]. The quality of all chips was assessed by fitting a linear model to the probe level data using the function “fitPLM” from the “affyPLM” package. Subsequently, the distribution (boxplots) of RLE (Relative Log Expression) and NUSE (Normalised Unscaled Standard Errors) was manually analyzed. Expression values were computed using the “Robust Multichip Average” algorithm [40].

### Differential Expression

As the number of replicates was low, the “Rank Products” algorithm was used to identify differentially expressed genes [41]. It has been shown that this algorithm performs well when the number of replicates is low [42]. The algorithm addresses the multiple testing problems by calculating for every gene an estimate of percentage of false-positives (pfp), if this gene and all genes with lower pfp would be considered as significantly differentially expressed. Thus, it is an estimate of False Discovery Rate (FDR). The value of 0.05 was accepted as a cut-off for pfp. The computations were performed using function Rpadvance from the “RankProd” package. A list of significantly differentially expressed genes was created for every pair of growth conditions (burn wound, lettuce, mouse tumor, planktonic and biofilm). Microarray data discussed here is MIAME compliant and have been deposited to the National Center for Biotechnology Information's Gene Expression Omnibus (GEO, http://www.ncbi.nlm.nih.gov/geo/) and are accessible through GEO series accession number GSE23007.

### Hierarchical clustering of differentially expressed genes

Of all genes that showed a significant change of expression in at least one pairwise comparison between the experiments (1734 genes), hierarchical clustering was performed using the 1-correlation as the distance measure and clustering performed using the “hclust” function of the R software suite, http://www.r-project.org/. Subsequently, the outcome of the clustering was used to identify groups of genes that followed a similar pattern in changes to expression, where 213 groups were detected using a cutoff of 0.15. The expression values were averaged within each of the groups to form for each of them a group-wide expression pattern. Finally, the hierarchical clustering was performed on the groups, following the same procedure as previously described. The dendrogram, together with the heatmap composed of the group-wide expression values were plotted.

### Specific gene signatures

To identify signature genes a set of five linear models was fit for every gene. Single model assumed that the expression change a gene occurs only in single condition (therefore five models for five conditions). As signature genes for a particular condition those were selected whose absolute value of linear coefficient for this condition exceeded 1.8 (the values of all five coefficients of a particular gene summed to 0). This value was chosen empirically, in order to achieve a reasonable number of signature genes. Afterwards, the expression patterns of chosen genes were inspected visually.

### Ordination of the global gene expression profiles of *P. aeruginosa* strains within each condition

Each of the three independent *P. aeruginosa* strains at each of the five studied conditions were ordinated using principal coordinate analysis (PCO). A permutational multivariate analysis of variance (PERMANOVA) was performed to determine the statistical significance of the effect of condition on the global transcriptional profile of independent strains as observed in the PCO plot [43], [44]. These routines were performed using PRIMER (v.6.1.6, PRIMER-E, Plymouth Marine Laboratory, UK) [45], [46]. From the gene expression data matrix comprising those 1734 differentially expressed genes, a resemblance-matrix was generated using Euclidean distance by comparing the expression of each gene in regards to every pairwise combination of strains at each condition (burn wound infection, mouse tumor infection, lettuce infection, biofilm and planktonic growth). Euclidean distance is a widely used and accepted measure of distance in multivariate data such as gene expression data [47]. PCO ordination was used to reduce high dimensional data into low dimensional space and is equivalent to principal component analysis (PCA) when Euclidean distance is the distance algorithm chosen. PERMANOVA was performed according to a one-way experimental design. For one-way PERMANOVA, an exact *P*-value was generated using unrestricted permutation of raw data, where a Pseudo-*F* and corresponding *p*-value first report whether there are any overall differences in the gene expression between conditions. If there are, then the Pseudo-*F* statistic and generated *p*-values are reported for each pair of conditions. Pair-wise tests from PERMANOVA were also performed on unrestricted permutation of raw data. However, in the pairwise PERMANOVA, the total number of unique permutations when comparing the 3 strains across a pair of conditions was 10 and since the number of unique values under permutations was very low, the resulting *p*-values for each corresponding t-value can not be any smaller than 0.1. Thus, when only low unique values in the permutation distribution were available, asymptotical Monte Carlo *p*-values were generated instead of permutational *p*-values (9999 Monte Carlo samples). The ability to generate significance tests for small sample sizes made this approach powerful. That is, PERMANOVA seemed to be more powerful than other classical multivariate analyses allowing partitioning of variance components [48]. The conditions were considered significantly different if the *p*-value falls <0.05.

## Supporting Information

Table S1 **Antibiotic resistance patterns of clinical isolates from burn wounds compared to known laboratory strains** ***P. aeruginosa*** **PAO1 and PA14. R = Resistant, S = Susceptible and I = Intermediate. R in bold represents the result different than in the wild type strains.**
(PDF)

Table S2 **Gene signature of** ***P. aeruginosa*** **under** ***in vivo*** **conditions in burn wound infections.**
(PDF)

Table S3 **Gene signature of** ***P. aeruginosa*** **under** ***in vivo*** **conditions in lettuce infection.**
(PDF)

Table S4 **Gene signature of** ***P. aeruginosa*** **under** ***in vivo*** **conditions in mouse tumor infection.**
(PDF)

Table S5 **Gene signature of** ***P. aeruginosa*** **under** ***in vitro*** **conditions in biofilm growth.**
(PDF)

Table S6 **Gene signature of** ***P. aeruginosa*** **under** ***in vitro*** **conditions in planktonic growth.**
(PDF)

Table S7 **Differentially expressed** ***P. aeruginosa*** **genes from the** ***in vivo*** **burn wound infections in comparison to planktonic or biofilm** ***in vitro*** **growth as well as to previous studies of iron starvation response of** ***P. aeruginosa*** **PAO1 strain** ***in vitro.***
(PDF)

Table S8 **Housekeeping genes.** The genes with a standard error of probe values below 0.05 and a signal intensity of at least category 3 with 4 being the average signal intensity among all microarrays were defined as housekeeping genes.
(PDF)

Table S9 **Pairwise comparison of the genes present in the specific clusters.** Each condition, bw – burn wound infection, tu – tumor infection, pl – plant infection, bf – biofilm growth and lb – planktonic growth, was compared with each other. If the gene was differentially regulated in the comparison the “+” or “−” was given. For example if the gene in the bw/bf comparison has “+”, that means the gene was upregulated in burn wound infection as compared to the biofilm growht. Similarly in case of the “−” the gene would be downregulated in the burn wound as compared to the biofilm. The genes mentioned in the manuscript main text are highlighted in bald.
(PDF)

Figure S1 **Agilent Bioanalyzer results of bacterial RNA enrichment from sample PBCLOp10.** A) Sample before enrichment, peaks from bacterial ribosomal RNA (16S and 23S) are seen together with eukaryotic ribosomal RNA (18S and 28S). B) Sample after enrichment, only bacterial ribosomal signals are detected.
(PDF)

Figure S2 **The genes that were expressed exclusively under** ***in vivo*** **burn wound infections.** Gene PA0707 (*toxR*) encoding the regulator activating expression of *toxA*, which codes for Exotoxin A (Hamood and Iglewski 1990). Genes PA4835-6 and PA4836 mentioned above also belong to this cluster. Other burn wound specific genes were: PA3598 encoding a conserved hypothetical protein predicted to be N-carbamoylputrescine amidase, which catalyzes the hydrolysis of N-carbamoylputrescine to putrescine. It represents the final step of the arginine decarboxylase pathway of putrescine biosyntheseis operating in some plant and bacterial species; PA4172 encoding exodeoxyribonuclease III involved in DNA repair due to oxidative/nitrosative stress; gene *pyrQ* (*pyrC2*) (PA5541) encoding dihydroorotase involved in pyrimidine metabolism; PA5540 encoding carbonic anhydrase related protein. Lastly, PA4390 passed through the stringent filtering process encoding a hypothetical protein.
(PDF)

Figure S3 **The genes that were expressed exclusively under planktonic** ***in vitro*** **conditions.** PA1178 – encoding OprH protein and PA1180 - PhoQ – two component system responsible for sensing Mg limitation. PA0113 - probable cytochrome c oxidase assembly factor. PA2566 – pyridine nucleotide-disulfide family oxidoreductase. PA4297 – the Flp assembly machinery. PA4302 and PA4304 (*tadA*, *rcpA*) are part of pathway encoding for Type IV pillus assembly, where the gene PA4297 is also required. PA4648 – unknown with export signal sequence. PA5208 – conserve hypothetical phosphate transport regulator (distant homolog of PhoU).
(PDF)

Figure S4 **The genes that were expressed exclusively under the biofilm** ***in vitro*** **conditions.** PA0263 – *hcpC* - encoding hemolisin co-regulated protein. PA1656, 59, 60, 61 – encoding putative type VI secretion system. PA4494 – putative two component system. PA5490 - cytochrome c4 precursor. PA1123 and PA3906 hypothetical unknown genes.
(PDF)

Figure S5 **The genes that were expressed exclusively under the** ***in vivo*** **tumor infection.** PA0415 – *chpC* - encoding putative chemotaxis protein. PA0518 – *nirM*, cytochrome c-551 precursor. PA1195 - N-Dimethylarginine dimethylaminohydrolase (Amino acid transport and metabolism).
(PDF)

Figure S6 **The genes that were expressed exclusively under the** ***in vivo*** **plant infections.** PA0734 – encoding hypothetical unknown protein. PA1060 - predicted permease, DMT superfamily. PA1856 – encoding probable cytochrome oxidase subunit. PA2031 – hypothetical unknown. PA2663 encoding membrane protein of unknown function. PA2664 – *fhp*, flavohemoprotein, aerobic nitric oxide detoxification. PA2847 – predicted permease. PA4147 – *acoR*, transcriptional activator of acetoin/glycerol. PA5176 – conserved hypothetical gene.
(PDF)

Figure S7 ***P. aeruginosa*** **PAO1 wild type and mutant strains biofilm formation evaluation performed by crystal violet assay.** An error bars were calculated from eight replicates and two independent experiments.
(PDF)

Figure S8 **Virulence of the** ***P. aeruginosa*** **PAO1 wild type and mutant strain in the lettuce leaf assay.** The number of bacterial cells (as colony forming units, cfu) present in 10 g of lettuce midrib 3 days post injection is shown. Error bars were calculated from three independent experiments.
(PDF)

## References

[1] Stover CK, Pham XQ, Erwin AL, Mizoguchi SD, Warrener P (2000). Complete genome sequence of *Pseudomonas aeruginosa* PAO1, an opportunistic pathogen.. Nature.

[2] Bielecki P, Glik J, Kawecki M, Martins dos Santos VAP (2008). Towards understanding *Pseudomonas aeruginosa* burn wound infections by profiling gene expression.. Biotechnology Letters.

[3] Man-Wah T, Laurence GR, Jeffrey AS, Ronald GT, Frederick MA (1999). *Pseudomonas aeruginosa* killing of *Caenorhabditis elegans* used to identify *P. aeruginosa* virulence factors.. Proceedings of the National Academy of Sciences of the United States of America.

[4] D'Argenio DA, Gallagher LA, Berg CA, Manoil C (2001). Drosophila as a model host for *Pseudomonas aeruginosa* infection.. Journal of bacteriology.

[5] Rahme L, Stevens E, Wolfort S, Shao J, Tompkins R (1995). Common virulence factors for bacterial pathogenicity in plants and animals.. Science.

[6] He J, Baldini R, Deziel E, Saucier M, Zhang Q (2004). The broad host range pathogen *Pseudomonas aeruginosa* strain PA14 carries two pathogenicity islands harboring plant and animal virulence genes.. Proceedings of the National Academy of Sciences.

[7] Lee D, Urbach J, Wu G, Liberati N, Feinbaum R (2006). Genomic analysis reveals that *Pseudomonas aeruginosa* virulence is combinatorial.. Genome Biology.

[8] Mathee K, Narasimhan G, Valdes C, Qiu X, Matewish JM (2008). Dynamics of *Pseudomonas aeruginosa* genome evolution.. Proceedings of the National Academy of Sciences.

[9] Rahme L, Tan M-W, Le L, Wong S, Tompkins R (1997). Use of model plant hosts to identify *Pseudomonas aeruginosa* virulence factors.. Proceedings of the National Academy of Sciences.

[10] Wiehlmann L, Wagner G, Cramer N, Siebert B, Gudowius P (2007). Population structure of *Pseudomonas aeruginosa*.. Proc Natl Acad Sci U S A.

[11] Stewart RMK, Wiehlmann L, Ashelford KE, Preston SJ, Frimmersdorf E (1003).

[12] Ochsner UA, Wilderman PJ, Vasil AI, Vasil ML (2002). GeneChip expression analysis of the iron starvation response in *Pseudomonas aeruginosa*: identification of novel pyoverdine biosynthesis genes.. Mol Microbiol.

[13] Baecker PA, Greenberg EA, Preiss J (1986). Biosynthesis of bacterial glycogen. Primary structure of Escherichia coli 1,4-alpha-D-glucan:1,4-alpha-D-glucan 6-alpha-D-(1, 4-alpha-D-glucano)-transferase as deduced from the nucleotide sequence of the *glg*B gene.. Journal of Biological Chemistry.

[14] Weir TL, Stull VJ, Badri D, Trunck LA, Schweizer HP (2008). Global Gene Expression Profiles Suggest an Important Role for Nutrient Acquisition in Early Pathogenesis in a Plant Model of *Pseudomonas aeruginosa* Infection.. Appl Environ Microbiol.

[15] Nishijyo T, Haas D, Itoh Y (2001). The CbrA–CbrB two-component regulatory system controls the utilization of multiple carbon and nitrogen sources in *Pseudomonas aeruginosa*.. Molecular Microbiology.

[16] Li W, Lu C-D (2007). Regulation of Carbon and Nitrogen Utilization by CbrAB and NtrBC Two-Component Systems in *Pseudomonas aeruginosa*.. J Bacteriol.

[17] Sonnleitner E, Abdou L, Haas D (2009). Small RNA as global regulator of carbon catabolite repression in *Pseudomonas aeruginosa*.. Proceedings of the National Academy of Sciences.

[18] Yahr TL, Wolfgang MC (2006). Transcriptional regulation of the *Pseudomonas aeruginosa* type III secretion system.. Molecular Microbiology.

[19] Cryz SJ, Pitt TL, Furer E, Germanier R (1984). Role of lipopolysaccharide in virulence of *Pseudomonas aeruginosa*.. Infect Immun.

[20] Dasgupta T, de Kievit TR, Masoud H, Altman E, Richards JC (1994). Characterization of lipopolysaccharide-deficient mutants of *Pseudomonas aeruginosa* derived from serotypes O3, O5, and O6.. Infect Immun.

[21] Vallet I, Olson JW, Lory S, Lazdunski A, Filloux A (2001). The chaperone/usher pathways of *Pseudomonas aeruginosa*: identification of fimbrial gene clusters (cup) and their involvement in biofilm formation.. Proceedings of the National Academy of Sciences of the United States of America.

[22] Singh PK, Schaefer AL, Parsek MR, Moninger TO, Welsh MJ (2000). Quorum-sensing signals indicate that cystic fibrosis lungs are infected with bacterial biofilms.. Nature.

[23] Vallet-Gely I, Sharp J, Dove S (2007). Local and Global Regulators Linking Anaerobiosis to cupA Fimbrial Gene Expression in *Pseudomonas aeruginosa*.. J Bacteriol.

[24] Schobert M, Tielen P (2010). Contribution of oxygen-limiting conditions to persistent infection of *Pseudomonas aeruginosa*.. Future Microbiology.

[25] Schreiber K, Boes N, Eschbach M, Jaensch L, Wehland J (2006). Anaerobic survival of *Pseudomonas aeruginosa* by pyruvate fermentation requires an Usp-type stress protein.. Journal of bacteriology.

[26] Rumbaugh KP, Griswold JA, Iglewski BH, Hamood AN (1999). Contribution of quorum sensing to the virulence of *Pseudomonas aeruginosa* in burn wound infections.. Infect Immun.

[27] Whiteley M, Lee KM, Greenberg EP (1999). Identification of genes controlled by quorum sensing in *Pseudomonas aeruginosa.*. Proceedings of the National Academy of Sciences.

[28] Juhas M, Wiehlmann L, Huber B, Jordan D, Lauber J (2004). Global regulation of quorum sensing and virulence by VqsR in *Pseudomonas aeruginosa*.. Microbiology.

[29] Oberhardt MA, Puchalka J, Fryer KE, Martins dos Santos VAP, Papin JA (2008). Genome-Scale Metabolic Network Analysis of the Opportunistic Pathogen *Pseudomonas aeruginosa* PAO1.. J Bacteriol.

[30] Oberhardt MA, Goldberg JB, Hogardt M, Papin JA (2010). Metabolic Network Analysis of *Pseudomonas aeruginosa* during Chronic Cystic Fibrosis Lung Infection.. J Bacteriol.

[31] Jacobs MA, Alwood A, Thaipisuttikul I, Spencer D, Haugen E (2003). Comprehensive transposon mutant library of *Pseudomonas aeruginosa*.. Proceedings of the National Academy of Sciences.

[32] Liberati NT, Urbach JM, Miyata S, Lee DG, Drenkard E (2006). An ordered, nonredundant library of *Pseudomonas aeruginosa* strain PA14 transposon insertion mutants.. Proceedings of the National Academy of Sciences of the United States of America.

[33] McLennan A (2006). The Nudix hydrolase superfamily.. Cellular and Molecular Life Sciences.

[34] Stepanovic S, Vukovic D, Dakic I, Savic B, Svabic-Vlahovic M (2000). A modified microtiter-plate test for quantification of staphylococcal biofilm formation.. Journal of Microbiological Methods.

[35] Filiatrault M, Picardo K, Ngai H, Passador L, Iglewski B (2006). Identification of *Pseudomonas aeruginosa* Genes Involved in Virulence and Anaerobic Growth.. Infect Immun.

[36] Starkey M, Rahme LG (2009). Modeling *Pseudomonas aeruginosa* pathogenesis in plant hosts.. Nat Protocols.

[37] Saravanamuthu S, von Gotz F, Salunkhe P, Chozhavendan R, Geffers R (2004). Evidence for Polyadenylated mRNA in *Pseudomonas aeruginosa*.. J Bacteriol.

[38] Francois P, Garzoni C, Bento M, Schrenzel J (2007). Comparison of amplification methods for transcriptomic analyses of low abundance prokaryotic RNA sources.. J Microbiol Methods.

[39] Gentleman RC, Carey VJ, Bates DM, Bolstad B, Dettling M (2004). Bioconductor: open software development for computational biology and bioinformatics.. Genome Biol.

[40] Irizarry RA, Hobbs B, Collin F, Beazer-Barclay YD, Antonellis KJ (2003). Exploration, normalization, and summaries of high density oligonucleotide array probe level data.. Biostatistics.

[41] Breitling R, Armengaud P, Amtmann A, Herzyk P (2004). Rank products: a simple, yet powerful, new method to detect differentially regulated genes in replicated microarray experiments.. FEBS Lett.

[42] Jeffery I, Higgins D, Culhane A (2006). Comparison and evaluation of methods for generating differentially expressed gene lists from microarray data.. BMC Bioinformatics.

[43] Anderson MJ (2001). A new method for non-parametric multivariate analysis of variance.. Austral Ecology.

[44] McArdle BH, Anderson MJ (2001). Fitting Multivariate Models to Community Data: a Comment On Distance-Based Redundancy Analysis.. Ecology.

[45] Clarke KR, Warwick RM (2001). Change in marine communities: An approach to statistical analysis and interpretation..

[46] Anderson MJ (2005). PERMANOVA: A FORTRAN Computer Program for Permutational Multivariate Analysis of Variance..

[47] Butte A (2002). The use and analysis of microarray data.. Nat Rev Drug Discov.

[48] Quin GP, Keough MJ (2002). Experimental Design and Data Analysis for Biologists..

