# Supplementary material for: In-Vivo Expression Profiling of Pseudomonas aeruginosa Infections Reveals Niche-Specific and Strain-Independent Transcriptional Programs
Source: PLoS One. 2011 Sep 12;6(9):e24235. doi: 10.1371/journal.pone.0024235 (PMC3171414; doi:10.1371/journal.pone.0024235)
Supplement: Table S1 — Antibiotic resistance patterns of clinical isolates from burn wounds compared to known laboratory strains P. aeruginosa PAO1 and PA14. R = Resistant, S = Susceptible and I = Intermediate. R in bold represents the result different than in the wild type strains. (PDF) [file pone.0024235.s001.pdf]

Table S1

Strain:	Beta lactams													Aminoglicosydes		Quinolones			Glycycylines	Sulfonamides
	Penicillins				Cephalosporins								Carbapenem							
	Ampicilin	Ampicillin/Sublactam	Piperacillin	Piperacillin/Tazobactam	Cefazolin	Cefuroxime	Cefuroxime Axetil	Cefoxitin	Cefpodoxime	Cefotaxime	Ceftazidime	Cefepime	Meropenem	Gentamycin	Tobramycin	Nalidixic Acid	Ciprofloxacin	Levofloxacin	Tigecycline	Trimethoprim*
PAO1	R	R	S	S	R	R	R	R	R	I	I	S	S	S	S	R	I	I	R	R
PA14	R	R	S	S	R	R	R	R	R	I	I	S	S	S	S	R	S	S	R	I
PBCLOp10	R	R	R	R	R	R	R	R	R	R	I	I	R	R	R	R	S	S	R	R
PBCLOp11	R	R	S	S	R	R	R	R	R	R	I	I	S	I	S	R	**R**	I	R	R
PBCLOp17	R	R	**R**	**R**	R	R	R	R	R	R	**R**	**R**	S	**R**	**R**	R	**R**	**R**	R	R

*Full name: Trimethoprin/Sulfamethoxazole
