# Supplementary material for: In-Vivo Expression Profiling of Pseudomonas aeruginosa Infections Reveals Niche-Specific and Strain-Independent Transcriptional Programs
Source: PLoS One. 2011 Sep 12;6(9):e24235. doi: 10.1371/journal.pone.0024235 (PMC3171414; doi:10.1371/journal.pone.0024235)
Supplement: Table S2 — Gene signature of P. aeruginosa under in vivo conditions in burn wound infections. (PDF) [file pone.0024235.s002.pdf]

Table S2

Locus ID	Gene name	Product name
PA0059	*osmC*	osmotically inducible protein OsmC
PA0060		conserved hypothetical protein
PA0102		probable carbonic anhydrase
PA0104		hypothetical protein
PA0266	*gabT*	4-aminobutyrate aminotransferase
PA0320		conserved hypothetical protein
PA0354		conserved hypothetical protein
PA0355	*pfpI*	protease PfpI
PA0376	*rpoH*	sigma factor RpoH
PA0423	*pasP*	PasP
PA0532		hypothetical protein
PA0553		hypothetical protein
PA0567		conserved hypothetical protein
PA0672	*hemO*	heme oxygenase
PA0707	*toxR*	transcriptional regulator ToxR
PA0763	*mucA*	anti-sigma factor MucA
PA0779		probable ATP-dependent protease
PA1134		hypothetical protein
PA1245		hypothetical protein
PA1320	*cyoD*	cytochrome o ubiquinol oxidase subunit IV
PA1323		hypothetical protein
PA1324		hypothetical protein
PA1500		probable oxidoreductase
PA1515	*alc*	allantoicase
PA1518		conserved hypothetical protein
PA1579		hypothetical protein
PA1596	*htpG*	heat shock protein HtpG
PA1870		hypothetical protein
PA1925		hypothetical protein
PA2000		probable CoA transferase, subunit B
PA2033		hypothetical protein
PA2034		hypothetical protein
PA2108		probable decarboxylase
PA2116		conserved hypothetical protein
PA2134		hypothetical protein
PA2135		probable transporter
PA2140		probable metallothionein
PA2141		hypothetical protein
PA2142		probable short-chain dehydrogenase
PA2143		hypothetical protein
PA2144	*glgP*	glycogen phosphorylase
PA2145		hypothetical protein
PA2146		conserved hypothetical protein
PA2147	*katE*	catalase HPII
PA2148		conserved hypothetical protein
PA2149		hypothetical protein
PA2150		conserved hypothetical protein
PA2151		conserved hypothetical protein
PA2152		probable trehalose synthase
PA2153	*glgB*	1,4-alpha-glucan branching enzyme

PA2157		hypothetical protein
PA2158		probable alcohol dehydrogenase (Zn-dependent)
PA2159		conserved hypothetical protein
PA2160		probable glycosyl hydrolase
PA2161		hypothetical protein
PA2164		probable glycosyl hydrolase
PA2165		probable glycogen synthase
PA2167		hypothetical protein
PA2168		hypothetical protein
PA2169		hypothetical protein
PA2170		hypothetical protein
PA2171		hypothetical protein
PA2172		hypothetical protein
PA2173		hypothetical protein
PA2176		hypothetical protein
PA2178		hypothetical protein
PA2180		hypothetical protein
PA2181		hypothetical protein
PA2182		hypothetical protein
PA2183		hypothetical protein
PA2184		conserved hypothetical protein
PA2187		hypothetical protein
PA2189		hypothetical protein
PA2190		conserved hypothetical protein
PA2192		conserved hypothetical protein
PA2383		probable transcriptional regulator
PA2384		hypothetical protein
PA2385	*pvdQ*	3-oxo-C12-homoserine lactone acylase PvdQ
PA2386	*pvdA*	L-ornithine N5-oxygenase
PA2393		probable dipeptidase precursor
PA2394	*pvdN*	PvdN
PA2405		hypothetical protein
PA2411		probable thioesterase
PA2412		conserved hypothetical protein
PA2413	*pvdH*	L-2,4-diaminobutyrate:2-ketoglutarate 4-aminotransferase, PvdH
PA2414		L-sorbosone dehydrogenase
PA2424	*pvdL*	PvdL
PA2485		hypothetical protein
PA2486		hypothetical protein
PA2634	*aceA*	isocitrate lyase AceA
PA2694		probable thioredoxin
PA3041		hypothetical protein
PA3042		hypothetical protein
PA3126	*ibpA*	heat-shock protein IbpA
PA3231		hypothetical protein
PA3273		hypothetical protein
PA3274		hypothetical protein
PA3283		conserved hypothetical protein
PA3407	*hasAp*	heme acquisition protein HasAp
PA3459		probable glutamine amidotransferase
PA3460		probable acetyltransferase
PA3461		conserved hypothetical protein
PA3540	*algD*	GDP-mannose 6-dehydrogenase AlgD

PA3541	*alg8*	alginate biosynthesis protein Alg8
PA3598		conserved hypothetical protein
PA3600		conserved hypothetical protein
PA3601		conserved hypothetical protein
PA3691		hypothetical protein
PA3692		probable outer membrane protein precursor
PA3731		conserved hypothetical protein
PA3795		probable oxidoreductase
PA3811	*hscB*	heat shock protein HscB
PA3812	*iscA*	probable iron-binding protein IscA
PA3814	*iscS*	L-cysteine desulfurase (pyridoxal phosphate-dependent)
PA3891		probable ATP-binding component of ABC transporter
PA3951		conserved hypothetical protein
PA4061		probable thioredoxin
PA4063		hypothetical protein
PA4065		hypothetical protein
PA4170		hypothetical protein
PA4171		probable protease
PA4172		probable nuclease
PA4175	*piv*	protease IV
PA4344		probable hydrolase
PA4345		hypothetical protein
PA4387		conserved hypothetical protein
PA4390		hypothetical protein
PA4394		conserved hypothetical protein
PA4467		hypothetical protein
PA4468	*sodM*	superoxide dismutase
PA4469		hypothetical protein
PA4470	*fumC1*	fumarate hydratase
PA4570		hypothetical protein
PA4661	*pagL*	Lipid A 3-O-deacylase
PA4738		conserved hypothetical protein
PA4739		conserved hypothetical protein
PA4761	*dnaK*	DnaK protein
PA4834		hypothetical protein
PA4835		hypothetical protein
PA4836		hypothetical protein
PA4837		probable outer membrane protein precursor
PA4838		hypothetical protein
PA4877		hypothetical protein
PA4896		probable sigma-70 factor, ECF subfamily
PA4936		probable rRNA methylase
PA5053	*hslV*	heat shock protein HslV
PA5054	*hslU*	heat shock protein HslU
PA5060	*phaF*	polyhydroxyalkanoate synthesis protein PhaF
PA5150		probable short-chain dehydrogenase
PA5340		hypothetical protein
PA5388		hypothetical protein
PA5481		hypothetical protein
PA5499	*np20*	transcriptional regulator np20
PA5501	*znuB*	permease of ABC zinc transporter ZnuB
PA5534		hypothetical protein
PA5535		conserved hypothetical protein

PA5536		conserved hypothetical protein
PA5537		hypothetical protein
PA5538	*amiA*	N-acetylmuramoyl-L-alanine amidase
PA5539		hypothetical protein
PA5540		hypothetical protein
PA5541	*pyrQ*	dihydroorotase
