# Supplementary material for: In-Vivo Expression Profiling of Pseudomonas aeruginosa Infections Reveals Niche-Specific and Strain-Independent Transcriptional Programs
Source: PLoS One. 2011 Sep 12;6(9):e24235. doi: 10.1371/journal.pone.0024235 (PMC3171414; doi:10.1371/journal.pone.0024235)
Supplement: Table S3 — Gene signature of P. aeruginosa under in vivo conditions in lettuce infection. (PDF) [file pone.0024235.s003.pdf]

Table S3

Locus ID	Gene name	Product name
PA0011		probable 2-OH-lauroyltransferase
PA0035	*trpA*	tryptophan synthase alpha chain
PA0036	*trpB*	tryptophan synthase beta chain
PA0201		hypothetical protein
PA0226		probable CoA transferase, subunit A
PA0276		hypothetical protein
PA0280	*cysA*	sulfate transport protein CysA
PA0281	*cysW*	sulfate transport protein CysW
PA0282	*cysT*	sulfate transport protein CysT
PA0283	*sbp*	sulfate-binding protein precursor
PA0284		hypothetical protein
PA0286	*desA*	delta-9 fatty acid desaturase, DesA
PA0296	*spuI*	probable glutamine synthetase
PA0298	*spuB*	probable glutamine synthetase
PA0385		hypothetical protein
PA0386		probable oxidase
PA0436		probable transcriptional regulator
PA0439		probable oxidoreductase
PA0441	*dht*	dihydropyrimidinase
PA0444		N-carbamoyl-beta-alanine amidohydrolase
PA0451		conserved hypothetical protein
PA0456		probable cold-shock protein
PA0472		probable sigma-70 factor, ECF subfamily
PA0473		probable glutathione S-transferase
PA0485		conserved hypothetical protein
PA0505		hypothetical protein
PA0547		probable transcriptional regulator
PA0608		probable phosphoglycolate phosphatase
PA0676		probable transmembrane sensor
PA0730		probable transferase
PA0734		hypothetical protein
PA0801		hypothetical protein
PA0802		hypothetical protein
PA0810		probable haloacid dehalogenase
PA0814		conserved hypothetical protein
PA0815		probable transcriptional regulator
PA0817		probable ring-cleaving dioxygenase
PA0837	*slyD*	peptidyl-prolyl cis-trans isomerase SlyD
PA0851		hypothetical protein
PA0862		hypothetical protein
PA0929		two-component response regulator
PA0936	*lpxO2*	lipopolysaccharide biosynthetic protein LpxO2
PA0995	*ogt*	methylated-DNA--protein-cysteine methyltransferase
PA1060		hypothetical protein
PA1075		hypothetical protein
PA1092	*fliC*	flagellin type B
PA1101	*fliF*	Flagella M-ring outer membrane protein precursor
PA1190		conserved hypothetical protein
PA1192		conserved hypothetical protein

PA1296		probable 2-hydroxyacid dehydrogenase
PA1377		conserved hypothetical protein
PA1440		hypothetical protein
PA1452	*flhA*	flagellar biosynthesis protein FlhA
PA1505	*moaA2*	molybdopterin biosynthetic protein A2
PA1626		probable major facilitator superfamily (MFS) transporter
PA1630		probable transcriptional regulator
PA1632	*kdpF*	KdpF protein
PA1651		probable transporter
PA1676		hypothetical protein
PA1677		conserved hypothetical protein
PA1730		conserved hypothetical protein
PA1775	*cmpX*	conserved cytoplasmic membrane protein, CmpX protein
PA1776	*sigX*	ECF sigma factor SigX
PA1778	*cobA*	uroporphyrin-III C-methyltransferase
PA1779		assimilatory nitrate reductase
PA1780	*nirD*	assimilatory nitrite reductase small subunit
PA1781	*nirB*	assimilatory nitrite reductase large subunit
PA1782		probable serine/threonine-protein kinase
PA1783	*nasA*	nitrate transporter
PA1800	*tig*	trigger factor
PA1814		hypothetical protein
PA1837		hypothetical protein
PA1838	*cysI*	sulfite reductase
PA1856		probable cytochrome oxidase subunit
PA1927	*metE*	5-methyltetrahydropteroyltriglutamate-homocysteine S-methyltransferase
PA1950	*rbsK*	ribokinase
PA1963		hypothetical protein
PA1984		probable aldehyde dehydrogenase
PA1998		probable transcriptional regulator
PA2019		Resistance-Nodulation-Cell Division (RND) multidrug efflux membrane fusion protein precursor
PA2030		hypothetical protein
PA2031		hypothetical protein
PA2045		conserved hypothetical protein
PA2092		probable major facilitator superfamily (MFS) transporter
PA2229		conserved hypothetical protein
PA2259	*ptxS*	transcriptional regulator PtxS
PA2260		hypothetical protein
PA2261		probable 2-ketogluconate kinase
PA2263		probable 2-hydroxyacid dehydrogenase
PA2306		conserved hypothetical protein
PA2322		gluconate permease
PA2380		hypothetical protein
PA2484		conserved hypothetical protein
PA2491		probable oxidoreductase
PA2519	*xylS*	transcriptional regulator XylS
PA2560		hypothetical protein
PA2570	*lecA*	LecA
PA2582		hypothetical protein
PA2662		conserved hypothetical protein
PA2663		hypothetical protein
PA2664	*fhp*	flavohemoprotein

PA2710		hypothetical protein
PA2718		probable transcriptional regulator
PA2743	*infC*	translation initiation factor IF-3
PA2746		hypothetical protein
PA2759		hypothetical protein
PA2786		hypothetical protein
PA2844		conserved hypothetical protein
PA2847		conserved hypothetical protein
PA2876	*pyrF*	orotidine 5'-phosphate decarboxylase
PA2906		probable oxidoreductase
PA2950		hypothetical protein
PA2985		hypothetical protein
PA3009		hypothetical protein
PA3010		hypothetical protein
PA3034		probable transcriptional regulator
PA3167	*serC*	3-phosphoserine aminotransferase
PA3168	*gyrA*	DNA gyrase subunit A
PA3178		hypothetical protein
PA3182	*pgl*	6-phosphogluconolactonase
PA3183	*zwf*	glucose-6-phosphate 1-dehydrogenase
PA3195	*gapA*	glyceraldehyde 3-phosphate dehydrogenase
PA3266	*capB*	cold acclimation protein B
PA3285		probable sigma-70 factor, ECF subfamily
PA3354		hypothetical protein
PA3417		probable pyruvate dehydrogenase E1 component, alpha subunit
PA3436		hypothetical protein
PA3450		probable antioxidant protein
PA3473		hypothetical protein
PA3533		conserved hypothetical protein
PA3536		hypothetical protein
PA3576		hypothetical protein
PA3578		conserved hypothetical protein
PA3668		conserved hypothetical protein
PA3720		hypothetical protein
PA3726		conserved hypothetical protein
PA3744	*rimM*	16S rRNA processing protein
PA3754		hypothetical protein
PA3756		hypothetical protein
PA3796		hypothetical protein
PA3808		conserved hypothetical protein
PA3813	*iscU*	probable iron-binding protein IscU
PA3817		probable methyltransferase
PA3844		hypothetical protein
PA3899		probable sigma-70 factor, ECF subfamily
PA3914	*moeA1*	molybdenum cofactor biosynthetic protein A1
PA3915	*moaB1*	molybdopterin biosynthetic protein B1
PA3918	*moaC*	molybdopterin biosynthetic protein C
PA4007	*proA*	gamma-glutamyl phosphate reductase
PA4014		hypothetical protein
PA4021		probable transcriptional regulator
PA4026		probable acetyltransferase
PA4029		conserved hypothetical protein
PA4034	*aqpZ*	aquaporin Z

PA4044	*dxs*	1-deoxyxylulose-5-phosphate synthase
PA4045		conserved hypothetical protein
PA4046		hypothetical protein
PA4051	*thiL*	thiamine monophosphate kinase
PA4069		hypothetical protein
PA4147	*acoR*	transcriptional regulator AcoR
PA4176	*ppiC2*	peptidyl-prolyl cis-trans isomerase C2
PA4245	*rpmD*	50S ribosomal protein L30
PA4248	*rplF*	50S ribosomal protein L6
PA4262	*rplD*	50S ribosomal protein L4
PA4263	*rplC*	50S ribosomal protein L3
PA4293	*pprA*	two-component sensor PprA
PA4326		hypothetical protein
PA4379		conserved hypothetical protein
PA4442	*cysN*	ATP sulfurylase GTP-binding subunit/APS kinase
PA4443	*cysD*	ATP sulfurylase small subunit
PA4544	*rluD*	pseudouridine synthase
PA4567	*rpmA*	50S ribosomal protein L27
PA4575		hypothetical protein
PA4602	*glyA3*	serine hydroxymethyltransferase
PA4630		hypothetical protein
PA4637		hypothetical protein
PA4666	*hemA*	glutamyl-tRNA reductase
PA4726	*cbrB*	two-component response regulator CbrB
PA4742	*truB*	tRNA pseudouridine 55 synthase
PA4748	*tpiA*	triosephosphate isomerase
PA4750	*folP*	dihydropteroate synthase
PA4758	*carA*	carbamoyl-phosphate synthase small chain
PA4780		conserved hypothetical protein
PA4852		conserved hypothetical protein
PA4859		probable permease of ABC transporter
PA4870		conserved hypothetical protein
PA4881		hypothetical protein
PA4933		hypothetical protein
PA4939		conserved hypothetical protein
PA4940		conserved hypothetical protein
PA5028		conserved hypothetical protein
PA5048		probable nuclease
PA5176		conserved hypothetical protein
PA5249		hypothetical protein
PA5287	*amtB*	ammonium transporter AmtB
PA5288	*glnK*	nitrogen regulatory protein P-II 2
PA5295		hypothetical protein
PA5298		xanthine phosphoribosyltransferase
PA5337	*rpoZ*	RNA polymerase omega subunit
PA5346	*sadB*	SadB
PA5380	*gbdR*	GbdR
PA5383		conserved hypothetical protein
PA5438		probable transcriptional regulator
PA5446		hypothetical protein
PA5460		hypothetical protein
PA5468		probable citrate transporter
PA5504		D-methionine ABC transporter membrane protein
