# Supplementary material for: In-Vivo Expression Profiling of Pseudomonas aeruginosa Infections Reveals Niche-Specific and Strain-Independent Transcriptional Programs
Source: PLoS One. 2011 Sep 12;6(9):e24235. doi: 10.1371/journal.pone.0024235 (PMC3171414; doi:10.1371/journal.pone.0024235)
Supplement: Table S4 — Gene signature of P. aeruginosa under in vivo conditions in mouse tumor infection. (PDF) [file pone.0024235.s004.pdf]

Table S4

Locus ID	Gene name	Product name
PA0044	*exoT*	exoenzyme T
PA0046		hypothetical protein
PA0067	*prlC*	oligopeptidase A
PA0093		hypothetical protein
PA0132		beta-alanine--pyruvate transaminase
PA0141		conserved hypothetical protein
PA0396	*pilU*	twitching motility protein PilU
PA0415	*chpC*	probable chemotaxis protein
PA0442		hypothetical protein
PA0510		probable uroporphyrin-III c-methyltransferase
PA0517	*nirC*	probable c-type cytochrome precursor
PA0518	*nirM*	cytochrome c-551 precursor
PA0519	*nirS*	nitrite reductase precursor
PA0534		conserved hypothetical protein
PA0545		hypothetical protein
PA0572		hypothetical protein
PA0713		hypothetical protein
PA0715		hypothetical protein
PA0716		hypothetical protein
PA0781		hypothetical protein
PA0822		hypothetical protein
PA0835	*pta*	phosphate acetyltransferase
PA0836	*ackA*	acetate kinase
PA0864		probable transcriptional regulator
PA0874		hypothetical protein
PA0952		hypothetical protein
PA0962		probable dna-binding stress protein
PA0977		hypothetical protein
PA0985		pyocin S5
PA1029		hypothetical protein
PA1126		hypothetical protein
PA1127		probable oxidoreductase
PA1131		probable major facilitator superfamily (MFS) transporter
PA1195		hypothetical protein
PA1329		conserved hypothetical protein
PA1337	*ansB*	glutaminase-asparaginase
PA1369		hypothetical protein
PA1370		hypothetical protein
PA1371		hypothetical protein
PA1372		hypothetical protein
PA1388		hypothetical protein
PA1429		probable cation-transporting P-type ATPase
PA1555		probable cytochrome c
PA1557		probable cytochrome oxidase subunit (cbb3-type)
PA1664		hypothetical protein
PA1673		hypothetical protein
PA1692		probable translocation protein in type III secretion
PA1696	*pscO*	translocation protein in type III secretion
PA1699		conserved hypothetical protein in type III secretion
PA1700		conserved hypothetical protein in type III secretion
PA1701		conserved hypothetical protein in type III secretion
PA1706	*pcrV*	type III secretion protein PcrV

PA1707	*pcrH*	regulatory protein PcrH
PA1708	*popB*	translocator protein PopB
PA1709	*popD*	Translocator outer membrane protein PopD precursor
PA1711	*exsE*	ExsE
PA1714	*exsD*	ExsD
PA1715	*pscB*	type III export apparatus protein
PA1716	*pscC*	Type III secretion outer membrane protein PscC precursor
PA1717	*pscD*	type III export protein PscD
PA1718	*pscE*	type III export protein PscE
PA1719	*pscF*	type III export protein PscF
PA1720	*pscG*	type III export protein PscG
PA1721	*pscH*	type III export protein PscH
PA1722	*pscI*	type III export protein PscI
PA1723	*pscJ*	type III export protein PscJ
PA1724	*pscK*	type III export protein PscK
PA1734		hypothetical protein
PA1746		hypothetical protein
PA1789		hypothetical protein
PA1869		probable acyl carrier protein
PA1901	*phzC2*	phenazine biosynthesis protein PhzC
PA1904	*phzF2*	probable phenazine biosynthesis protein
PA1934		hypothetical protein
PA2037		hypothetical protein
PA2102		hypothetical protein
PA2119		alcohol dehydrogenase (Zn-dependent)
PA2127		conserved hypothetical protein
PA2128	*cupA1*	fimbrial subunit CupA1
PA2186		hypothetical protein
PA2191	*exoY*	adenylate cyclase ExoY
PA2392	*pvdP*	PvdP
PA2436		hypothetical protein
PA2460		hypothetical protein
PA2461		hypothetical protein
PA2576		hypothetical protein
PA2730		hypothetical protein
PA2733		conserved hypothetical protein
PA2734		hypothetical protein
PA2753		hypothetical protein
PA2819		hypothetical protein
PA2825		probable transcriptional regulator
PA2826		probable glutathione peroxidase
PA2931	*cifR*	CifR
PA3115	*fimV*	Motility protein FimV
PA3140		hypothetical protein
PA3144		hypothetical protein
PA3147	*wbpJ*	probable glycosyl transferase WbpJ
PA3148	*wbpI*	probable UDP-N-acetylglucosamine 2-epimerase WbpI
PA3149	*wbpH*	probable glycosyltransferase WbpH
PA3150	*wbpG*	LPS biosynthesis protein WbpG
PA3151	*hisF2*	imidazoleglycerol-phosphate synthase, cyclase subunit
PA3152	*hisH2*	glutamine amidotransferase
PA3154	*wzy*	B-band O-antigen polymerase
PA3155	*wbpE*	probable aminotransferase WbpE
PA3156	*wbpD*	probable acetyltransferase WbpD
PA3157		probable acetyltransferase

PA3159	*wbpA*	probable UDP-glucose/GDP-mannose dehydrogenase WbpA
PA3160	*wzz*	O-antigen chain length regulator
PA3161	*himD*	integration host factor beta subunit
PA3278		hypothetical protein
PA3291		hypothetical protein
PA3309		conserved hypothetical protein
PA3337	*rfaD*	ADP-L-glycero-D-mannoheptose 6-epimerase
PA3431		conserved hypothetical protein
PA3432		hypothetical protein
PA3458		probable transcriptional regulator
PA3520		hypothetical protein
PA3529		probable peroxidase
PA3572		hypothetical protein
PA3613		hypothetical protein
PA3615		hypothetical protein
PA3841	*exoS*	exoenzyme S
PA3842		probable chaperone
PA3866		pyocin protein
PA3880		conserved hypothetical protein
PA3968		probable pseudouridine synthase
PA4028		hypothetical protein
PA4067	*oprG*	Outer membrane protein OprG precursor
PA4141		hypothetical protein
PA4211	*phzB1*	probable phenazine biosynthesis protein
PA4217	*phzS*	flavin-containing monooxygenase
PA4348		conserved hypothetical protein
PA4352		conserved hypothetical protein
PA4385	*groEL*	GroEL protein
PA4386	*groES*	GroES protein
PA4407	*ftsZ*	cell division protein FtsZ
PA4463		conserved hypothetical protein
PA4577		hypothetical protein
PA4578		hypothetical protein
PA4587	*ccpR*	cytochrome c551 peroxidase precursor
PA4610		hypothetical protein
PA4699		hypothetical protein
PA4760	*dnaJ*	DnaJ protein
PA4922	*azu*	azurin precursor
PA5027		hypothetical protein
PA5044	*pilM*	type 4 fimbrial biogenesis protein PilM
PA5086		hypothetical protein
PA5087		hypothetical protein
PA5105	*hutC*	histidine utilization repressor HutC
PA5106		conserved hypothetical protein
PA5170	*arcD*	arginine/ornithine antiporter
PA5171	*arcA*	arginine deiminase
PA5172	*arcB*	ornithine carbamoyltransferase, catabolic
PA5173	*arcC*	carbamate kinase
PA5232		conserved hypothetical protein
PA5427	*adhA*	alcohol dehydrogenase
PA5475		hypothetical protein
PA5494		hypothetical protein
