# Supplementary material for: In-Vivo Expression Profiling of Pseudomonas aeruginosa Infections Reveals Niche-Specific and Strain-Independent Transcriptional Programs
Source: PLoS One. 2011 Sep 12;6(9):e24235. doi: 10.1371/journal.pone.0024235 (PMC3171414; doi:10.1371/journal.pone.0024235)
Supplement: Table S5 — Gene signature of P. aeruginosa under in vitro conditions in biofilm growth. (PDF) [file pone.0024235.s005.pdf]

Table S5

Locus ID	Gene name	Product name
PA0089		hypothetical protein
PA0128		conserved hypothetical protein
PA0140	*ahpF*	alkyl hydroperoxide reductase subunit F
PA0161		hypothetical protein
PA0172		hypothetical protein
PA0257		hypothetical protein
PA0261		hypothetical protein
PA0263	*hcpC*	secreted protein Hcp
PA0291	*oprE*	Anaerobically-induced outer membrane porin OprE precursor
PA0297	*spuA*	probable glutamine amidotransferase
PA0359		hypothetical protein
PA0363	*coaD*	phosphopantetheine adenylyltransferase
PA0381	*thiG*	thiamine biosynthesis protein, thiazole moiety
PA0408	*pilG*	twitching motility protein PilG
PA0409	*pilH*	twitching motility protein PilH
PA0410	*pilI*	twitching motility protein PilI
PA0411	*pilJ*	twitching motility protein PilJ
PA0424	*mexR*	multidrug resistance operon repressor MexR
PA0506		probable acyl-CoA dehydrogenase
PA0541		hypothetical protein
PA0563		conserved hypothetical protein
PA0576	*rpoD*	sigma factor RpoD
PA0578		conserved hypothetical protein
PA0579	*rpsU*	30S ribosomal protein S21
PA0581		conserved hypothetical protein
PA0589		conserved hypothetical protein
PA0595	*ostA*	organic solvent tolerance protein OstA precursor
PA0652	*vfr*	transcriptional regulator Vfr
PA0654	*speD*	S-adenosylmethionine decarboxylase proenzyme
PA0655		hypothetical protein
PA0667		conserved hypothetical protein
PA0767	*lepA*	GTP-binding protein LepA
PA0768	*lepB*	signal peptidase I
PA0805		hypothetical protein
PA0915		conserved hypothetical protein
PA0916		conserved hypothetical protein
PA0944	*purN*	phosphoribosylaminoimidazole synthetase
PA0945	*purM*	phosphoribosylaminoimidazole synthetase
PA0955		hypothetical protein
PA0961		probable cold-shock protein
PA0965	*ruvC*	Holliday junction resolvase RuvC
PA0974		conserved hypothetical protein
PA0981		hypothetical protein
PA0996	*pqsA*	probable coenzyme A ligase
PA0998	*pqsC*	Homologous to beta-keto-acyl-acyl-carrier protein synthase
PA1006		conserved hypothetical protein
PA1034		hypothetical protein
PA1080	*flgE*	flagellar hook protein FlgE
PA1094	*fliD*	flagellar capping protein FliD
PA1123		hypothetical protein
PA1132		hypothetical protein
PA1159		probable cold-shock protein

PA1168		hypothetical protein
PA1183	*dctA*	C4-dicarboxylate transport protein
PA1193		hypothetical protein
PA1439		conserved hypothetical protein
PA1477	*ccmC*	heme exporter protein CcmC
PA1479	*ccmE*	cytochrome C-type biogenesis protein CcmE
PA1480	*ccmF*	cytochrome C-type biogenesis protein CcmF
PA1482	*ccmH*	cytochrome C-type biogenesis protein CcmH
PA1541		probable drug efflux transporter
PA1543	*apt*	adenine phosphoribosyltransferase
PA1546	*hemN*	oxygen-independent coproporphyrinogen III oxidase
PA1551		probable ferredoxin
PA1556		probable cytochrome c oxidase subunit
PA1564		conserved hypothetical protein
PA1580	*gltA*	citrate synthase
PA1582	*sdhD*	succinate dehydrogenase (D subunit)
PA1584	*sdhB*	succinate dehydrogenase (B subunit)
PA1610	*fabA*	beta-hydroxydecanoyl-ACP dehydrase
PA1656		hypothetical protein
PA1657		conserved hypothetical protein
PA1658		conserved hypothetical protein
PA1659		hypothetical protein
PA1660		hypothetical protein
PA1661		hypothetical protein
PA1668		hypothetical protein
PA1669		hypothetical protein
PA1713	*exsA*	transcriptional regulator ExsA
PA1767		hypothetical protein
PA1768		hypothetical protein
PA1774	*cfrX*	CfrX protein
PA1801	*clpP*	ATP-dependent Clp protease proteolytic subunit
PA1805	*ppiD*	peptidyl-prolyl cis-trans isomerase D
PA1812	*mltD*	membrane-bound lytic murein transglycosylase D precursor
PA1830		hypothetical protein
PA2042		probable transporter (membrane subunit)
PA2112		conserved hypothetical protein
PA2456		hypothetical protein
PA2459		hypothetical protein
PA2584	*pgsA*	CDP-diacylglycerol--glycerol-3-phosphate 3-phosphatidyltransferase
PA2593		hypothetical protein
PA2619	*infA*	initiation factor
PA2624	*idh*	isocitrate dehydrogenase
PA2627		conserved hypothetical protein
PA2629	*purB*	adenylosuccinate lyase
PA2637	*nuoA*	NADH dehydrogenase I chain A
PA2639	*nuoD*	NADH dehydrogenase I chain C,D
PA2666		probable 6-pyruvoyl tetrahydrobiopterin synthase
PA2667		conserved hypothetical protein
PA2741	*rplT*	50S ribosomal protein L20
PA2769		hypothetical protein
PA2840		probable ATP-dependent RNA helicase
PA2851	*efp*	translation elongation factor P
PA2953		electron transfer flavoprotein-ubiquinone oxidoreductase
PA2957		probable transcriptional regulator
PA2959		conserved hypothetical protein

PA2960	*pilZ*	type 4 fimbrial biogenesis protein PilZ
PA2966	*acpP*	acyl carrier protein
PA2970	*rpmF*	50S ribosomal protein L32
PA2971		conserved hypothetical protein
PA2986		conserved hypothetical protein
PA3013	*foaB*	fatty-acid oxidation complex beta-subunit
PA3057		hypothetical protein
PA3112	*accD*	acetyl-CoA carboxylase beta subunit
PA3162	*rpsA*	30S ribosomal protein S1
PA3217	*cyaB*	CyaB
PA3245	*minE*	cell division topological specificity factor MinE
PA3280	*oprO*	Pyrophosphate-specific outer membrane porin OprO precursor
PA3292		hypothetical protein
PA3299	*fadD1*	long-chain-fatty-acid--CoA ligase
PA3332		conserved hypothetical protein
PA3383		binding protein component of ABC phosphonate transporter
PA3480		probable deoxycytidine triphosphate deaminase
PA3489		conserved hypothetical protein
PA3525	*argG*	argininosuccinate synthase
PA3531	*bfrB*	bacterioferritin
PA3611		hypothetical protein
PA3612		conserved hypothetical protein
PA3621	*fdxA*	ferredoxin I
PA3633	*ygbP*	4-diphosphocytidyl-2-C-methylerythritol synthase
PA3636	*kdsA*	2-dehydro-3-deoxyphosphooctonate aldolase
PA3639	*accA*	acetyl-coenzyme A carboxylase carboxyl transferase (alpha subunit)
PA3642	*rnhB*	ribonuclease HII
PA3644	*lpxA*	UDP-N-acetylglucosamine acyltransferase
PA3645	*fabZ*	(3R)-hydroxymyristoyl-[acyl carrier protein] dehydratase
PA3646	*lpxD*	UDP-3-O-[3-hydroxylauroyl] glucosamine N-acyltransferase
PA3648	*opr86*	outer membrane protein Opr86
PA3653	*frr*	ribosome recycling factor
PA3654	*pyrH*	uridylate kinase
PA3655	*tsf*	elongation factor Ts
PA3656	*rpsB*	30S ribosomal protein S2
PA3686	*adk*	adenylate kinase
PA3700	*lysS*	lysyl-tRNA synthetase
PA3722		hypothetical protein
PA3741		hypothetical protein
PA3742	*rplS*	50S ribosomal protein L19
PA3743	*trmD*	tRNA (guanine-N1)-methyltransferase
PA3745	*rpsP*	30S ribosomal protein S16
PA3770	*guaB*	inosine-5'-monophosphate dehydrogenase
PA3804		hypothetical protein
PA3805	*pilF*	type 4 fimbrial biogenesis protein PilF
PA3806		conserved hypothetical protein
PA3818		extragenic suppressor protein SuhB
PA3822		conserved hypothetical protein
PA3824	*queA*	S-adenosylmethionine:trna ribosyltransferase-isomerase
PA3903	*prfC*	peptide chain release factor 3
PA3905		hypothetical protein
PA3906		hypothetical protein
PA3907		hypothetical protein
PA3908		hypothetical protein
PA3941		hypothetical protein

PA3966		hypothetical protein
PA3967		hypothetical protein
PA3979		hypothetical protein
PA3980		conserved hypothetical protein
PA3982		conserved hypothetical protein
PA3989	*holA*	DNA polymerase III, delta subunit
PA4031	*ppa*	inorganic pyrophosphatase
PA4130		probable sulfite or nitrite reductase
PA4131		probable iron-sulfur protein
PA4132		conserved hypothetical protein
PA4133		cytochrome c oxidase subunit (cbb3-type)
PA4134		hypothetical protein
PA4139		hypothetical protein
PA4140		hypothetical protein
PA4142		probable secretion protein
PA4237	*rplQ*	50S ribosomal protein L17
PA4238	*rpoA*	DNA-directed RNA polymerase alpha chain
PA4239	*rpsD*	30S ribosomal protein S4
PA4240	*rpsK*	30S ribosomal protein S11
PA4241	*rpsM*	30S ribosomal protein S13
PA4242	*rpmJ*	50S ribosomal protein L36
PA4243	*secY*	secretion protein SecY
PA4246	*rpsE*	30S ribosomal protein S5
PA4247	*rplR*	50S ribosomal protein L18
PA4249	*rpsH*	30S ribosomal protein S8
PA4250	*rpsN*	30S ribosomal protein S14
PA4251	*rplE*	50S ribosomal protein L5
PA4252	*rplX*	50S ribosomal protein L24
PA4253	*rplN*	50S ribosomal protein L14
PA4254	*rpsQ*	30S ribosomal protein S17
PA4258	*rplV*	50S ribosomal protein L22
PA4261	*rplW*	50S ribosomal protein L23
PA4264	*rpsJ*	30S ribosomal protein S10
PA4265	*tufA*	elongation factor Tu
PA4266	*fusA1*	elongation factor G
PA4268	*rpsL*	30S ribosomal protein S12
PA4269	*rpoC*	DNA-directed RNA polymerase beta* chain
PA4270	*rpoB*	DNA-directed RNA polymerase beta chain
PA4271	*rplL*	50S ribosomal protein L7 / L12
PA4272	*rplJ*	50S ribosomal protein L10
PA4273	*rplA*	50S ribosomal protein L1
PA4274	*rplK*	50S ribosomal protein L11
PA4275	*nusG*	transcription antitermination protein NusG
PA4276	*secE*	secretion protein SecE
PA4317		hypothetical protein
PA4318		hypothetical protein
PA4319		conserved hypothetical protein
PA4405		hypothetical protein
PA4406	*lpxC*	UDP-3-O-acyl-N-acetylglucosamine deacetylase
PA4426		conserved hypothetical protein
PA4427	*sspB*	stringent starvation protein B
PA4428	*sspA*	stringent starvation protein A
PA4429		probable cytochrome c1 precursor
PA4430		probable cytochrome b
PA4431		probable iron-sulfur protein

PA4432	*rpsI*	30S ribosomal protein S9
PA4433	*rplM*	50S ribosomal protein L13
PA4449	*hisG*	ATP-phosphoribosyltransferase
PA4451		conserved hypothetical protein
PA4458		conserved hypothetical protein
PA4459		conserved hypothetical protein
PA4460		conserved hypothetical protein
PA4461		probable ATP-binding component of ABC transporter
PA4465		conserved hypothetical protein
PA4466		probable phosphoryl carrier protein
PA4479	*mreD*	rod shape-determining protein MreD
PA4480	*mreC*	rod shape-determining protein MreC
PA4482	*gatC*	Glu-tRNA(Gln) amidotransferase subunit C
PA4494		probable two-component sensor
PA4528	*pilD*	type 4 prepilin peptidase PilD
PA4530		conserved hypothetical protein
PA4554	*pilY1*	type 4 fimbrial biogenesis protein PilY1
PA4563	*rpsT*	30S ribosomal protein S20
PA4568	*rplU*	50S ribosomal protein L21
PA4569	*ispB*	octaprenyl-diphosphate synthase
PA4574		conserved hypothetical protein
PA4625		hypothetical protein
PA4640	*mqoB*	malate:quinone oxidoreductase
PA4670	*prs*	ribose-phosphate pyrophosphokinase
PA4671		probable ribosomal protein L25
PA4672		peptidyl-tRNA hydrolase
PA4673		conserved hypothetical protein
PA4678	*rimI*	peptide n-acetyltransferase RimI
PA4728	*folK*	2-amino-4-hydroxy-6-hydroxymethyldihydropteridine pyrophosphokinase
PA4740	*pnp*	polyribonucleotide nucleotidyltransferase
PA4743	*rbfA*	ribosome-binding factor A
PA4744	*infB*	translation initiation factor IF-2
PA4745	*nusA*	N utilization substance protein A
PA4746		conserved hypothetical protein
PA4747	*secG*	secretion protein SecG
PA4753		conserved hypothetical protein
PA4757		conserved hypothetical protein
PA4765	*omlA*	Outer membrane lipoprotein OmlA precursor
PA4768	*smpB*	SmpB protein
PA4846	*aroQ1*	3-dehydroquinate dehydratase
PA4853	*fis*	DNA-binding protein Fis
PA4932	*rplI*	50S ribosomal protein L9
PA4934	*rpsR*	30S ribosomal protein S18
PA4935	*rpsF*	30S ribosomal protein S6
PA4962		conserved hypothetical protein
PA4965		hypothetical protein
PA4997	*msbA*	transport protein MsbA
PA5033		hypothetical protein
PA5043	*pilN*	type 4 fimbrial biogenesis protein PilN
PA5045	*ponA*	penicillin-binding protein 1A
PA5046		malic enzyme
PA5049	*rpmE*	50S ribosomal protein L31
PA5117	*typA*	regulatory protein TypA
PA5118	*thiI*	thiazole biosynthesis protein ThiI
PA5130		conserved hypothetical protein

PA5139		hypothetical protein
PA5203	*gshA*	glutamate--cysteine ligase
PA5239	*rho*	transcription termination factor Rho
PA5244		conserved hypothetical protein
PA5296	*rep*	ATP-dependent DNA helicase Rep
PA5300	*cycB*	cytochrome c5
PA5302	*dadX*	catabolic alanine racemase
PA5315	*rpmG*	50S ribosomal protein L33
PA5316	*rpmB*	50S ribosomal protein L28
PA5338	*spoT*	guanosine-3',5'-bis(diphosphate) 3'-pyrophosphohydrolase
PA5351	*rubA1*	Rubredoxin 1
PA5366	*pstB*	ATP-binding component of ABC phosphate transporter
PA5367	*pstA*	membrane protein component of ABC phosphate transporter
PA5369	*pstS*	phosphate ABC transporter, periplasmic phosphate-binding protein, PstS
PA5435		probable transcarboxylase subunit
PA5462		hypothetical protein
PA5479	*gltP*	proton-glutamate symporter
PA5490	*cc4*	cytochrome c4 precursor
PA5491		probable cytochrome
PA5492		conserved hypothetical protein
PA5505		probable TonB-dependent receptor
PA5554	*atpD*	ATP synthase beta chain
PA5555	*atpG*	ATP synthase gamma chain
PA5556	*atpA*	ATP synthase alpha chain
PA5557	*atpH*	ATP synthase delta chain
PA5560	*atpB*	ATP synthase A chain
PA5561	*atpI*	ATP synthase protein I
PA5563	*soj*	chromosome partitioning protein Soj
PA5568		conserved hypothetical protein
PA5569	*rnpA*	ribonuclease P protein component
PA5570	*rpmH*	50S ribosomal protein L34
