# Supplementary material for: In-Vivo Expression Profiling of Pseudomonas aeruginosa Infections Reveals Niche-Specific and Strain-Independent Transcriptional Programs
Source: PLoS One. 2011 Sep 12;6(9):e24235. doi: 10.1371/journal.pone.0024235 (PMC3171414; doi:10.1371/journal.pone.0024235)
Supplement: Table S6 — Gene signature of P. aeruginosa under in vitro conditions in planktonic growth. (PDF) [file pone.0024235.s006.pdf]

Table S6

Locus ID	Gene name	Product name
PA0038		hypothetical protein
PA0039		hypothetical protein
PA0052		hypothetical protein
PA0105	*coxB*	cytochrome c oxidase, subunit II
PA0106	*coxA*	cytochrome c oxidase, subunit I
PA0107		conserved hypothetical protein
PA0108	*coIII*	cytochrome c oxidase, subunit III
PA0109		hypothetical protein
PA0110		hypothetical protein
PA0111		hypothetical protein
PA0112		hypothetical protein
PA0113		probable cytochrome c oxidase assembly factor
PA0122		conserved hypothetical protein
PA0156	*triA*	Resistance-Nodulation-Cell Division (RND) triclosan efflux membrane fusion protein, TriA
PA0157	*triB*	Resistance-Nodulation-Cell Division (RND) triclosan efflux membrane fusion protein, TriB
PA0173		probable methylesterase
PA0175		probable chemotaxis protein methyltransferase
PA0176	*aer2*	aerotaxis transducer Aer2
PA0177		probable purine-binding chemotaxis protein
PA0178		probable two-component sensor
PA0179		probable two-component response regulator
PA0180		probable chemotaxis transducer
PA0200		hypothetical protein
PA0249		probable acetyltransferase
PA0250		conserved hypothetical protein
PA0256		hypothetical protein
PA0312		conserved hypothetical protein
PA0329		conserved hypothetical protein
PA0332		hypothetical protein
PA0365		hypothetical protein
PA0366		probable aldehyde dehydrogenase
PA0384		hypothetical protein
PA0387		conserved hypothetical protein
PA0388		hypothetical protein
PA0432	*sahH*	S-adenosyl-L-homocysteine hydrolase
PA0452		probable stomatin-like protein
PA0459		probable ClpA/B protease ATP binding subunit
PA0484		conserved hypothetical protein
PA0520	*nirQ*	regulatory protein NirQ
PA0540		hypothetical protein
PA0586		conserved hypothetical protein
PA0588		conserved hypothetical protein
PA0612	*ptrB*	repressor, PtrB
PA0614		hypothetical protein
PA0616		hypothetical protein
PA0619		probable bacteriophage protein
PA0624		hypothetical protein
PA0631		hypothetical protein
PA0635		hypothetical protein
PA0636		hypothetical protein

PA0648		hypothetical protein
PA0656		probable HIT family protein
PA0745		probable enoyl-CoA hydratase/isomerase
PA0746		probable acyl-CoA dehydrogenase
PA0762	*algU*	sigma factor AlgU
PA0776		hypothetical protein
PA0865	*hpd*	4-hydroxyphenylpyruvate dioxygenase
PA0866	*aroP2*	aromatic amino acid transport protein AroP2
PA0870	*phhC*	aromatic amino acid aminotransferase
PA0871	*phhB*	pterin-4-alpha-carbinolamine dehydratase
PA0872	*phhA*	phenylalanine-4-hydroxylase
PA0887	*acsA*	acetyl-coenzyme A synthetase
PA0921		hypothetical protein
PA0959		hypothetical protein
PA0960		hypothetical protein
PA1002	*phnB*	anthranilate synthase component II
PA1027		probable aldehyde dehydrogenase
PA1041		probable outer membrane protein precursor
PA1042		conserved hypothetical protein
PA1048		probable outer membrane protein precursor
PA1053		conserved hypothetical protein
PA1084	*flgI*	flagellar P-ring protein precursor FlgI
PA1121		conserved hypothetical protein
PA1172	*napC*	cytochrome c-type protein NapC
PA1173	*napB*	cytochrome c-type protein NapB precursor
PA1174	*napA*	periplasmic nitrate reductase protein NapA
PA1175	*napD*	NapD protein of periplasmic nitrate reductase
PA1176	*napF*	ferredoxin protein NapF
PA1177	*napE*	periplasmic nitrate reductase protein NapE
PA1178	*oprH*	PhoP/Q and low Mg2+ inducible outer membrane protein H1 precursor
PA1179	*phoP*	two-component response regulator PhoP
PA1180	*phoQ*	two-component sensor PhoQ
PA1202		probable hydrolase
PA1246	*aprD*	alkaline protease secretion protein AprD
PA1247	*aprE*	alkaline protease secretion protein AprE
PA1249	*aprA*	alkaline metalloproteinase precursor
PA1283		probable transcriptional regulator
PA1287		probable glutathione peroxidase
PA1289		hypothetical protein
PA1301		probable transmembrane sensor
PA1327		probable protease
PA1342		probable binding protein component of ABC transporter
PA1348		hypothetical protein
PA1353		hypothetical protein
PA1430	*lasR*	transcriptional regulator LasR
PA1431	*rsaL*	regulatory protein RsaL
PA1432	*lasI*	autoinducer synthesis protein LasI
PA1454	*fleN*	flagellar synthesis regulator FleN
PA1455	*fliA*	sigma factor FliA
PA1456	*cheY*	two-component response regulator CheY
PA1458		probable two-component sensor
PA1462		probable plasmid partitioning protein
PA1464		probable purine-binding chemotaxis protein
PA1545		hypothetical protein
PA1617		probable AMP-binding enzyme

PA1641		hypothetical protein
PA1728		hypothetical protein
PA1729		conserved hypothetical protein
PA1733		conserved hypothetical protein
PA1752		hypothetical protein
PA1753		conserved hypothetical protein
PA1760		probable transcriptional regulator
PA1762		hypothetical protein
PA1784		hypothetical protein
PA1817		hypothetical protein
PA1819		probable amino acid permease
PA1860		hypothetical protein
PA1874		hypothetical protein
PA1875		probable outer membrane protein precursor
PA1876		probable ATP-binding/permease fusion ABC transporter
PA1887		hypothetical protein
PA1888		hypothetical protein
PA1914		conserved hypothetical protein
PA1931		probable ferredoxin
PA1944		hypothetical protein
PA1985	*pqqA*	pyrroloquinoline quinone biosynthesis protein A
PA2006		probable major facilitator superfamily (MFS) transporter
PA2007	*maiA*	maleylacetoacetate isomerase
PA2009	*hmgA*	homogentisate 1,2-dioxygenase
PA2020		probable transcriptional regulator
PA2024		probable ring-cleaving dioxygenase
PA2025	*gor*	glutathione reductase
PA2027		hypothetical protein
PA2166		hypothetical protein
PA2174		hypothetical protein
PA2237	*pslG*	probable glycosyl hydrolase
PA2247	*bkdA1*	2-oxoisovalerate dehydrogenase (alpha subunit)
PA2248	*bkdA2*	2-oxoisovalerate dehydrogenase (beta subunit)
PA2249	*bkdB*	branched-chain alpha-keto acid dehydrogenase (lipoamide component)
PA2250	*lpdV*	lipoamide dehydrogenase-Val
PA2364		hypothetical protein
PA2365		conserved hypothetical protein
PA2367		hypothetical protein
PA2375		hypothetical protein
PA2381		hypothetical protein
PA2422		hypothetical protein
PA2423		hypothetical protein
PA2433		hypothetical protein
PA2434		hypothetical protein
PA2501		hypothetical protein
PA2504		hypothetical protein
PA2511		probable transcriptional regulator
PA2562		hypothetical protein
PA2564		hypothetical protein
PA2565		hypothetical protein
PA2566		conserved hypothetical protein
PA2571		probable two-component sensor
PA2573		probable chemotaxis transducer
PA2577		probable transcriptional regulator
PA2587	*pqsH*	probable FAD-dependent monooxygenase

PA2591		probable transcriptional regulator
PA2605		conserved hypothetical protein
PA2607		conserved hypothetical protein
PA2618		hypothetical protein
PA2620	*clpA*	ATP-binding protease component ClpA
PA2622	*cspD*	cold-shock protein CspD
PA2640	*nuoE*	NADH dehydrogenase I chain E
PA2643	*nuoH*	NADH dehydrogenase I chain H
PA2645	*nuoJ*	NADH dehydrogenase I chain J
PA2646	*nuoK*	NADH dehydrogenase I chain K
PA2747		hypothetical protein
PA2754		conserved hypothetical protein
PA2762		hypothetical protein
PA2771		conserved hypothetical protein
PA2779		hypothetical protein
PA2799		hypothetical protein
PA2827		conserved hypothetical protein
PA2841		probable enoyl-CoA hydratase/isomerase
PA2849		probable transcriptional regulator
PA2883		hypothetical protein
PA2897		probable transcriptional regulator
PA2915		hypothetical protein
PA2920		probable chemotaxis transducer
PA2937		hypothetical protein
PA2939		probable aminopeptidase
PA3017		conserved hypothetical protein
PA3032	*snr1*	cytochrome c Snr1
PA3040		conserved hypothetical protein
PA3068	*gdhB*	NAD-dependent glutamate dehydrogenase
PA3186	*oprB*	Glucose/carbohydrate outer membrane porin OprB precursor
PA3187		probable ATP-binding component of ABC transporter
PA3188		probable permease of ABC sugar transporter
PA3216		hypothetical protein
PA3229		hypothetical protein
PA3234		probable sodium:solute symporter
PA3235		conserved hypothetical protein
PA3311		conserved hypothetical protein
PA3316		probable permease of ABC transporter
PA3343		hypothetical protein
PA3345		hypothetical protein
PA3346		probable two-component response regulator
PA3347		hypothetical protein
PA3348		probable chemotaxis protein methyltransferase
PA3349		probable chemotaxis protein
PA3351	*flgM*	FlgM
PA3352		hypothetical protein
PA3369		hypothetical protein
PA3371		hypothetical protein
PA3415		probable dihydrolipoamide acetyltransferase
PA3416		probable pyruvate dehydrogenase E1 component, beta chain
PA3418	*ldh*	leucine dehydrogenase
PA3451		hypothetical protein
PA3465		conserved hypothetical protein
PA3477	*rhlR*	transcriptional regulator RhlR
PA3496		hypothetical protein

PA3526		probable outer membrane protein precursor
PA3568		probable acetyl-coa synthetase
PA3569	*mmsB*	3-hydroxyisobutyrate dehydrogenase
PA3570	*mmsA*	methylmalonate-semialdehyde dehydrogenase
PA3622	*rpoS*	sigma factor RpoS
PA3628		probable esterase
PA3629	*adhC*	alcohol dehydrogenase class III
PA3662		hypothetical protein
PA3674		hypothetical protein
PA3684		hypothetical protein
PA3688		hypothetical protein
PA3723		probable FMN oxidoreductase
PA3724	*lasB*	elastase LasB
PA3740		hypothetical protein
PA3753		conserved hypothetical protein
PA3784		hypothetical protein
PA3785		conserved hypothetical protein
PA3786		hypothetical protein
PA3792	*leuA*	2-isopropylmalate synthase
PA3833		hypothetical protein
PA3846		hypothetical protein
PA3847		conserved hypothetical protein
PA3848		hypothetical protein
PA3851		hypothetical protein
PA3858		probable amino acid-binding protein
PA3922		conserved hypothetical protein
PA3923		hypothetical protein
PA3945		conserved hypothetical protein
PA3957		probable short-chain dehydrogenase
PA3986		hypothetical protein
PA4012		hypothetical protein
PA4079		probable dehydrogenase
PA4112		probable sensor/response regulator hybrid
PA4129		hypothetical protein
PA4218		probable transporter
PA4219		hypothetical protein
PA4220		hypothetical protein
PA4221	*fptA*	Fe(III)-pyochelin outer membrane receptor precursor
PA4222		probable ATP-binding component of ABC transporter
PA4223		probable ATP-binding component of ABC transporter
PA4224	*pchG*	pyochelin biosynthetic protein PchG
PA4225	*pchF*	pyochelin synthetase
PA4226	*pchE*	dihydroaeruginoic acid synthetase
PA4227	*pchR*	transcriptional regulator PchR
PA4228	*pchD*	pyochelin biosynthesis protein PchD
PA4229	*pchC*	pyochelin biosynthetic protein PchC
PA4230	*pchB*	salicylate biosynthesis protein PchB
PA4231	*pchA*	salicylate biosynthesis isochorismate synthase
PA4290		probable chemotaxis transducer
PA4294		hypothetical protein
PA4296	*pprB*	two-component response regulator, PprB
PA4297	*tadG*	TadG
PA4298		hypothetical protein
PA4299	*tadD*	TadD
PA4300	*tadC*	TadC

PA4301	*tadB*	TadB
PA4302	*tadA*	TadA ATPase
PA4303	*tadZ*	TadZ
PA4304	*rcpA*	RcpA
PA4305	*rcpC*	RcpC
PA4306	*flp*	Type IVb pilin, Flp
PA4311		conserved hypothetical protein
PA4315	*mvaT*	transcriptional regulator MvaT, P16 subunit
PA4324		hypothetical protein
PA4377		hypothetical protein
PA4410	*ddlB*	D-alanine--D-alanine ligase
PA4411	*murC*	UDP-N-acetylmuramate--alanine ligase
PA4412	*murG*	UDP-N-acetylglucosamine--N-acetylmuramyl-(pentapeptide) pyrophosphoryl-undecaprenol N-acetylglucosamine transferase
PA4414	*murD*	UDP-N-acetylmuramoylalanine--D-glutamate ligase
PA4415	*mraY*	phospho-N-acetylmuramoyl-pentapeptide-transferase
PA4417	*murE*	UDP-N-acetylmuramoylalanyl-D-glutamate-2, 6-diaminopimelate ligase
PA4418	*ftsI*	penicillin-binding protein 3
PA4420		conserved hypothetical protein
PA4474		conserved hypothetical protein
PA4507		hypothetical protein
PA4572	*fklB*	peptidyl-prolyl cis-trans isomerase FklB
PA4573		hypothetical protein
PA4590	*pra*	protein activator
PA4607		hypothetical protein
PA4608		hypothetical protein
PA4611		hypothetical protein
PA4633		probable chemotaxis transducer
PA4641		still frameshift hypothetical protein
PA4648		hypothetical protein
PA4651		probable pili assembly chaperone
PA4702		hypothetical protein
PA4703		hypothetical protein
PA4713		hypothetical protein
PA4717		conserved hypothetical protein
PA4733	*acsB*	acetyl-coenzyme A synthetase
PA4735		hypothetical protein
PA4736		hypothetical protein
PA4766		conserved hypothetical protein
PA4767		conserved hypothetical protein
PA4778		probable transcriptional regulator
PA4781		probable two-component response regulator
PA4782		hypothetical protein
PA4793		hypothetical protein
PA4810	*fdnI*	nitrate-inducible formate dehydrogenase, gamma subunit
PA4811	*fdnH*	nitrate-inducible formate dehydrogenase, beta subunit
PA4812	*fdnG*	formate dehydrogenase-O, major subunit
PA4841		conserved hypothetical protein
PA4874		conserved hypothetical protein
PA4876	*osmE*	osmotically inducible lipoprotein OsmE
PA4880		probable bacterioferritin
PA4913		probable binding protein component of ABC transporter
PA4915		probable chemotaxis transducer
PA4925		conserved hypothetical protein
PA4929		hypothetical protein
PA5039	*aroK*	shikimate kinase

PA5062		conserved hypothetical protein
PA5097		probable amino acid permease
PA5101		hypothetical protein
PA5148		conserved hypothetical protein
PA5153		amino acid (lysine/arginine/ornithine/histidine/octopine) ABC transporter periplasmic binding protein
PA5163	*rmlA*	glucose-1-phosphate thymidylyltransferase
PA5178		conserved hypothetical protein
PA5208		conserved hypothetical protein
PA5213	*gcvP1*	glycine cleavage system protein P1
PA5214	*gcvH1*	glycine cleavage system protein H1
PA5245		conserved hypothetical protein
PA5253	*algP*	alginate regulatory protein AlgP
PA5255	*algQ*	Alginate regulatory protein AlgQ
PA5261	*algR*	alginate biosynthesis regulatory protein AlgR
PA5301		probable transcriptional regulator
PA5348		probable DNA-binding protein
PA5350	*rubA2*	Rubredoxin 2
PA5359		hypothetical protein
PA5424		conserved hypothetical protein
PA5482		hypothetical protein
PA5527		hypothetical protein
PA5543		hypothetical protein
PA5545		conserved hypothetical protein
PA5546		conserved hypothetical protein
