# Supplementary material for: In-Vivo Expression Profiling of Pseudomonas aeruginosa Infections Reveals Niche-Specific and Strain-Independent Transcriptional Programs
Source: PLoS One. 2011 Sep 12;6(9):e24235. doi: 10.1371/journal.pone.0024235 (PMC3171414; doi:10.1371/journal.pone.0024235)
Supplement: Table S7 — Differentially expressed P. aeruginosa genes from the in vivo burn wound infections in comparison to planktonic or biofilm in vitro growth as well as to previous studies of iron starvation response of P. aeruginosa PAO1 strain in vitro. (PDF) [file pone.0024235.s007.pdf]

Table S7.

Locus ID	Gene name	Product name	Fold change values: Burn wound v. biofilm	Burn wound v. planktonic	PAO1 low iron v. PAO1 normal iron*
PA0472		probable sigma-70 factor, ECF subfamily	3.9	-	46
PA0672	*hemO*	heme oxygenase	6.8	-	138
PA0707	*toxR*	transcriptional regulator ToxR	4.2	4.3	8
PA1134		hypothetical protein	3.7	3.7	7
PA1300		probable sigma-70 factor, ECF subfamily	5.5	-	46
PA1301		probable transmembrane sensor	4.2	-	46
PA2033		hypothetical protein	3.0	-	95
PA2034		hypothetical protein	4.1	-	95
PA2383		probable transcriptional regulator	4.2	2.7	148
PA2384		hypothetical protein	12.3	3.1	148
PA2385	*pvdQ*	3-oxo-C12-homoserine lactone acylase PvdQ	5.5	5.7	11
PA2386	*pvdA*	L-ornithine N5-oxygenase	4.4	4.2	216
PA2393		probable dipeptidase precursor	3.5	2.8	38
PA2395	*pvdO*	PvdO	3.9	3.8	38
PA2404		hypothetical protein	4.2	2.9	15
PA2405		hypothetical protein	4.6	4.0	15
PA2406		hypothetical protein	-	3.5	15
PA2407		probable adhesion protein	2.5	2.6	15
PA2411		probable thioesterase	15.5	6.1	126
PA2412		conserved hypothetical protein	13.2	5.8	126
PA2413	*pvdH*	L-2,4-diaminobutyrate:2-ketoglutarate 4-aminotransferase, PvdH	4.7	3.7	65
PA2424	*pvdL*	PvdL	4.6	3.2	34
PA2425	*pvdG*	PvdG	3.8	2.3	34
PA2426	*pvdS*	sigma factor PvdS	10.4	5.4	177
PA2531		probable aminotransferase	-	2.3	11
PA3397	*fpr*	ferredoxin--NADP+ reductase	-	2.9	119
PA3407	*hasAp*	heme acquisition protein HasAp	33.3	31.4	70
PA3530		conserved hypothetical protein	2.7	-	203
PA3811	*hscB*	heat shock protein HscB	-	2.3	4
PA3812	*iscA*	probable iron-binding protein IscA	-	2.5	4
PA4175	*piv*	protease IV	5.6	-	7
PA4220		hypothetical protein	7.5	-	182
PA4221	*fptA*	Fe(III)-pyochelin outer membrane receptor precursor	3.7	-	182
PA4223		probable ATP-binding component of ABC transporter	2.9	-	55
PA4224	*pchG*	pyochelin biosynthetic protein PchG	3.8	-	55
PA4227	*pchR*	transcriptional regulator PchR	5.2	-	37
PA4230	*pchB*	salicylate biosynthesis protein PchB	4.3	-	124
PA4370	*icmP*	Insulin-cleaving metalloproteinase outer membrane protein precursor	2.9	-	19
PA4467		hypothetical protein	10.1	-	119
PA4468	*sodM*	superoxide dismutase	16.9	3.6	119
PA4469		hypothetical protein	10.0	-	119
PA4470	*fumC1*	fumarate hydratase	15.7	-	119

PA4471		hypothetical protein	7.6	3.2	119
PA4570		hypothetical protein	14.0	-	403
PA4833		conserved hypothetical protein	5.8	-	4
PA4896		probable sigma-70 factor, ECF subfamily	3.5	3.4	20
PA5150		probable short-chain dehydrogenase	3.6	-	10
PA5217		probable binding protein component of ABC iron transporter	2.7	-	18
PA5531	*tonB*	TonB protein	3.1	-	24

* iron-regulated genes *in vitro* (12)
