# Supplementary material for: In-Vivo Expression Profiling of Pseudomonas aeruginosa Infections Reveals Niche-Specific and Strain-Independent Transcriptional Programs
Source: PLoS One. 2011 Sep 12;6(9):e24235. doi: 10.1371/journal.pone.0024235 (PMC3171414; doi:10.1371/journal.pone.0024235)
Supplement: Table S8 — Housekeeping genes. The genes with a standard error of probe values below 0.05 and a signal intensity of at least category 3 with 4 being the average signal intensity among all microarrays were defined as housekeeping genes. (PDF) [file pone.0024235.s008.pdf]

Table S8

Locus ID	Gene name	Product name
PA0076		hypothetical protein
PA0077	*icmF1*	IcmF1
PA0129	*gabP*	gamma-aminobutyrate permease
PA0143	*nuh*	purine nucleosidase Nuh
PA0241		probable major facilitator superfamily (MFS) transporter
PA0273		probable major facilitator superfamily (MFS) transporter
PA0309		hypothetical protein
PA0457		hypothetical protein
PA0501	*bioF*	8-amino-7-oxononanoate synthase
PA0706	*cat*	chloramphenicol acetyltransferase
PA0744		probable enoyl-CoA hydratase/isomerase
PA0923	*dinB*	DNA Polymerase IV, DinB
PA1049	*pdxH*	pyridoxine 5'-phosphate oxidase
PA1122		probable peptide deformylase
PA1240		probable enoyl-CoA hydratase/isomerase
PA1278	*cobP*	cobinamide kinase
PA1293		hypothetical protein
PA1319	*cyoC*	cytochrome o ubiquinol oxidase subunit III
PA1418		probable sodium:solute symport protein
PA1433		conserved hypothetical protein
PA1535		probable acyl-CoA dehydrogenase
PA1539		hypothetical protein
PA1585	*sucA*	2-oxoglutarate dehydrogenase (E1 subunit)
PA1638		conserved hypothetical protein
PA1640		conserved hypothetical protein
PA1678		probable DNA methylase
PA1757	*thrH*	homoserine kinase
PA1788		hypothetical protein
PA1816	*dnaQ*	DNA polymerase III, epsilon chain
PA1839		hypothetical protein
PA1948	*rbsC*	membrane protein component of ABC ribose transporter
PA2437		hypothetical protein
PA2611	*cysG*	siroheme synthase
PA2726		probable radical activating enzyme
PA2842		hypothetical protein
PA2877		probable transcriptional regulator
PA2908	*cbiD*	cobalamin biosynthetic protein CbiD
PA2958		hypothetical protein
PA2961	*holB*	DNA polymerase III, delta prime subunit
PA2995	*nqrE*	Na+-translocating NADH:quinone oxidoreductase subunit Nqr5
PA3059	*pelF*	PelF
PA3110		hypothetical protein
PA3165	*hisC2*	histidinol-phosphate aminotransferase
PA3169		5-methylthioribose-1-phosphate isomerase MtnA
PA3268		probable TonB-dependent receptor
PA3368		probable acetyltransferase
PA3493		conserved hypothetical protein
PA3627	*ygbB*	2C-methyl-D-erythritol 2,4-cyclodiphosphate synthase
PA3631		conserved hypothetical protein
PA3693		conserved hypothetical protein

PA3988		hypothetical protein
PA3995		probable transcriptional regulator
PA3998		conserved hypothetical protein
PA4177		hypothetical protein
PA4190	*pqsL*	probable FAD-dependent monooxygenase
PA4355		probable major facilitator superfamily (MFS) transporter
PA4388		hypothetical protein
PA4402	*argJ*	glutamate N-acetyltransferase
PA4908		hypothetical protein
PA5012	*waaF*	heptosyltransferase II
PA5038	*aroB*	3-dehydroquinate synthase
PA5050	*priA*	primosomal protein N'
PA5066	*hisI*	phosphoribosyl-AMP cyclohydrolase
PA5193	*yrfI*	heat shock protein HSP33
PA5280	*sss*	site-specific recombinase Sss
PA5308	*lrp*	leucine-responsive regulatory protein
PA5451	*wzm*	membrane subunit of A-band LPS efflux transporter
PA5552	*glmU*	glucosamine-1-phosphate
Pae_16		16S RNA
Pae_23		23S RNA
