# Supplementary material for: In-Vivo Expression Profiling of Pseudomonas aeruginosa Infections Reveals Niche-Specific and Strain-Independent Transcriptional Programs
Source: PLoS One. 2011 Sep 12;6(9):e24235. doi: 10.1371/journal.pone.0024235 (PMC3171414; doi:10.1371/journal.pone.0024235)
Supplement: Table S9 — Pairwise comparison of the genes present in the specific clusters. Each condition, bw – burn wound infection, tu – tumor infection, pl – plant infection, bf – biofilm growth and lb – planktonic growth, was compared with each other. If the gene was differentially regulated in the comparison the “+” or “−” was given. For example if the gene in the bw/bf comparison has “+”, that means the gene was upregulated in burn wound infection as compared to the biofilm growht. Similarly in case of the “−” the gene would be downregulated in the burn wound as compared to the biofilm. The genes mentioned in the manuscript main text are highlighted in bald. (PDF) [file pone.0024235.s009.pdf]

Table S9

Burn wound infection cluster

	bw /bf	tu/ bf	pl/ bf	bw/ lb	tu/ lb	pl/ lb	pl/ bw	pl/ tu	tu/ bw	bf /lb	Gene name	Product name
PA0059	+	+	+	+			-		-	-	*osmC*	osmotically inducible protein OsmC
PA0060	+			+			-		-			conserved hypothetical protein
PA0102	+	+	+	+	+	+	-					probable carbonic anhydrase
PA0104	+			+								hypothetical protein
PA0266										+	*gabT*	4-aminobutyrate aminotransferase
PA0320	+	+		+	+		-	-				conserved hypothetical protein
PA0354				+			-					conserved hypothetical protein
**PA0355**	+	+				-	-	-		-	***pfpI***	**protease PfpI**
PA0376	+										*rpoH*	sigma factor RpoH
PA0423				+			-				*pasP*	PasP
PA0532	+			+			-		-			hypothetical protein
PA0553	+					-	-			-		hypothetical protein
PA0567	+				-	-	-		-	-		conserved hypothetical protein
PA0672	+						-		-		*hemO*	heme oxygenase
**PA0707**	+			+			-		-		***toxR***	**transcriptional regulator ToxR**
PA0763	+						-				*mucA*	anti-sigma factor MucA
PA0779				+			-					probable ATP-dependent protease
PA1134	+			+					-			hypothetical protein
PA1245					-	-					*aprX*	AprX
PA1320							-				*cyoD*	cytochrome o ubiquinol oxidase subunit IV
PA1323	+	+	+							-		hypothetical protein
PA1324	+			+			-		-			hypothetical protein
PA1500							-					probable oxidoreductase
PA1515	+			+			-				*alc*	allantoicase
PA1518	+			+			-		-			conserved hypothetical protein
PA1579	+								-			hypothetical protein
PA1596			-	+			-	-			*htpG*	heat shock protein HtpG
PA1870	+			+			-		-			hypothetical protein
PA1925					+			-				hypothetical protein

PA2000	+			+			-				*dhcB*	DhcB, dehydrocarnitine CoA transferase, subunit B
PA2033	+											hypothetical protein
PA2034	+			-	-	-			-	-		hypothetical protein
PA2108	+			+			-					probable decarboxylase
PA2116			-							+		conserved hypothetical protein
**PA2134**	+	+	+	+			-		-	-		**hypothetical protein**
**PA2135**	+			+			-		-			**probable transporter**
**PA2140**	+			+			-		-			**probable metallothionein**
**PA2141**	+			+			-		-			**hypothetical protein**
**PA2142**	+			+			-		-			**probable short-chain dehydrogenase**
**PA2143**	+	+	+	+			-		-	-		**hypothetical protein**
**PA2144**	+			+			-		-		*glgP*	**glycogen phosphorylase**
**PA2145**	+			+			-					**hypothetical protein**
**PA2146**	+	+	+	+	-			+	-	-		**conserved hypothetical protein**
**PA2147**	+			+			-		-		*katE*	**catalase HPII**
**PA2148**	+			+			-		-			**conserved hypothetical protein**
**PA2149**	+			+		-	-		-	-		**hypothetical protein**
**PA2150**	+			+			-					**conserved hypothetical protein**
**PA2151**	+			+			-		-			**conserved hypothetical protein**
**PA2152**	+		+	+			-		-			**probable trehalose synthase**
**PA2153**	+			+							*glgB*	**1,4-alpha-glucan branching enzyme**
**PA2157**	+			+			-		-			**hypothetical protein**
**PA2158**	+			+			-		-			**probable alcohol dehydrogenase (Zn-dependent)**
**PA2159**	+		+	+	-	-	-		-	-		**conserved hypothetical protein**
**PA2160**	+			+			-		-		*glgX*	**probable glycosyl hydrolase**
**PA2161**	+			+			-				*0*	**hypothetical protein**
**PA2164**	+			+			-					**probable glycosyl hydrolase**
**PA2165**	+			+			-		-		*glgA*	**probable glycogen synthase**
**PA2167**	+						-		-			**hypothetical protein**
**PA2168**	+	+		+			-	-				**hypothetical protein**
**PA2169**	+	+	+	+			-		-	-		**hypothetical protein**
**PA2170**	+	+	+	+			-		-	-		**hypothetical protein**
**PA2171**	+	+		+			-	-	-	-		**hypothetical protein**

**PA2172**	+	+	+	+			-			-		**hypothetical protein**
**PA2173**	+	+	+				-			-		**hypothetical protein**
**PA2176**	+			+			-					**hypothetical protein**
**PA2178**	+			+			-					**hypothetical protein**
**PA2180**	+			+			-		-			**hypothetical protein**
**PA2181**	+			+			-					**hypothetical protein**
**PA2182**	+											**hypothetical protein**
**PA2183**	+			+			-					**hypothetical protein**
**PA2184**	+						-					**conserved hypothetical protein**
**PA2187**	+			+								**hypothetical protein**
**PA2189**	+						-					**hypothetical protein**
**PA2190**	+	+	+	+			-		-	-		**conserved hypothetical protein**
PA2192	+			+			-	-	-			conserved hypothetical protein
PA2383	+		+	+								probable transcriptional regulator
PA2384	+		+	+				+	-	-		hypothetical protein
**PA2385**	+			+			-		-		*pvdQ*	**3-oxo-C12-homoserine lactone acylase PvdQ**
**PA2386**	+			+			-				*pvdA*	**L-ornithine N5-oxygenase**
**PA2393**	+			+			-					**probable dipeptidase precursor**
**PA2394**							-		-		*pvdN*	**PvdN**
PA2405	+		+	+		+			-			hypothetical protein
PA2411	+	+	+	+		+			-			probable thioesterase
PA2412	+	+	+	+		+		+	-			conserved hypothetical protein
**PA2413**	+			+			-		-		*pvdH*	**L-2,4-diaminobutyrate:2-ketoglutarate 4-aminotransferase, PvdH**
**PA2414**	+				-	-	-		-	-		**L-sorbosone dehydrogenase**
**PA2424**	+			+							*pvdL*	**PvdL**
PA2485				+								hypothetical protein
PA2486	+			+			-		-			hypothetical protein
PA2634				+			-				*aceA*	isocitrate lyase AceA
PA2694	+			+			-					probable thioredoxin
PA3041	+	+	+							-		hypothetical protein
PA3042	+					-	-		-			hypothetical protein
PA3126			-	+	+		-	-		+	*ibpA*	heat-shock protein IbpA
PA3231	+	+	+				-		-	-		hypothetical protein

PA3273		+			+			-				hypothetical protein
PA3274	+	+		+			-		-	-		hypothetical protein
PA3283	+			+			-		-			conserved hypothetical protein
**PA3407**	**+**	**+**		**+**	**+**		**-**	**-**	**-**		***hasAp***	**heme acquisition protein HasAp**
PA3459	+	+		+		-	-	-	-	-	*asnB*	probable glutamine amidotransferase
PA3460	+			+			-		-			probable acetyltransferase
PA3461	+	+		+		-	-	-	-	-		conserved hypothetical protein
PA3540	+			+			-		-		*algD*	GDP-mannose 6-dehydrogenase AlgD
PA3541							-				*alg8*	alginate biosynthesis protein Alg8
PA3598	+			+			-		-			conserved hypothetical protein
PA3600	+	+		+	+		-	-	-			conserved hypothetical protein
PA3601	+	+		+	+	+	-	-	-			conserved hypothetical protein
PA3691	+	+		+		-	-			-		hypothetical protein
PA3692	+			+		-	-		-	-	*lptF*	Lipotoxon F, LptF
PA3731	+			+			-					conserved hypothetical protein
PA3795	+			+			-		-			probable oxidoreductase
PA3811				+		+		+	-		*hscB*	heat shock protein HscB
PA3812		-	+	+	-	+		+	-		*iscA*	probable iron-binding protein IscA
PA3814			+	+		+		+	-		*iscS*	L-cysteine desulfurase (pyridoxal phosphate-dependent)
PA3891	+			+			-					probable ATP-binding component of ABC transporter
PA3951	+			+			-					conserved hypothetical protein
PA4061	+											probable thioredoxin
PA4063	+			+			-		-			hypothetical protein
PA4065	+			+			-		-			hypothetical protein
PA4170	+			+			-		-			hypothetical protein
PA4171	+			+			-		-	-		probable protease
PA4172	+			+			-		-			probable nuclease
**PA4175**	**+**		**+**		**-**				**-**	**-**	***piv***	**protease IV**
PA4344	+						-		-			probable hydrolase
PA4345	+						-					hypothetical protein
PA4387							-				*fxsA*	conserved hypothetical protein
PA4390	+			+			-		-			hypothetical protein
PA4394	+			+			-		-			conserved hypothetical protein

PA4467	+		+		-			+	-	-		hypothetical protein
**PA4468**	**+**		**+**	**+**	**-**	**-**	**-**	**+**	**-**	**-**	***sodM***	**superoxide dismutase**
PA4469	+		+		-	-	-		-	-		hypothetical protein
**PA4470**	**+**		**+**		**-**		**-**	**+**	**-**	**-**	***fumC1***	**fumarate hydratase**
PA4570	+		+		-		-	+	-	-		hypothetical protein
**PA4661**	**+**					**-**	**-**		**-**		***pagL***	**Lipid A 3-O-deacylase**
PA4738									-			conserved hypothetical protein
PA4739									-			conserved hypothetical protein
PA4761	+	+		+	+		-	-			*dnaK*	DnaK protein
PA4834	+			+			-		-			hypothetical protein
PA4835	+			+			-		-			hypothetical protein
PA4836	+			+			-		-			hypothetical protein
PA4837	+			+			-		-			probable outer membrane protein precursor
PA4838	+			+			-					hypothetical protein
PA4877	+	+					-	-		-		hypothetical protein
PA4896	+			+			-		-			probable sigma-70 factor, ECF subfamily
PA4936	+										*spoU*	probable rRNA methylase
PA5053			-	+			-				*hslV*	heat shock protein HslV
PA5054			-	+	+		-	-		+	*hslU*	heat shock protein HslU
PA5060	+									-	*phaF*	polyhydroxyalkanoate synthesis protein PhaF
PA5150	+		+									probable short-chain dehydrogenase
PA5340				+		+				+		hypothetical protein
PA5388	+	+		+	+		-	-	-			hypothetical protein
PA5481	+		+				-			-		hypothetical protein
PA5499	+			+			-		-		*np20*	transcriptional regulator np20
PA5501										+	*znuB*	permease of ABC zinc transporter ZnuB
PA5534				+			-		-			hypothetical protein
PA5535	+			+			-		-			conserved hypothetical protein
PA5536	+			+			-		-			conserved hypothetical protein
PA5537	+			+			-		-			hypothetical protein
PA5538	+			+			-		-		*amiA*	N-acetylmuramoyl-L-alanine amidase
PA5539	+			+			-		-			hypothetical protein
PA5540	+			+			-		-			hypothetical protein

PA5541	+			+			-		-		*pyrQ*	dihydroorotase

Plant infection cluster

	bw /bf	tu/ bf	pl/ bf	bw/ lb	tu/ lb	pl/ lb	pl/ bw	pl/ tu	tu/ bw	bf/ lb		
PA0011						+	+					probable 2-OH-lauroyltransferase
PA0035			+			+	+				*trpA*	tryptophan synthase alpha chain
PA0036							+				*trpB*	tryptophan synthase beta chain
PA0201			+			+	+					hypothetical protein
PA0226						+						probable CoA transferase, subunit A
PA0276		+	+		+	+	+		+			hypothetical protein
**PA0280**			+			+	+	+			***cysA***	**sulfate transport protein CysA**
**PA0281**			+			+	+	+			***cysW***	**sulfate transport protein CysW**
**PA0282**			+			+	+				***cysT***	**sulfate transport protein CysT**
**PA0283**			+			+	+	+			***sbp***	**sulfate-binding protein precursor**
PA0284			+			+	+	+				hypothetical protein
PA0286			+			+	+	+			*desA*	delta-9 fatty acid desaturase, DesA
**PA0296**								+			***spuI***	**probable glutamine synthetase**
**PA0298**			+			+	+	+			***spuB***	**probable glutamine synthetase**
PA0385	-	-				+	+	+		+		hypothetical protein
PA0386						+						probable oxidase
PA0436						+		+				probable transcriptional regulator
PA0439			+			+	+					probable oxidoreductase
PA0441			+			+	+	+			*dht*	dihydropyrimidinase
PA0444						+	+	+				N-carbamoyl-beta-alanine amidohydrolase
PA0451			+			+	+	+				conserved hypothetical protein
PA0456	-	-	+			+	+	+	-	+		probable cold-shock protein
PA0472	+		+		-	+		+	-	-	*fiuI*	probable sigma-70 factor, ECF subfamily
PA0473							+				*psfA*	probable glutathione S-transferase
PA0485						+	+					conserved hypothetical protein
PA0505		-	+	-	-	+	+	+	-	-		hypothetical protein
PA0547								+				probable transcriptional regulator
PA0608	-					+				+	*gph*	probable phosphoglycolate phosphatase

PA0676						+	+				*vreR*	sigma factor regulator, VreR
PA0730						+	+					probable transferase
**PA0734**			+			+	+	+				**hypothetical protein**
PA0801			+				+					hypothetical protein
PA0802			+		-	+	+	+				hypothetical protein
PA0810			+									probable haloacid dehalogenase
PA0814			+			+	+	+				conserved hypothetical protein
PA0815			+			+	+	+				probable transcriptional regulator
PA0817			+			+	+					probable ring-cleaving dioxygenase
PA0837			+			+	+	+			*slyD*	peptidyl-prolyl cis-trans isomerase SlyD
PA0851			+			+	+					hypothetical protein
PA0862			+									hypothetical protein
PA0929		-	+		-	+	+	+	-		*pirR*	two-component response regulator
PA0936		-				+	+	+			*lpxO2*	lipopolysaccharide biosynthetic protein LpxO2
PA0995			+			+		+			*ogt*	methylated-DNA--protein-cysteine methyltransferase
PA1060			+			+	+	+				hypothetical protein
PA1075			+				+					hypothetical protein
PA1092	-						+		+		*fliC*	flagellin type B
PA1101							+				*fliF*	Flagella M-ring outer membrane protein precursor
PA1190			+	-	-	+	+	+		-		conserved hypothetical protein
PA1192						+						conserved hypothetical protein
PA1296						+		+				probable 2-hydroxyacid dehydrogenase
PA1377			+				+					conserved hypothetical protein
PA1440			+				+	+				hypothetical protein
PA1452							+				*flhA*	flagellar biosynthesis protein FlhA
PA1505			+			+	+	+			*moaA2*	molybdopterin biosynthetic protein A2
PA1626			+			+						probable major facilitator superfamily (MFS) transporter
PA1630			+			+	+					probable transcriptional regulator
PA1632						+					*kdpF*	KdpF protein
PA1651			+			+	+	+				probable transporter
PA1676			+				+	+		-		hypothetical protein
PA1677			+	-		+	+	+		-		conserved hypothetical protein
PA1730			+									conserved hypothetical protein

PA1775			+		+		+	-		*cmpX*	conserved cytoplasmic membrane protein, CmpX protein
PA1776	-			-			+			*sigX*	ECF sigma factor SigX
PA1778		+			+	+	+			*cobA*	uroporphyrin-III C-methyltransferase
**PA1779**		+			+	+					**assimilatory nitrate reductase**
**PA1780**		+			+	+	+			***nirD***	**assimilatory nitrite reductase small subunit**
**PA1781**		+			+	+				***nirB***	**assimilatory nitrite reductase large subunit**
**PA1782**					+	+					**probable serine/threonine-protein kinase**
**PA1783**		+			+					***nasA***	**nitrate transporter**
PA1800					+		+			*tig*	trigger factor
PA1814		+				+	+				hypothetical protein
PA1837		+			+	+					hypothetical protein
**PA1838**		+			+	+	+			***cysI***	**sulfite reductase**
PA1856		+			+	+	+				probable cytochrome oxidase subunit
PA1927		+			+	+	+			*metE*	5-methyltetrahydropteroyltriglutamate-homocysteine S-methyltransferase
PA1950		+				+				*rbsK*	ribokinase
PA1963		+			+	+	+				hypothetical protein
PA1984		+			+	+	+		+	*exaC*	NAD+ dependent aldehyde dehydrogenase ExaC
PA1998		+			+					*dhcR*	DhcR, transcriptional regulator
PA2019					+	+	+			*amrA*	Resistance-Nodulation-Cell Division (RND) multidrug efflux membrane fusion protein precursor
PA2030		+	-		+	+	+		-		hypothetical protein
PA2031	+	+			+	+	+				hypothetical protein
PA2045		+			+	+	+				conserved hypothetical protein
PA2092		+			+						probable major facilitator superfamily (MFS) transporter
PA2229		+			+	+					conserved hypothetical protein
PA2259		+			+	+	+			*ptxS*	transcriptional regulator PtxS
PA2260		+			+	+	+				hypothetical protein
PA2261		+			+	+					probable 2-ketogluconate kinase
PA2263		+			+	+	+				probable 2-hydroxyacid dehydrogenase
PA2306		+			+	+	+			*ambA*	AmbA
PA2322		+			+	+					gluconate permease
PA2380		+			+	+	+				hypothetical protein
PA2484		+				+	+				conserved hypothetical protein

PA2491			+				+	+				probable oxidoreductase
**PA2519**			+			+	+	+			***xylS***	**transcriptional regulator XylS**
PA2560			+			+	+	+				hypothetical protein
PA2570	-		+	-		+	+	+			*lecA*	LecA
PA2582			+				+	+				hypothetical protein
PA2662		+	+		+	+	+	+	+			conserved hypothetical protein
PA2663			+			+	+	+			*ppyR*	psl and pyoverdine operon regulator, PpyR
PA2664			+			+	+	+			*fhp*	flavohemoprotein
PA2710			+				+	+				hypothetical protein
PA2718			+									probable transcriptional regulator
PA2743						+	+	+			*infC*	translation initiation factor IF-3
PA2746			+	-	-	+	+	+		-		hypothetical protein
PA2759			+			+	+	+				hypothetical protein
PA2786			+			+	+	+				hypothetical protein
PA2844			+									conserved hypothetical protein
PA2847			+			+	+	+				conserved hypothetical protein
PA2876							+				*pyrF*	orotidine 5'-phosphate decarboxylase
PA2906						+						probable oxidoreductase
PA2950						+						hypothetical protein
PA2985			+			+	+	+				hypothetical protein
PA3009	-	-				+	+	+	-	+		hypothetical protein
PA3010							+	+				hypothetical protein
PA3034			+			+						probable transcriptional regulator
PA3167						+	+	+			*serC*	3-phosphoserine aminotransferase
PA3168						+	+	+		+	*gyrA*	DNA gyrase subunit A
PA3178			+				+	+		-		hypothetical protein
PA3182			+			+	+				*pgl*	6-phosphogluconolactonase
PA3183			+				+				*zwf*	glucose-6-phosphate 1-dehydrogenase
PA3195			+				+	+			*gapA*	glyceraldehyde 3-phosphate dehydrogenase
PA3266		-		+		+	+	+	-	+	*capB*	cold acclimation protein B
PA3285			+			+						probable sigma-70 factor, ECF subfamily
PA3354			+	-			+			-		hypothetical protein
PA3417		+	+	-			+		+	-		probable pyruvate dehydrogenase E1 component, alpha subunit

PA3436					+	+					hypothetical protein
PA3450		+			+	+	+				probable antioxidant protein
PA3473					+	+					hypothetical protein
PA3533	-	+		-	+	+	+	-			conserved hypothetical protein
PA3536		+				+	+				hypothetical protein
PA3576		+	-			+	+				hypothetical protein
PA3578	+	+		+	+	+					conserved hypothetical protein
PA3668		+									conserved hypothetical protein
PA3720					+						hypothetical protein
PA3726						+					conserved hypothetical protein
PA3744	-				+		+	-		*rimM*	16S rRNA processing protein
PA3754		+			+	+	+				hypothetical protein
PA3756					+	+	+				hypothetical protein
PA3796	+	+	-			+			-		hypothetical protein
PA3808	-	+		-	+	+	+	-			conserved hypothetical protein
PA3813		+			+	+	+			*iscU*	probable iron-binding protein IscU
PA3817					+						probable methyltransferase
PA3844		+									hypothetical protein
PA3899		+			+		+				probable sigma-70 factor, ECF subfamily
PA3914		+			+		+			*moeA1*	molybdenum cofactor biosynthetic protein A1
PA3915		+			+	+	+			*moaB1*	molybdopterin biosynthetic protein B1
PA3918		+			+	+				*moaC*	molybdopterin biosynthetic protein C
PA4007					+	+				*proA*	gamma-glutamyl phosphate reductase
PA4014						+					hypothetical protein
PA4021		+				+	+				probable transcriptional regulator
PA4026		+				+					probable acetyltransferase
PA4029		+			+		+				conserved hypothetical protein
PA4034		+	+		+	+	+			*aqpZ*	aquaporin Z
PA4044					+	+	+			*dxs*	1-deoxyxylulose-5-phosphate synthase
PA4045					+						conserved hypothetical protein
PA4046					+	+					hypothetical protein
PA4051					+					*thiL*	thiamine monophosphate kinase
PA4069					+						hypothetical protein

PA4147			+			+	+	+			*acoR*	transcriptional regulator AcoR
PA4176			+			+					*ppiC2*	peptidyl-prolyl cis-trans isomerase C2
PA4245		-				+				+	*rpmD*	50S ribosomal protein L30
PA4248		-		+		+		+		+	*rplF*	50S ribosomal protein L6
PA4262						+				+	*rplD*	50S ribosomal protein L4
PA4263		-				+				+	*rplC*	50S ribosomal protein L3
PA4293			+	-			+	+			*pprA*	two-component sensor PprA
PA4326			+			+	+	+				hypothetical protein
PA4379			+		-			+	-			conserved hypothetical protein
**PA4442**			+			+	+				***cysN***	**ATP sulfurylase GTP-binding subunit/APS kinase**
**PA4443**			+			+	+	+			***cysD***	**ATP sulfurylase small subunit**
PA4544						+					*rluD*	pseudouridine synthase
PA4567	-	-			-	+	+	+	-	+	*rpmA*	50S ribosomal protein L27
PA4575	+	+	+				+			-		hypothetical protein
PA4602	-	-				+	+	+		+	*glyA3*	serine hydroxymethyltransferase
PA4630			+			+	+	+				hypothetical protein
PA4637			+			+	+	+				hypothetical protein
PA4666						+					*hemA*	glutamyl-tRNA reductase
**PA4726**			+			+	+	+			***cbrB***	**two-component response regulator CbrB**
PA4742						+				+	*truB*	tRNA pseudouridine 55 synthase
PA4748						+	+			+	*tpiA*	triosephosphate isomerase
PA4750						+					*folP*	dihydropteroate synthase
PA4758						+					*carA*	carbamoyl-phosphate synthase small chain
PA4780			+	-			+	+				conserved hypothetical protein
PA4852						+	+	+				conserved hypothetical protein
PA4859			+			+						probable permease of ABC transporter
PA4870			+			+	+	+				conserved hypothetical protein
PA4881		+	+		+	+	+	+	+			hypothetical protein
PA4933					+	+				+		hypothetical protein
PA4939						+	+	+		+		conserved hypothetical protein
PA4940	-	-			-	+	+	+	-			conserved hypothetical protein
PA5028			+			+	+					conserved hypothetical protein
PA5048			+			+	+	+				probable nuclease

**PA5176**			+			+	+	+				**conserved hypothetical protein**
PA5249			+			+	+					hypothetical protein
PA5287			+			+	+	+			*amtB*	ammonium transporter AmtB
PA5288			+	-	-		+	+		-	*glnK*	nitrogen regulatory protein P-II 2
PA5295						+	+	+				hypothetical protein
PA5298				+		+				+	*xpt*	xanthine phosphoribosyltransferase
PA5337			+			+	+	+			*rpoZ*	RNA polymerase omega subunit
PA5346			+					+			*sadB*	SadB
PA5380			+	-	-		+	+		-	*gbdR*	GbdR
PA5383			+			+	+	+				conserved hypothetical protein
PA5438			+				+	+				probable transcriptional regulator
PA5446						+	+	+				hypothetical protein
PA5460			+			+	+	+				hypothetical protein
PA5468			+			+	+	+				probable citrate transporter
PA5504	-	-				+	+			+		D-methionine ABC transporter membrane protein

Tumor infection cluster

	bw /bf	tu/ bf	pl/ bf	bw/ lb	tu/ lb	pl/ lb	pl/ bw	pl/ tu	tu/ bw	bf/ lb		
**PA0044**		+			+			-	+		***exoT***	**exoenzyme T**
PA0046								-				hypothetical protein
PA0067		+									*prlC*	oligopeptidase A
PA0093		+			+			-				hypothetical protein
PA0132		+			+			-	+		*oapT*	beta-alanine--pyruvate transaminase
PA0141		+			+			-	+			conserved hypothetical protein
PA0396								-	+		*pilU*	twitching motility protein PilU
PA0415		+			+			-			*chpC*	probable chemotaxis protein
PA0442							-	-				hypothetical protein
PA0510		+			+			-	+		*nirE*	probable uroporphyrin-III c-methyltransferase
**PA0517**					+			-	+		***nirC***	probable c-type cytochrome precursor
**PA0518**		+			+			-	+		***nirM***	cytochrome c-551 precursor
**PA0519**		+			+			-	+		***nirS***	nitrite reductase precursor

PA0534								-				conserved hypothetical protein
PA0545		+			+			-	+			hypothetical protein
PA0572					+			-				hypothetical protein
PA0713	-	+	-		+			-	+			hypothetical protein
PA0715							-	-				hypothetical protein
PA0716								-				hypothetical protein
PA0781	+	+		+	+		-	-				hypothetical protein
PA0822							-	-				hypothetical protein
PA0835								-	+		*pta*	phosphate acetyltransferase
**PA0836**		**+**			**+**			**-**	**+**		***ackA***	**acetate kinase**
PA0864		+			+			-	+			probable transcriptional regulator
PA0874								-				hypothetical protein
PA0952	-		-		+			-	+	+		hypothetical protein
PA0962	+	+				-	-	-				probable dna-binding stress protein
PA0977								-				hypothetical protein
PA0985							-	-			*pyoS5*	pyocin S5
PA1029	+	+	+		+			-	+			hypothetical protein
PA1126					+			-				hypothetical protein
PA1127		+						-				probable oxidoreductase
PA1131		-					+	+				probable major facilitator superfamily (MFS) transporter
PA1195		+			+			-	+			hypothetical protein
PA1329								-				conserved hypothetical protein
PA1337		+									*ansB*	glutaminase-asparaginase
PA1369							-	-				hypothetical protein
PA1370								-				hypothetical protein
PA1371							-	-				hypothetical protein
PA1372							-	-				hypothetical protein
PA1388		+			+			-	+			hypothetical protein
PA1429		+			+			-	+			probable cation-transporting P-type ATPase
PA1555	-		-		+		-	-	+	+	*ccoP2*	Cytochrome c oxidase, cbb3-type, CcoP subunit
PA1557	-		-		+			-	+	+	*ccoN2*	Cytochrome c oxidase, cbb3-type, CcoN subunit
PA1664							-	-				hypothetical protein
PA1673		+			+		-	-	+			hypothetical protein

PA1692					+						*pscS*	probable translocation protein in type III secretion
**PA1696**					+			-			***pscO***	**translocation protein in type III secretion**
**PA1699**					+			-	+		***pcr1***	**Pcr1**
**PA1700**		+			+			-	+		***pcr2***	**Pcr2**
**PA1701**	-		-		+			-	+	+	***pcr3***	**Pcr3**
**PA1706**		+			+			-	+		***pcrV***	**type III secretion protein PcrV**
**PA1707**		+			+			-	+		***pcrH***	**regulatory protein PcrH**
**PA1708**		+			+			-	+		***popB***	**translocator protein PopB**
**PA1709**		+			+			-	+		***popD***	**Translocator outer membrane protein PopD precursor**
**PA1711**		+			+			-	+		***exsE***	**ExsE**
**PA1714**	-		-		+			-	+		***exsD***	**ExsD**
**PA1715**		+			+			-	+		***pscB***	**type III export apparatus protein**
**PA1716**					+			-	+		***pscC***	**Type III secretion outer membrane protein PscC precursor**
**PA1717**		+			+			-	+		***pscD***	**type III export protein PscD**
**PA1718**		+			+			-	+		***pscE***	**type III export protein PscE**
**PA1719**		+			+		-	-	+		***pscF***	**type III export protein PscF**
**PA1720**		+			+			-			***pscG***	**type III export protein PscG**
**PA1721**		+			+			-	+		***pscH***	**type III export protein PscH**
**PA1722**		+			+			-	+		***pscI***	**type III export protein PscI**
**PA1723**					+			-			***pscJ***	**type III export protein PscJ**
**PA1724**					+			-			***pscK***	**type III export protein PscK**
PA1734					+			-				hypothetical protein
PA1746		+			+			-	+			hypothetical protein
PA1789		+						-	+			hypothetical protein
PA1869	-	-						+				probable acyl carrier protein
PA1901						-					*phzC2*	phenazine biosynthesis protein PhzC
PA1904								-			*phzF2*	probable phenazine biosynthesis protein
PA1934								-				hypothetical protein
PA2037								-				hypothetical protein
PA2102								-				hypothetical protein
PA2119		+			+			-	+		*adh*	alcohol dehydrogenase (Zn-dependent)
PA2127					+			-	+			conserved hypothetical protein
**PA2128**		+			+			-	+		***cupA1***	**fimbrial subunit CupA1**

PA2186				+			-	-				hypothetical protein
**PA2191**		+			+			-	+		***exoY***	**adenylate cyclase ExoY**
PA2392		+			+			-			*pvdP*	PvdP
PA2436		+			+			-				hypothetical protein
PA2460		+			+							hypothetical protein
PA2461							-	-				hypothetical protein
PA2576		+			+			-				hypothetical protein
PA2730								-				hypothetical protein
PA2733							-	-				conserved hypothetical protein
PA2734								-				hypothetical protein
PA2753		+			+			-	+			hypothetical protein
PA2819							-	-				hypothetical protein
PA2825								-			*ospR*	OspR
PA2826		+						-				probable glutathione peroxidase
PA2931		+			+			-	+		*cifR*	CifR
PA3115					+			-	+		*fimV*	Motility protein FimV
PA3140								-				hypothetical protein
PA3144		+										hypothetical protein
**PA3147**					+			-	+		***wbpJ***	**probable glycosyl transferase WbpJ**
**PA3148**		+			+			-	+		***wbpI***	**UDP-N-acetylglucosamine 2-epimerase WbpI**
**PA3149**					+			-			***wbpH***	**probable glycosyltransferase WbpH**
**PA3150**		+			+			-	+		***wbpG***	**LPS biosynthesis protein WbpG**
**PA3151**		+			+			-			***hisF2***	**imidazoleglycerol-phosphate synthase, cyclase subunit**
**PA3152**		+			+			-	+		***hisH2***	**glutamine amidotransferase**
**PA3154**		+			+			-	+		***wzy***	**B-band O-antigen polymerase**
**PA3155**					+			-			***wbpE***	**UDP-2-acetamido-2-dideoxy-d-ribo-hex-3-uluronic acid transaminase, wbpE**
**PA3156**					+						***wbpD***	**UDP-2-acetamido-3-amino-2,3-dideoxy-d-glucuronic acid N-acetyltransferase, WbpD**
**PA3157**									+			**probable acetyltransferase**
**PA3159**					+						***wbpA***	**UDP-N-acetyl-d-glucosamine 6-Dehydrogenase**
**PA3160**					+				+		***wzz***	**O-antigen chain length regulator**
PA3161			+				+			-	*himD*	integration host factor beta subunit
PA3278	-	+			+			-	+	+		hypothetical protein

PA3291								-				hypothetical protein
PA3309		+			+	-		-	+			conserved hypothetical protein
PA3337		+			+			-			*rfaD*	ADP-L-glycero-D-mannoheptose 6-epimerase
PA3431	+	+	+	+	+	+		-	+			conserved hypothetical protein
PA3432	+	+	+	+	+	+		-	+			hypothetical protein
PA3458						-		-				probable transcriptional regulator
PA3520	-			-								hypothetical protein
PA3529		+										probable peroxidase
PA3572	-	+	+	-	+		+	-	+	-		hypothetical protein
PA3613		+			+			-	+			hypothetical protein
PA3615										-		hypothetical protein
**PA3841**		+			+	-		-	+		***exoS***	**exoenzyme S**
PA3842		+			+			-	+		*spcS*	specific Pseudomonas chaperone for ExoS, SpcS
PA3866		+					-	-				pyocin protein
PA3880		+			+			-	+			conserved hypothetical protein
PA3968		+			+			-				probable pseudouridine synthase
PA4028				+	+		-	-				hypothetical protein
PA4067			-		+			-	+		*oprG*	Outer membrane protein OprG precursor
PA4141	-	-	-							+		hypothetical protein
PA4211					+			-	+		*phzB1*	probable phenazine biosynthesis protein
PA4217					+			-			*phzS*	flavin-containing monooxygenase
PA4348			-		+			-	+	+		conserved hypothetical protein
**PA4352**	+	+	+		+			-	+	-		**conserved hypothetical protein**
PA4385		+	-	+	+		-	-			*groEL*	GroEL protein
PA4386		+	-		+	-	-	-			*groES*	GroES protein
PA4407			-			-	-	-		-	*ftsZ*	cell division protein FtsZ
PA4463			+	-						-		conserved hypothetical protein
PA4577		+			+			-	+			hypothetical protein
PA4578			-									hypothetical protein
PA4587		+	-		+			-	+	+	*ccpR*	cytochrome c551 peroxidase precursor
PA4610		+			+			-	+			hypothetical protein
PA4699		+			+			-				hypothetical protein
PA4760							-	-			*dnaJ*	DnaJ protein

PA4922			-					-			*azu*	azurin precursor
PA5027		+			+			-	+			hypothetical protein
PA5044			-					-	+		*pilM*	type 4 fimbrial biogenesis protein PilM
PA5086								-				hypothetical protein
PA5087							-	-				hypothetical protein
PA5105		+			+			-	+		*hutC*	histidine utilization repressor HutC
PA5106		+			+			-	+			conserved hypothetical protein
**PA5170**	**+**	**+**	**+**					**-**	**+**	**-**	***arcD***	**arginine/ornithine antiporter**
**PA5171**	**+**	**+**			**+**	**-**	**-**	**-**	**+**		***arcA***	**arginine deiminase**
**PA5172**	**+**	**+**			**+**	**-**	**-**	**-**	**+**	**-**	***arcB***	**ornithine carbamoyltransferase, catabolic**
**PA5173**	**+**	**+**				**-**	**-**	**-**		**-**	***arcC***	**carbamate kinase**
PA5232		+			+	-		-	+			conserved hypothetical protein
PA5427		+			+		-	-	+		*adhA*	alcohol dehydrogenase
PA5475		+			+			-	+			hypothetical protein
PA5494		+	+	-					+	-		hypothetical protein

Biofilm growth specific cluster

	bw /bf	tu/ bf	pl/ bf	bw/ lb	tu/ lb	pl/ lb	pl/ bw	pl/ tu	tu/ bw	bf/ lb		
PA0089			-								*tssG1*	TssG1
PA0128	-	-	-							+		conserved hypothetical protein
PA0140			-					-		+	*ahpF*	alkyl hydroperoxide reductase subunit F
PA0161	-	-	-							+		hypothetical protein
PA0172			-									hypothetical protein
PA0257		-	-									hypothetical protein
PA0261			-									hypothetical protein
PA0263	-	-	-			+		+		+	*hcpC*	secreted protein Hcp
PA0291	-	-	-							+	*oprE*	Anaerobically-induced outer membrane porin OprE precursor
PA0297		-				+	+	+		+	*spuA*	probable glutamine amidotransferase
PA0359			-					-				hypothetical protein
PA0363	-	-	-								*coaD*	phosphopantetheine adenylyltransferase
PA0381										+	*thiG*	thiamine biosynthesis protein, thiazole moiety

PA0408	-	-	-								*pilG*	twitching motility protein PilG
PA0409	-	-	-							+	*pilH*	twitching motility protein PilH
PA0410	-	-	-							+	*pilI*	twitching motility protein PilI
PA0411	-		-							+	*pilJ*	twitching motility protein PilJ
PA0424		-	-	+					-	+	*mexR*	multidrug resistance operon repressor MexR
PA0506	-		-		+					+		probable acyl-CoA dehydrogenase
PA0541	-	-	-							+		hypothetical protein
PA0563	-	-				+	+	+		+		conserved hypothetical protein
PA0576		-	-						-	+	*rpoD*	sigma factor RpoD
PA0578	-	-	-	+		+		+	-	+		conserved hypothetical protein
PA0579	-	-		+		+		+	-	+	*rpsU*	30S ribosomal protein S21
PA0581	-	-	-							+		conserved hypothetical protein
PA0589	-	-	-							+		conserved hypothetical protein
PA0595		-	-							+	*ostA*	organic solvent tolerance protein OstA precursor
**PA0652**			**-**	**+**						**+**	***vfr***	**transcriptional regulator Vfr**
PA0654	-	-	-							+	*speD*	S-adenosylmethionine decarboxylase proenzyme
PA0655	-	-	-									hypothetical protein
PA0667	-	-	-									conserved hypothetical protein
PA0767	-	-	-							+	*lepA*	GTP-binding protein LepA
PA0768	-	-	-							+	*lepB*	signal peptidase I
PA0805	-	-			-	+	+	+	-	+		hypothetical protein
PA0915	-	-	-							+		conserved hypothetical protein
PA0916		-								+		conserved hypothetical protein
PA0944		-	-							+	*purN*	phosphoribosylaminoimidazole synthetase
PA0945	-	-								+	*purM*	phosphoribosylaminoimidazole synthetase
PA0955	-	-	-							+		hypothetical protein
PA0961			-							+		probable cold-shock protein
PA0965	-	-					+				*ruvC*	Holliday junction resolvase RuvC
PA0974		-	-							+		conserved hypothetical protein
PA0981	-		-					-				hypothetical protein
**PA0996**		**-**			**-**			**+**			***pqsA***	**probable coenzyme A ligase**
**PA0998**	**-**	**-**	**-**		**-**						***pqsC***	**PqsC**
PA1006		-										conserved hypothetical protein

PA1034	-	-	-							+		hypothetical protein
PA1080	-	-	-			-					*flgE*	flagellar hook protein FlgE
PA1094									+		*fliD*	flagellar capping protein FliD
PA1123	-	-	-							+		hypothetical protein
PA1132	-	-	-							+		hypothetical protein
PA1159			+			+	+	+				probable cold-shock protein
PA1168	-	-	-	-	-	-				+		hypothetical protein
PA1183	-	-	-			+	+	+		+	*dctA*	C4-dicarboxylate transport protein
PA1193		-	-							+		hypothetical protein
PA1439	-	-										conserved hypothetical protein
PA1477	-	-								+	*ccmC*	heme exporter protein CcmC
PA1479	-	-								+	*ccmE*	cytochrome C-type biogenesis protein CcmE
PA1480	-	-	-							+	*ccmF*	cytochrome C-type biogenesis protein CcmF
PA1482	-	-	-							+	*ccmH*	cytochrome C-type biogenesis protein CcmH
PA1541										+		probable drug efflux transporter
PA1543	-	-								+	*apt*	adenine phosphoribosyltransferase
PA1546	-				+				+		*hemN*	oxygen-independent coproporphyrinogen III oxidase
PA1551	-		-							+		probable ferredoxin
PA1556	-		-	+	+		-	-		+	*ccoO2*	Cytochrome c oxidase, cbb3-type, CcoO subunit
PA1564		-						+	-	+		conserved hypothetical protein
PA1580		-	-				-				*gltA*	citrate synthase
PA1582				+		+				+	*sdhD*	succinate dehydrogenase (D subunit)
PA1584	-	-	-							+	*sdhB*	succinate dehydrogenase (B subunit)
PA1610	-	-			-	+		+	-	+	*fabA*	beta-hydroxydecanoyl-ACP dehydrase
PA1656	-	-	-							+		hypothetical protein
PA1657	-	-	-							+		conserved hypothetical protein
PA1658	-	-	-							+		conserved hypothetical protein
PA1659	-	-	-					-		+		hypothetical protein
PA1660	-	-	-							+		hypothetical protein
PA1661	-	-	-							+		hypothetical protein
PA1668	-		-							+		hypothetical protein
PA1669	-		-							+		hypothetical protein
PA1713	-		-					-		+	*exsA*	transcriptional regulator ExsA

PA1767	-	-	-							+		hypothetical protein
PA1768	-	-	-	+						+		hypothetical protein
PA1774		-			-			+	-	+	*crfX*	CrfX protein
PA1801		-									*clpP*	ClpP
PA1805			-								*ppiD*	peptidyl-prolyl cis-trans isomerase D
PA1812	-	-	-	+			-			+	*mltD*	membrane-bound lytic murein transglycosylase D precursor
PA1830	-		-							+		hypothetical protein
PA2042		-								+		probable transporter (membrane subunit)
PA2112			-									conserved hypothetical protein
PA2456	-		-									hypothetical protein
PA2459			-				-	-				hypothetical protein
PA2584	-	-				+		+		+	*pgsA*	CDP-diacylglycerol--glycerol-3-phosphate 3-phosphatidyltransferase
PA2593	-	-	-							+	*qteE*	quorum threshold expression element, QteE
PA2619	-	-	-	+		+		+	-	+	*infA*	initiation factor
PA2624			-	+			-		-	+	*idh*	isocitrate dehydrogenase
PA2627		-								+		conserved hypothetical protein
PA2629	-	-				+	+	+		+	*purB*	adenylosuccinate lyase
PA2637											*nuoA*	NADH dehydrogenase I chain A
PA2639			-								*nuoD*	NADH dehydrogenase I chain C,D
PA2666	-					+	+			+	*ptpS*	probable 6-pyruvoyl tetrahydrobiopterin synthase
PA2667			-	+			-			+		conserved hypothetical protein
PA2741		-		+		+		+	-	+	*rplT*	50S ribosomal protein L20
PA2769		-	-									hypothetical protein
PA2840	-	-		+		+				+	*deaD*	probable ATP-dependent RNA helicase
PA2851		-		+		+				+	*efp*	translation elongation factor P
PA2953	-		-							+		electron transfer flavoprotein-ubiquinone oxidoreductase
PA2957	-	-				+	+	+		+		probable transcriptional regulator
PA2959										+		conserved hypothetical protein
PA2960		-	-								*pilZ*	type 4 fimbrial biogenesis protein PilZ
PA2966	-	-				+		+	-	+	*acpP*	acyl carrier protein
PA2970	-	-		+		+		+	-	+	*rpmF*	50S ribosomal protein L32
PA2971	-	-				+				+		conserved hypothetical protein
PA2986										+		conserved hypothetical protein

PA3013			-				-	-		+	*foaB*	fatty-acid oxidation complex beta-subunit
PA3057	-		-									hypothetical protein
PA3112		-							-	+	*accD*	acetyl-CoA carboxylase beta subunit
PA3162		-				+		+		+	*rpsA*	30S ribosomal protein S1
PA3217	-	-	-							+	*cyaB*	CyaB
PA3245	-	-	-							+	*minE*	cell division topological specificity factor MinE
PA3280	-		-							+	*oprO*	Pyrophosphate-specific outer membrane porin OprO precursor
PA3292	-	-	-					-		+		hypothetical protein
PA3299	-		-							+	*fadD1*	long-chain-fatty-acid--CoA ligase
PA3332	-	-	-							+		conserved hypothetical protein
PA3383			-								*phnD*	binding protein component of ABC phosphonate transporter
PA3480		-								+		probable deoxycytidine triphosphate deaminase
PA3489	-	-	-							+		conserved hypothetical protein
PA3525	-	-				+		+		+	*argG*	argininosuccinate synthase
PA3531	-	-	-							+	*bfrB*	bacterioferritin
PA3611				+		+				+		hypothetical protein
PA3612				+					-	+		conserved hypothetical protein
PA3621	-	-				+	+			+	*fdxA*	ferredoxin I
PA3633										+	*ygbP*	4-diphosphocytidyl-2-C-methylerythritol synthase
PA3636		-	-								*kdsA*	2-dehydro-3-deoxyphosphooctonate aldolase
PA3639										+	*accA*	acetyl-coenzyme A carboxylase carboxyl transferase (alpha subunit)
PA3642			-							+	*rnhB*	ribonuclease HII
PA3644			-							+	*lpxA*	UDP-N-acetylglucosamine acyltransferase
PA3645	-	-	-							+	*fabZ*	(3R)-hydroxymyristoyl-[acyl carrier protein] dehydratase
PA3646	-	-	-							+	*lpxD*	UDP-3-O-[3-hydroxylauroyl] glucosamine N-acyltransferase
PA3648	-	-	-							+	*opr86*	outer membrane protein Opr86
PA3653	-		-								*frr*	ribosome recycling factor
PA3654	-	-								+	*pyrH*	uridylate kinase
PA3655		-		+		+				+	*tsf*	elongation factor Ts
PA3656		-		+						+	*rpsB*	30S ribosomal protein S2
PA3686		-	-						-	+	*adk*	adenylate kinase
PA3700										+	*lysS*	lysyl-tRNA synthetase
PA3722	-	-	-							+		hypothetical protein

PA3741	-	-	-							+		hypothetical protein
PA3742	-	-	-	+		+			-	+	*rplS*	50S ribosomal protein L19
PA3743		-				+		+		+	*trmD*	tRNA (guanine-N1)-methyltransferase
PA3745		-				+		+		+	*rpsP*	30S ribosomal protein S16
PA3770		-	-							+	*guaB*	inosine-5'-monophosphate dehydrogenase
PA3804	-	-	-							+		hypothetical protein
PA3805	-	-									*pilF*	type 4 fimbrial biogenesis protein PilF
PA3806	-	-	-						-	+		conserved hypothetical protein
PA3818		-				+		+		+	*suhB*	extragenic suppressor protein SuhB
PA3822			-							+		conserved hypothetical protein
PA3824	-	-	-							+	*queA*	S-adenosylmethionine:trna ribosyltransferase-isomerase
PA3903			-							+	*prfC*	peptide chain release factor 3
PA3905	-	-	-							+		hypothetical protein
PA3906	-	-	-							+		hypothetical protein
PA3907			-							+		hypothetical protein
PA3908	-	-	-							+		hypothetical protein
PA3941	-	-								+		hypothetical protein
PA3966	-	-	-	+		+				+		hypothetical protein
PA3967	-	-	-							+		hypothetical protein
PA3979	-	-				+		+		+		hypothetical protein
PA3980										+		conserved hypothetical protein
PA3982		-				+				+		conserved hypothetical protein
PA3989		-									*holA*	DNA polymerase III, delta subunit
PA4031		-		+		+				+	*ppa*	inorganic pyrophosphatase
PA4130	-	-	-	-								probable sulfite or nitrite reductase
PA4131	-		-					-		+		probable iron-sulfur protein
PA4132	-		-					-		+		conserved hypothetical protein
PA4133	-	-	-	-		-		-	+			cytochrome c oxidase subunit (cbb3-type)
PA4134	-	-	-	-		-				+		hypothetical protein
PA4139	-	-	-	-	-	-	-	-		+		hypothetical protein
PA4140	-	-	-							+		hypothetical protein
PA4142	-	-	-							+		probable secretion protein
PA4237		-	-						-	+	*rplQ*	50S ribosomal protein L17

PA4238		-								+	*rpoA*	DNA-directed RNA polymerase alpha chain
PA4239		-	-	+						+	*rpsD*	30S ribosomal protein S4
PA4240		-	-	+		+				+	*rpsK*	30S ribosomal protein S11
PA4241	-	-		+		+		+		+	*rpsM*	30S ribosomal protein S13
PA4242	-	-									*rpmJ*	50S ribosomal protein L36
PA4243		-								+	*secY*	secretion protein SecY
PA4246		-								+	*rpsE*	30S ribosomal protein S5
PA4247		-	-	+					-	+	*rplR*	50S ribosomal protein L18
PA4249		-								+	*rpsH*	30S ribosomal protein S8
PA4250	-	-				+		+	-	+	*rpsN*	30S ribosomal protein S14
PA4251	-										*rplE*	50S ribosomal protein L5
PA4252			-							+	*rplX*	50S ribosomal protein L24
PA4253			-								*rplN*	50S ribosomal protein L14
PA4254	-	-	-								*rpsQ*	30S ribosomal protein S17
PA4258		-									*rplV*	50S ribosomal protein L22
PA4261	-	-	-							+	*rplW*	50S ribosomal protein L23
PA4264	-	-				+	+	+		+	*rpsJ*	30S ribosomal protein S10
PA4265					+	+				+	*tufA*	elongation factor Tu
PA4266										+	*fusA1*	elongation factor G
PA4268	-					+	+	+			*rpsL*	30S ribosomal protein S12
PA4269	-									+	*rpoC*	DNA-directed RNA polymerase beta* chain
PA4270	-	-				+				+	*rpoB*	DNA-directed RNA polymerase beta chain
PA4271		-	-	+						+	*rplL*	50S ribosomal protein L7 / L12
PA4272	-			+		+				+	*rplJ*	50S ribosomal protein L10
PA4273		-		+		+		+		+	*rplA*	50S ribosomal protein L1
PA4274	-	-		+		+				+	*rplK*	50S ribosomal protein L11
PA4275	-	-		+		+	+	+		+	*nusG*	transcription antitermination protein NusG
PA4276	-	-		+		+	+	+		+	*secE*	secretion protein SecE
PA4317	-		-							+		hypothetical protein
PA4318	-	-	-							+		hypothetical protein
PA4319			-									conserved hypothetical protein
PA4405	-	-	-							+		hypothetical protein
PA4406		-						+			*lpxC*	UDP-3-O-acyl-N-acetylglucosamine deacetylase

PA4426	-	-	-			-	-			+		conserved hypothetical protein
PA4427	-	-	-				+			+	*sspB*	stringent starvation protein B
PA4428	-	-	-							+	*sspA*	stringent starvation protein A
PA4429	-	-	-							+		probable cytochrome c1 precursor
PA4430	-	-	-							+		probable cytochrome b
PA4431	-	-	-							+		probable iron-sulfur protein
PA4432	-	-	-	+		+		+	-	+	*rpsI*	30S ribosomal protein S9
PA4433	-	-	-	+		+				+	*rplM*	50S ribosomal protein L13
PA4449		-								+	*hisG*	ATP-phosphoribosyltransferase
PA4451						+		+				conserved hypothetical protein
PA4458	-	-	-							+		conserved hypothetical protein
PA4459	-	-	-							+		conserved hypothetical protein
PA4460		-	-									conserved hypothetical protein
PA4461		-	-	+				+	-	+		probable ATP-binding component of ABC transporter
PA4465										+		conserved hypothetical protein
PA4466		-								+		probable phosphoryl carrier protein
PA4479			-	+	+	+				+	*mreD*	rod shape-determining protein MreD
PA4480	-	-	-							+	*mreC*	rod shape-determining protein MreC
PA4482						+				+	*gatC*	Glu-tRNA(Gln) amidotransferase subunit C
PA4494	-	-	-							+	*roxS*	RoxS
PA4528	-	-	-							+	*pilD*	type 4 prepilin peptidase PilD
PA4530	-		-							+		conserved hypothetical protein
PA4554										+	*pilY1*	type 4 fimbrial biogenesis protein PilY1
PA4563	-	-	-	+		+	+	+	-	+	*rpsT*	30S ribosomal protein S20
PA4568	-	-		+		+		+		+	*rplU*	50S ribosomal protein L21
PA4569	-	-	-							+	*ispB*	octaprenyl-diphosphate synthase
PA4574										+		conserved hypothetical protein
PA4625	-	-	-							+	*cdrA*	cyclic diguanylate-regulated TPS partner A, CdrA
PA4640			-							+	*mqoB*	malate:quinone oxidoreductase
PA4670	-	-	-	+		+			-	+	*prs*	ribose-phosphate pyrophosphokinase
PA4671		-		+		+		+		+		probable ribosomal protein L25
PA4672	-	-	-			+				+	*pth*	peptidyl-tRNA hydrolase
PA4673	-	-		+		+				+		conserved hypothetical protein

PA4678	-	-	-								*rimI*	peptide n-acetyltransferase RimI
PA4728		-								+	*folK*	2-amino-4-hydroxy-6-hydroxymethyldihydropteridine pyrophosphokinase
PA4740						+				+	*pnp*	polyribonucleotide nucleotidyltransferase
PA4743	-	-	-							+	*rbfA*	ribosome-binding factor A
PA4744										+	*infB*	translation initiation factor IF-2
PA4745				+		+				+	*nusA*	N utilization substance protein A
PA4746	-	-	-	+		+		+	-	+		conserved hypothetical protein
PA4747	-	-				+	+	+		+	*secG*	secretion protein SecG
PA4753	-	-				+	+	+		+		conserved hypothetical protein
PA4757	-	-				+		+		+		conserved hypothetical protein
PA4765	-	-	-	+				+	-	+	*omlA*	Outer membrane lipoprotein OmlA precursor
PA4768	-	-	-								*smpB*	SmpB protein
PA4846	-	-				+		+		+	*aroQ1*	3-dehydroquinate dehydratase
PA4853	-	-	-					+	-	+	*fis*	DNA-binding protein Fis
PA4932		-		+		+		+	-	+	*rplI*	50S ribosomal protein L9
PA4934	-	-				+	+	+		+	*rpsR*	30S ribosomal protein S18
PA4935	-	-	-	+		+				+	*rpsF*	30S ribosomal protein S6
PA4962	-	-	-							+		conserved hypothetical protein
PA4965			-									hypothetical protein
PA4997						+					*msbA*	transport protein MsbA
PA5033	-	-	-							+		hypothetical protein
PA5043			-					-			*pilN*	type 4 fimbrial biogenesis protein PilN
PA5045	-	-	-							+	*ponA*	penicillin-binding protein 1A
PA5046		-										malic enzyme
PA5049	-	-				+	+	+		+	*rpmE*	50S ribosomal protein L31
PA5117	-	-		+		+				+	*typA*	regulatory protein TypA
PA5118	-	-								+	*thiI*	thiazole biosynthesis protein ThiI
PA5130		-	-							+		conserved hypothetical protein
PA5139	-	-	-							+		hypothetical protein
PA5203										+	*gshA*	glutamate--cysteine ligase
PA5239	-	-		+		+		+	-	+	*rho*	transcription termination factor Rho
PA5244						+						conserved hypothetical protein
PA5296	-									+	*rep*	ATP-dependent DNA helicase Rep

PA5300	-	-					+				*cycB*	cytochrome c5
PA5302			-								*dadX*	catabolic alanine racemase
PA5315	-	-		+	+	+	+	+	-	+	*rpmG*	50S ribosomal protein L33
PA5316	-	-			-	+	+	+	-	+	*rpmB*	50S ribosomal protein L28
PA5338		-		+		+		+	-	+	*spoT*	guanosine-3',5'-bis(diphosphate) 3'-pyrophosphohydrolase
PA5351	-						+				*rubA1*	Rubredoxin 1
PA5366	-	-	-							+	*pstB*	ATP-binding component of ABC phosphate transporter
PA5367	-	-	-							+	*pstA*	membrane protein component of ABC phosphate transporter
PA5369	-									+	*pstS*	phosphate ABC transporter, periplasmic phosphate-binding protein, PstS
PA5435		-	-							+		probable transcarboxylase subunit
PA5462										+		hypothetical protein
PA5479	-	-	-	+						+	*gltP*	proton-glutamate symporter
PA5490	-	-	-							+	*cc4*	cytochrome c4 precursor
PA5491	-	-			-			+	-	+		probable cytochrome
PA5492	-											conserved hypothetical protein
PA5505	-	-	-							+		probable TonB-dependent receptor
PA5554			-								*atpD*	ATP synthase beta chain
PA5555	-		-					-		+	*atpG*	ATP synthase gamma chain
PA5556			-								*atpA*	ATP synthase alpha chain
PA5557			-							+	*atpH*	ATP synthase delta chain
PA5560	-	-	-	+			-			+	*atpB*	ATP synthase A chain
PA5561		-			-			+			*atpI*	ATP synthase protein I
PA5563		-			-			+	-		*soj*	chromosome partitioning protein Soj
PA5568	-	-	-	+		+				+		conserved hypothetical protein
PA5569		-		+				+	-	+	*rnpA*	ribonuclease P protein component
PA5570		-								+	*rpmH*	50S ribosomal protein L34

Planktonic growth specific cluster

	bw /bf	tu/ bf	pl/ bf	bw/ lb	tu/ lb	pl/ lb	pl/ bw	pl/ tu	tu/ bw	bf/ lb		
PA0038	+					-	-			-		hypothetical protein
PA0039					-			+	-			hypothetical protein

PA0052			+	-	-		+	+		-		hypothetical protein
PA0105			+	-	-	-	+	+		-	*coxB*	cytochrome c oxidase, subunit II
PA0106				-	-	-	+	+		-	*coxA*	cytochrome c oxidase, subunit I
PA0107			+	-	-	-	+	+		-		conserved hypothetical protein
PA0108	-			-	-	-	+	+		-	*coIII*	cytochrome c oxidase, subunit III
PA0109			+	-			+	+		-		hypothetical protein
PA0110				-	-	-	+	+		-		hypothetical protein
PA0111				-	-	-				-		hypothetical protein
PA0112				-	-	-				-		hypothetical protein
PA0113				-	-	-				-		probable cytochrome c oxidase assembly factor
PA0122	-	-		-	-	-	+	+		-		conserved hypothetical protein
PA0156						-				-	*triA*	(RND) triclosan efflux membrane fusion protein, TriA
PA0157						-					*triB*	(RND) triclosan efflux membrane fusion protein, TriB
PA0173				-		-				-		probable methylesterase
PA0175				-	-	-				-	*cheR2*	probable chemotaxis protein methyltransferase
PA0176				-	-	-				-	*aer2*	aerotaxis transducer Aer2
PA0177				-	-	-				-		probable purine-binding chemotaxis protein
PA0178				-	-	-				-		probable two-component sensor
PA0179		-	+	-	-	-	+	+		-		probable two-component response regulator
PA0180				-	-	-				-	*cttP*	chemotactic transducer for trichloroethylene [positive chemotaxis], CttP
PA0200		+	+				+		+	-		hypothetical protein
PA0249				-	-	-				-		probable acetyltransferase
PA0250			+	-						-		conserved hypothetical protein
PA0256				-	-	-				-		hypothetical protein
PA0312		+	+	-	-		+			-		conserved hypothetical protein
PA0329				-	-	-				-		conserved hypothetical protein
PA0332		+	+	-						-		hypothetical protein
PA0365				-	-	-				-		hypothetical protein
PA0366						-						probable aldehyde dehydrogenase
PA0384				-	-							hypothetical protein
PA0387										-		conserved hypothetical protein
PA0388		+						-	+			hypothetical protein
PA0432		-	-			-					*sahH*	S-adenosyl-L-homocysteine hydrolase

PA0452			+				+					probable stomatin-like protein
PA0459						-		-				probable ClpA/B protease ATP binding subunit
PA0484			+	-	-			+		-		conserved hypothetical protein
PA0520		+	+	-					+	-	*nirQ*	regulatory protein NirQ
PA0540						-						hypothetical protein
PA0586				-	-	-				-		conserved hypothetical protein
PA0588	+					-				-		conserved hypothetical protein
PA0612				-		-		-		-	*ptrB*	repressor, PtrB
PA0614		+				-		-		-		hypothetical protein
PA0616						-						hypothetical protein
PA0619						-						probable bacteriophage protein
PA0624				-	-	-				-		hypothetical protein
PA0631						-	-					hypothetical protein
PA0635						-						hypothetical protein
PA0636						-						hypothetical protein
PA0648						-						hypothetical protein
PA0656			+	-			+			-		probable HIT family protein
PA0745				-	-	-				-		probable enoyl-CoA hydratase/isomerase
PA0746						-				-		probable acyl-CoA dehydrogenase
PA0762	+					-	-			-	*algU*	sigma factor AlgU
PA0776						-						hypothetical protein
PA0865				-	-	-		+		-	*hpd*	4-hydroxyphenylpyruvate dioxygenase
PA0866				-	-	-				-	*aroP2*	aromatic amino acid transport protein AroP2
PA0870				-	-	-				-	*phhC*	aromatic amino acid aminotransferase
PA0871				-	-	-				-	*phhB*	pterin-4-alpha-carbinolamine dehydratase
PA0872					-	-				-	*phhA*	phenylalanine-4-hydroxylase
PA0887	+									-	*acsA*	acetyl-coenzyme A synthetase
PA0921				-	-	-				-		hypothetical protein
PA0959				-	-	-				-		hypothetical protein
PA0960				-	-			+		-		hypothetical protein
**PA1002**				**-**	**-**		**+**				***phnB***	**anthranilate synthase component II**
PA1027						-				-		probable aldehyde dehydrogenase
PA1041			+	-	-	-	+			-		probable outer membrane protein precursor

PA1042				-								conserved hypothetical protein
PA1048				-	-	-				-		probable outer membrane protein precursor
PA1053										-		conserved hypothetical protein
PA1084								+			*flgI*	flagellar P-ring protein precursor FlgI
PA1121				-	-	-				-	*yfiR*	YfiR
PA1172				-							*napC*	cytochrome c-type protein NapC
PA1173				-			+				*napB*	cytochrome c-type protein NapB precursor
PA1174				-	-	-	+			-	*napA*	periplasmic nitrate reductase protein NapA
PA1175	-			-	-	-	+			-	*napD*	NapD protein of periplasmic nitrate reductase
PA1176			+	-	-	-	+	+		-	*napF*	ferredoxin protein NapF
PA1177		+	+	-	-		+	+	+	-	*napE*	periplasmic nitrate reductase protein NapE
PA1178				-	-	-		-	+	-	*oprH*	PhoP/Q and low Mg2+ inducible outer membrane protein H1 precursor
PA1179				-	-	-				-	*phoP*	two-component response regulator PhoP
PA1180				-	-	-				-	*phoQ*	two-component sensor PhoQ
PA1202				-		-				-		probable hydrolase
PA1246						-					*aprD*	alkaline protease secretion protein AprD
PA1247						-					*aprE*	alkaline protease secretion protein AprE
PA1249						-					*aprA*	alkaline metalloproteinase precursor
PA1283				-		-				-		probable transcriptional regulator
PA1287			+									probable glutathione peroxidase
PA1289				-	-	-				-		hypothetical protein
PA1301	+						-		-			probable transmembrane sensor
PA1327				-		-						probable protease
PA1342				-	-	-				-		probable binding protein component of ABC transporter
PA1348				-	-	-				-		hypothetical protein
PA1353				-						-		hypothetical protein
**PA1430**						**-**				**-**	***lasR***	**transcriptional regulator LasR**
**PA1431**		**-**		**-**	**-**		**+**	**+**			***rsaL***	**regulatory protein RsaL**
**PA1432**			**+**				**+**				***lasI***	**autoinducer synthesis protein LasI**
PA1454				-			+				*fleN*	flagellar synthesis regulator FleN
PA1455				-	-						*fliA*	sigma factor FliA
PA1456				-	-						*cheY*	two-component response regulator CheY
PA1458				-	-						*cheA*	probable two-component sensor

PA1462				-	-		+					probable plasmid partitioning protein
PA1464			+	-			+	+		-	*cheW*	probable purine-binding chemotaxis protein
PA1545			+	-	-		+	+				hypothetical protein
PA1617				-	-	-				-		probable AMP-binding enzyme
PA1641				-	-	-				-		hypothetical protein
PA1728		+	+	-	-		+	+		-		hypothetical protein
PA1729			+							-		conserved hypothetical protein
PA1733			+	-	-		+	+				conserved hypothetical protein
PA1752			+	-						-		hypothetical protein
PA1753				-	-		+	+				conserved hypothetical protein
PA1760				-	-					-		probable transcriptional regulator
PA1762						-						hypothetical protein
PA1784				-	-	-				-		hypothetical protein
PA1817			+				+					hypothetical protein
PA1819				-		-				-		probable amino acid permease
PA1860										-		hypothetical protein
PA1874			+	-	-		+			-		hypothetical protein
PA1875			+	-	-	-	+	+		-		probable outer membrane protein precursor
PA1876				-	-	-				-		probable ATP-binding/permease fusion ABC transporter
PA1887				-	-	-				-		hypothetical protein
PA1888				-	-	-				-		hypothetical protein
PA1914				-	-	-				-		conserved hypothetical protein
PA1931	+		+	-	-	-				-		probable ferredoxin
PA1944						-						hypothetical protein
PA1985	+		+		-			+	-	-	*pqqA*	pyrroloquinoline quinone biosynthesis protein A
PA2006				-	-	-				-		probable major facilitator superfamily (MFS) transporter
PA2007	+	+	+	-	-	-	-			-	*maiA*	maleylacetoacetate isomerase
PA2009						-				-	*hmgA*	homogentisate 1,2-dioxygenase
PA2020			+	-			+			-	*amrR*	probable transcriptional regulator
PA2024	+		+	-	-		+	+		-		probable ring-cleaving dioxygenase
PA2025			+							-	*gor*	glutathione reductase
PA2027				-	-	-				-		hypothetical protein
PA2166	+		+	-	-	-		+	-	-		hypothetical protein

PA2174	+		+		-			+		-		hypothetical protein
PA2237						-					*pslG*	PslG
PA2247			-	-	-	-	-			-	*bkdA1*	2-oxoisovalerate dehydrogenase (alpha subunit)
PA2248				-	-	-		-		-	*bkdA2*	2-oxoisovalerate dehydrogenase (beta subunit)
PA2249		+		-	-	-		-		-	*bkdB*	branched-chain alpha-keto acid dehydrogenase (lipoamide component)
PA2250				-	-	-				-	*lpdV*	lipoamide dehydrogenase-Val
PA2364			+	-	-		+			-		hypothetical protein
PA2365	-	-		-	-			+				conserved hypothetical protein
PA2367				-								hypothetical protein
PA2375			+	-			+	+		-		hypothetical protein
PA2381			-	-	-	-	-	-		-		hypothetical protein
PA2422				-		-				-		hypothetical protein
PA2423				-								hypothetical protein
PA2433	+		+		-	-	-		-	-		hypothetical protein
PA2434						-						hypothetical protein
PA2501	-			-	-	-	+		+	-		hypothetical protein
PA2504				-	-	-				-		hypothetical protein
PA2511			+				+	+				probable transcriptional regulator
PA2562			+	-	-	-				-		hypothetical protein
PA2564				-	-	-	+			-		hypothetical protein
PA2565			+	-	-	-	+	+		-		hypothetical protein
PA2566				-	-	-				-		conserved hypothetical protein
PA2571				-	-	-				-		probable two-component sensor
PA2573		+	+	-			+			-		probable chemotaxis transducer
PA2577						-						probable transcriptional regulator
**PA2587**						**-**					***pqsH***	**probable FAD-dependent monooxygenase**
PA2591	-	-		-	-		+	+				probable transcriptional regulator
PA2605			+	-			+			-		conserved hypothetical protein
PA2607			+									conserved hypothetical protein
PA2618				-	-			+		-		hypothetical protein
PA2620			+	-						-	*clpA*	ATP-binding protease component ClpA
PA2622	-		+	-	-		+	+			*cspD*	cold-shock protein CspD
PA2640	-	-	-	-	-	-					*nuoE*	NADH dehydrogenase I chain E

PA2643	-	-		-	-		+				*nuoH*	NADH dehydrogenase I chain H
PA2645			-			-					*nuoJ*	NADH dehydrogenase I chain J
PA2646	-		-	-		-					*nuoK*	NADH dehydrogenase I chain K
PA2747			+	-	-			+		-		hypothetical protein
PA2754	+	+				-	-	-		-		conserved hypothetical protein
PA2762				-	-	-				-		hypothetical protein
PA2771				-	-	-				-		conserved hypothetical protein
PA2779			+	-			+			-		hypothetical protein
PA2799			+				+	+		-		hypothetical protein
PA2827		+	+							-		conserved hypothetical protein
PA2841						-				-		probable enoyl-CoA hydratase/isomerase
PA2849				-	-	-				-	*ohrR*	OhrR
PA2883	+									-		hypothetical protein
PA2897			+		-		+	+		-		probable transcriptional regulator
PA2915				-						-		hypothetical protein
PA2920					-					-		probable chemotaxis transducer
PA2937			+	-	-		+	+		-		hypothetical protein
PA2939				-	-	-				-	*pepB*	probable aminopeptidase
PA3017	+	+	+	-	-		+	+		-		conserved hypothetical protein
PA3032				-	-	-				-	*snr1*	cytochrome c Snr1
PA3040	+	+	+			-				-		conserved hypothetical protein
PA3068				-		-				-	*gdhB*	NAD-dependent glutamate dehydrogenase
PA3186				-	-	-				-	*oprB*	Glucose/carbohydrate outer membrane porin OprB precursor
PA3187				-	-		+			-	*gltK*	probable ATP-binding component of ABC transporter
PA3188				-	-	-					*gltG*	probable permease of ABC sugar transporter
PA3216				-		-				-		hypothetical protein
PA3229			+									hypothetical protein
PA3234			+	-	-		+	+		-		probable sodium:solute symporter
PA3235			+	-	-		+	+	-	-		conserved hypothetical protein
PA3311						-						conserved hypothetical protein
PA3316			+	-	-					-		probable permease of ABC transporter
PA3343				-								hypothetical protein
PA3345				-	-							hypothetical protein

PA3346				-	-	-				-		probable two-component response regulator
PA3347				-	-	-				-		hypothetical protein
PA3348					-						*cheR1*	probable chemotaxis protein methyltransferase
PA3349			+	-	-		+	+		-		probable chemotaxis protein
PA3351			+				+	+			*flgM*	FlgM
PA3352			+			+	+	+				hypothetical protein
PA3369					-			+				hypothetical protein
PA3371			+					+		-		hypothetical protein
**PA3415**		**+**	**+**	**-**			**+**			**-**		**probable dihydrolipoamide acetyltransferase**
**PA3416**				**-**	**-**	**-**				**-**		**probable pyruvate dehydrogenase E1 component, beta chain**
PA3418			+	-	-		+	+		-	*ldh*	leucine dehydrogenase
PA3451			+	-	-	+	+	+		-		hypothetical protein
PA3465				-		-						conserved hypothetical protein
**PA3477**		**-**		**-**	**-**		**+**	**+**	**-**		***rhlR***	**transcriptional regulator RhlR**
PA3496	-	-	-	-	-							hypothetical protein
PA3526			+	-			+	+				probable outer membrane protein precursor
PA3568				-	-	-				-		probable acetyl-coa synthetase
PA3569	+			-	-	-	-			-	*mmsB*	3-hydroxyisobutyrate dehydrogenase
PA3570	+			-	-	-				-	*mmsA*	methylmalonate-semialdehyde dehydrogenase
PA3622			+	-	-			+		-	*rpoS*	sigma factor RpoS
PA3628							+					probable esterase
PA3629							+				*adhC*	alcohol dehydrogenase class III
PA3662				-	-	-				-		hypothetical protein
PA3674						-		-		-		hypothetical protein
PA3684	-	-	+	-	-		+	+		-		hypothetical protein
PA3688	+		+	-	-		+	+		-		hypothetical protein
PA3723				-	-	-				-		probable FMN oxidoreductase
PA3724				-	-	-		+		-	*lasB*	elastase LasB
PA3740			+							-		hypothetical protein
PA3753										-		conserved hypothetical protein
PA3784				-		-				-		hypothetical protein
PA3785				-		-		-		-		conserved hypothetical protein
PA3786				-	-							hypothetical protein

PA3792				-							*leuA*	2-isopropylmalate synthase
PA3833			+	-	-		+	+				hypothetical protein
PA3846			+	-			+	+		-		hypothetical protein
PA3847				-	-	-				-		conserved hypothetical protein
PA3848				-		-						hypothetical protein
PA3851			+		-		+	+				hypothetical protein
PA3858				-	-	-				-		probable amino acid-binding protein
PA3922				-	-	-				-		conserved hypothetical protein
PA3923				-	-							hypothetical protein
PA3945				-	-	-				-		conserved hypothetical protein
PA3957			+	-	-		+	+		-		probable short-chain dehydrogenase
PA3986		+	+	-	-		+	+		-		hypothetical protein
PA4012			+	-	-		+	+		-		hypothetical protein
PA4079				-		-				-		probable dehydrogenase
PA4112				-	-	-				-		probable sensor/response regulator hybrid
PA4129	-	-	-	-	-	-	+					hypothetical protein
PA4218				-		-				-	*ampP*	AmpP
PA4219				-	-	-				-		hypothetical protein
PA4220	+	+	+	-	-	-				-		hypothetical protein
PA4221	+			-	-	-				-	*fptA*	Fe(III)-pyochelin outer membrane receptor precursor
PA4222				-	-	-				-	*pchI*	probable ATP-binding component of ABC transporter
PA4223	+			-	-	-				-	*pchH*	probable ATP-binding component of ABC transporter
PA4224	+			-	-	-	-			-	*pchG*	pyochelin biosynthetic protein PchG
PA4225				-	-	-				-	*pchF*	pyochelin synthetase
PA4226				-	-	-				-	*pchE*	dihydroaeruginoic acid synthetase
PA4227	+				-	-	-		-	-	*pchR*	transcriptional regulator PchR
PA4228				-	-	-				-	*pchD*	pyochelin biosynthesis protein PchD
PA4229				-	-	-				-	*pchC*	pyochelin biosynthetic protein PchC
PA4230	+			-	-	-				-	*pchB*	salicylate biosynthesis protein PchB
PA4231				-	-	-				-	*pchA*	salicylate biosynthesis isochorismate synthase
PA4290						-						probable chemotaxis transducer
PA4294			+	-	-		+	+		-		hypothetical protein
PA4296			+	-	-		+	+		-	*pprB*	two-component response regulator, PprB

PA4297				-	-	-				-	*tadG*	TadG
PA4298				-	-	-				-		hypothetical protein
PA4299				-	-	-				-	*tadD*	TadD
PA4300			+	-	-	-				-	*tadC*	TadC
PA4301				-	-	-				-	*tadB*	TadB
PA4302				-	-	-				-	*tadA*	TadA ATPase
PA4303				-	-	-				-	*tadZ*	TadZ
PA4304				-	-	-				-	*rcpA*	RcpA
PA4305			+	-	-	-	+	+		-	*rcpC*	RcpC
PA4306	-		+	-	-		+	+		-	*flp*	Type IVb pilin, Flp
PA4311										-		conserved hypothetical protein
PA4315		-			-				-		*mvaT*	transcriptional regulator MvaT, P16 subunit
PA4324	-	-		-	-		+	+				hypothetical protein
PA4377				-	-	-		+		-		hypothetical protein
PA4410						-		-			*ddlB*	D-alanine--D-alanine ligase
PA4411						-		-			*murC*	UDP-N-acetylmuramate--alanine ligase
PA4412						-					*murG*	UDP-N-acetylglucosamine--N-acetylmuramyl-(pentapeptide)
PA4414						-	-				*murD*	UDP-N-acetylmuramoylalanine--D-glutamate ligase
PA4415			-			-					*mraY*	phospho-N-acetylmuramoyl-pentapeptide-transferase
PA4417						-		-			*murE*	UDP-N-acetylmuramoylalanyl-D-glutamate-2, 6-diaminopimelate ligase
PA4418						-		-			*ftsI*	penicillin-binding protein 3
PA4420						-						conserved hypothetical protein
PA4474	+					-				-		conserved hypothetical protein
PA4507				-	-	-				-		hypothetical protein
PA4572			+	-	-	-	+	+		-	*fklB*	peptidyl-prolyl cis-trans isomerase FklB
PA4573			+	-	-		+	+		-		hypothetical protein
PA4590				-	-	-	+	+		-	*pra*	protein activator
PA4607				-	-	-				-		hypothetical protein
PA4608		+	+	-	-		+	+		-		hypothetical protein
PA4611			+	-	-					-		hypothetical protein
PA4633			+	-			+					probable chemotaxis transducer
PA4641				-	-	-				-		still frameshift hypothetical protein
PA4648				-	-	-				-		hypothetical protein

PA4651				-		-				-		probable pili assembly chaperone
PA4702			+	-	-		+	+		-		hypothetical protein
PA4703				-	-	-		+		-		hypothetical protein
PA4713			+	-	-			+		-		hypothetical protein
PA4717			+	-	-			+		-		conserved hypothetical protein
PA4733				-		-				-	*acsB*	acetyl-coenzyme A synthetase
PA4735				-		-						hypothetical protein
PA4736				-		-						hypothetical protein
PA4766					-	-				-		conserved hypothetical protein
PA4767				-	-	-				-		conserved hypothetical protein
PA4778		-		-	-		+	+	-		*cueR*	CueR
PA4781			+	-	-		+	+		-		cyclic di-GMP phosphodiesterase
PA4782				-		-						hypothetical protein
PA4793			+									hypothetical protein
PA4810				-					+		*fdnI*	nitrate-inducible formate dehydrogenase, gamma subunit
PA4811				-		-				-	*fdnH*	nitrate-inducible formate dehydrogenase, beta subunit
PA4812				-						-	*fdnG*	formate dehydrogenase-O, major subunit
PA4841						-						conserved hypothetical protein
PA4874			+	-	-					-		conserved hypothetical protein
PA4876	+	+	+							-	*osmE*	osmotically inducible lipoprotein OsmE
PA4880			-			-	-	-	-			probable bacterioferritin
PA4913			+	-	-		+	+		-		probable binding protein component of ABC transporter
PA4915				-	-	-				-		probable chemotaxis transducer
PA4925				-	-	-	+	+		-		conserved hypothetical protein
PA4929				-	-	-				-		hypothetical protein
PA5039					-						*aroK*	shikimate kinase
PA5062			+				+	+				conserved hypothetical protein
PA5097						-						probable amino acid permease
PA5101	-			-	-	-						hypothetical protein
PA5148						-						conserved hypothetical protein
PA5153				-	-					-		amino acid ABC transporter periplasmic binding protein
PA5163		-			-						*rmlA*	glucose-1-phosphate thymidylyltransferase
PA5178										-		conserved hypothetical protein

PA5208				-	-	-				-		conserved hypothetical protein
PA5213				-	-	-				-	*gcvP1*	glycine cleavage system protein P1
PA5214				-							*gcvH1*	glycine cleavage system protein H1
PA5245						-	-	-		-		conserved hypothetical protein
PA5253			+				+			-	*algP*	alginate regulatory protein AlgP
PA5255			+	-	-		+	+		-	*algQ*	Alginate regulatory protein AlgQ
PA5261	+	+	+	-						-	*algR*	alginate biosynthesis regulatory protein AlgR
PA5301		-		-	-			+				probable transcriptional regulator
PA5348		-	+	-	-	-	+	+		-		probable DNA-binding protein
PA5350				-	-		+				*rubA2*	Rubredoxin 2
PA5359			+	-		-				-		hypothetical protein
PA5424			+	-	-					-		conserved hypothetical protein
PA5482	+		+		-				-	-		hypothetical protein
PA5527			+	-	-		+	+		-		hypothetical protein
PA5543				-	-	-						hypothetical protein
PA5545										-		conserved hypothetical protein
PA5546	+	+		-		-				-		conserved hypothetical protein
