# Supplementary figures and images for: In-Vivo Expression Profiling of Pseudomonas aeruginosa Infections Reveals Niche-Specific and Strain-Independent Transcriptional Programs
Source: PLoS One. 2011 Sep 12;6(9):e24235. doi: 10.1371/journal.pone.0024235 (PMC3171414; doi:10.1371/journal.pone.0024235)

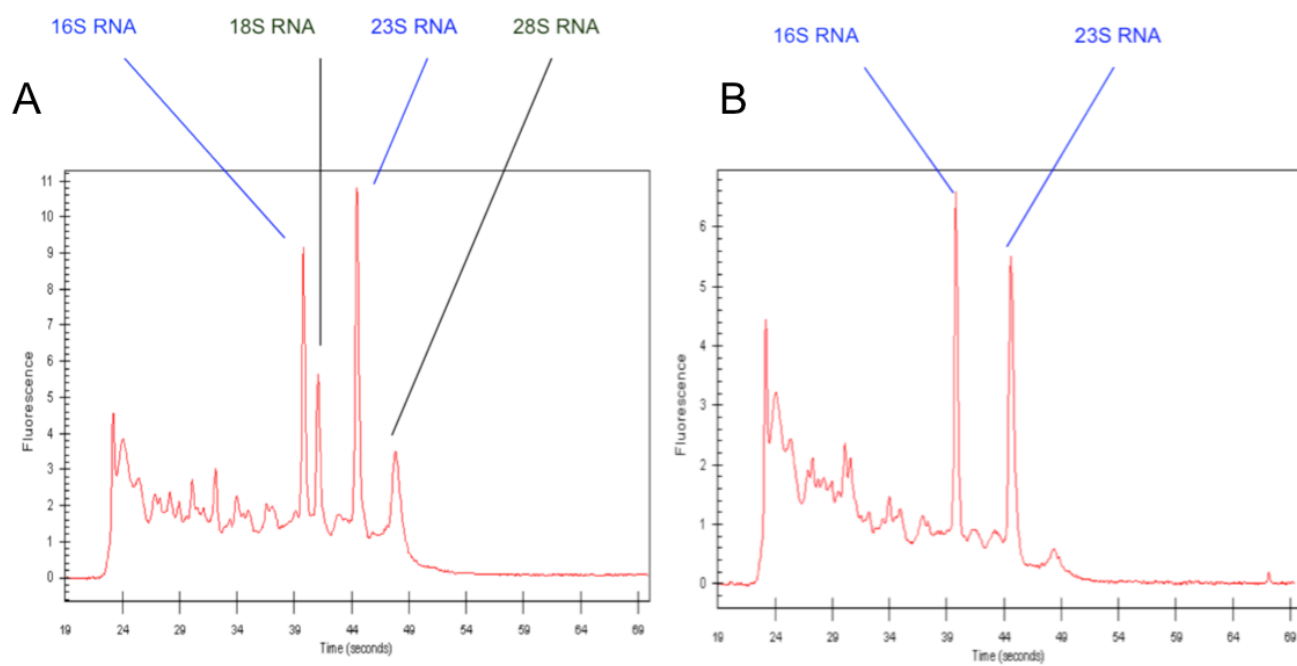

Figure S1

Supplement: Figure S1 — Agilent Bioanalyzer results of bacterial RNA enrichment from sample PBCLOp10. A) Sample before enrichment, peaks from bacterial ribosomal RNA (16S and 23S) are seen together with eukaryotic ribosomal RNA (18S and 28S). B) Sample after enrichment, only bacterial ribosomal signals are detected. (PDF) [file pone.0024235.s010.pdf]

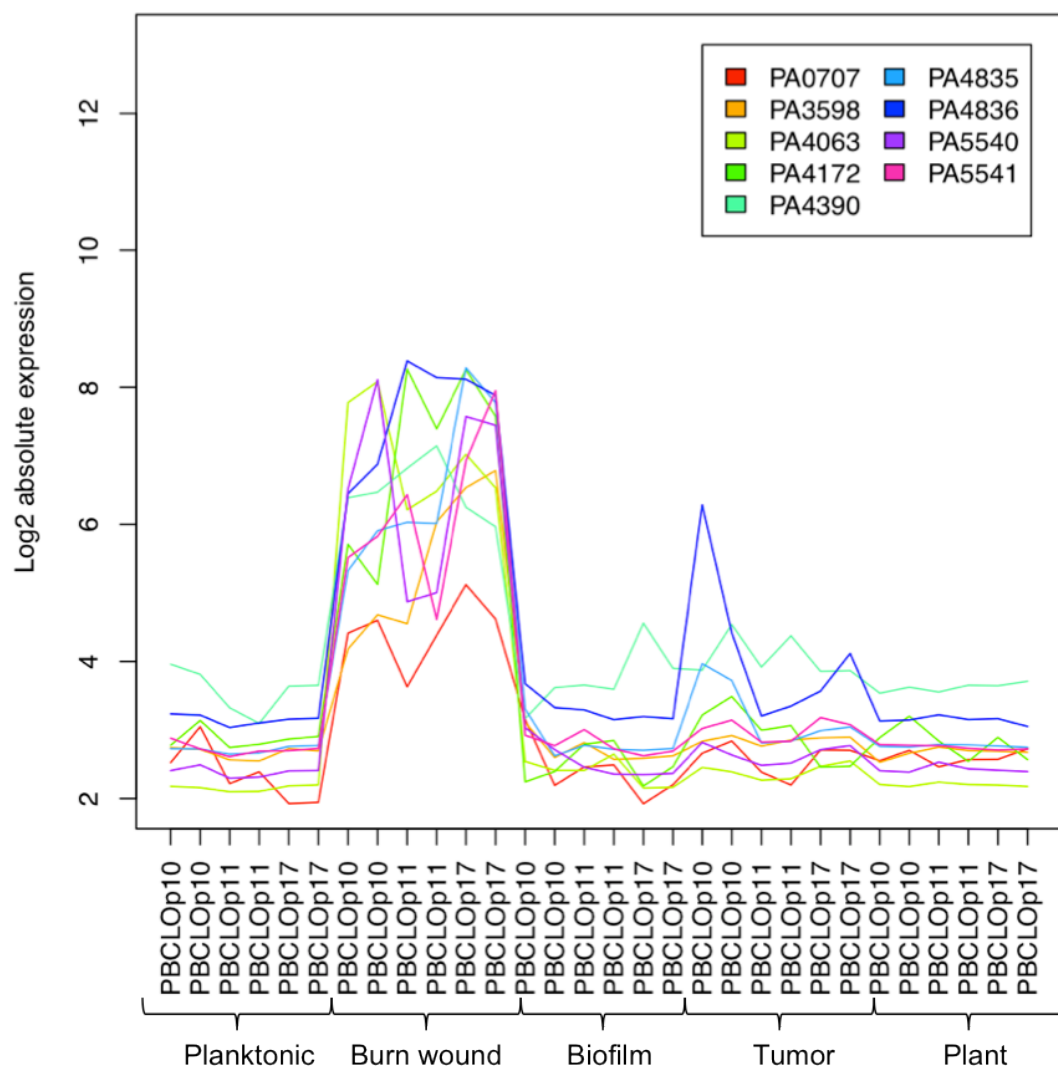

Figure S2

Supplement: Figure S2 — The genes that were expressed exclusively under in vivo burn wound infections. Gene PA0707 (toxR) encoding the regulator activating expression of toxA, which codes for Exotoxin A (Hamood and Iglewski 1990). Genes PA4835-6 and PA4836 mentioned above also belong to this cluster. Other burn wound specific genes were: PA3598 encoding a conserved hypothetical protein predicted to be N-carbamoylputrescine amidase, which catalyzes the hydrolysis of N-carbamoylputrescine to putrescine. It represents the final step of the arginine decarboxylase pathway of putrescine biosyntheseis operating in some plant and bacterial species; PA4172 encoding exodeoxyribonuclease III involved in DNA repair due to oxidative/nitrosative stress; gene pyrQ (pyrC2) (PA5541) encoding dihydroorotase involved in pyrimidine metabolism; PA5540 encoding carbonic anhydrase related protein. Lastly, PA4390 passed through the stringent filtering process encoding a hypothetical protein. (PDF) [file pone.0024235.s011.pdf]

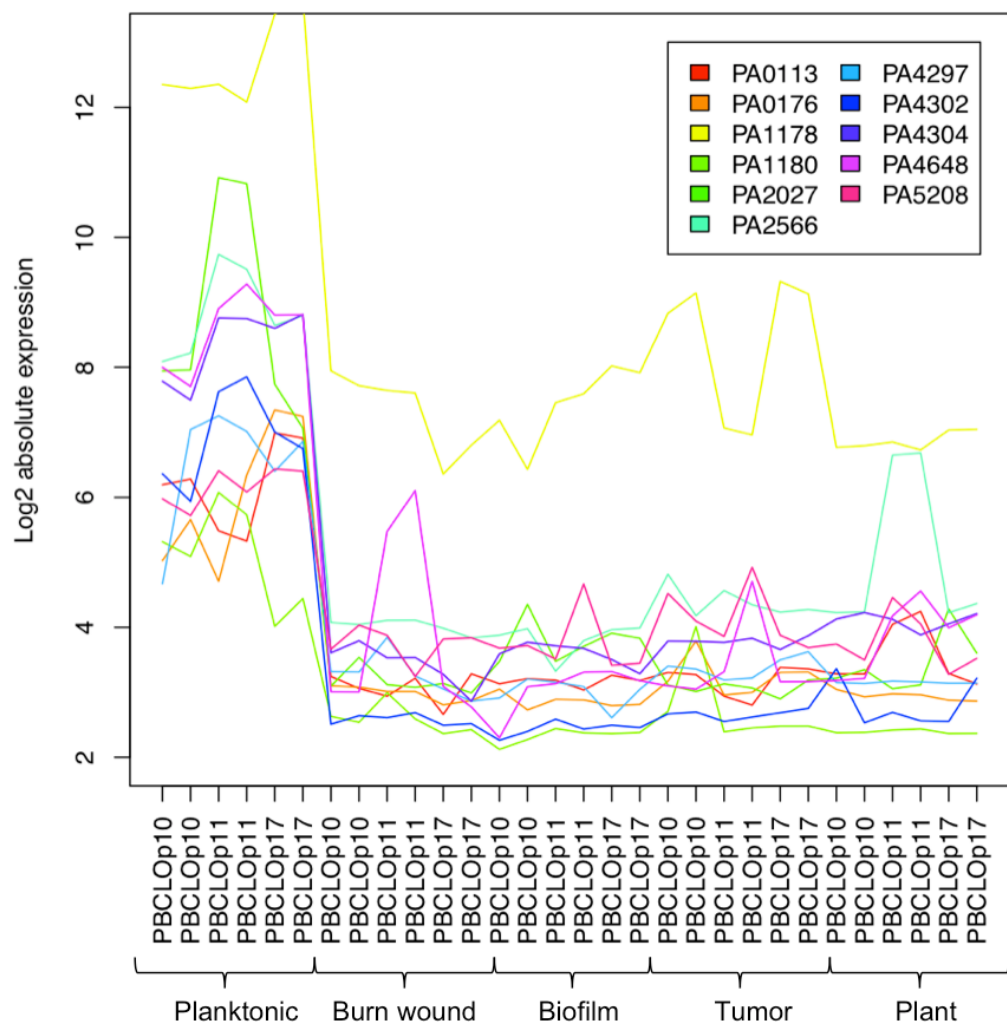

Figure S3

Supplement: Figure S3 — The genes that were expressed exclusively under planktonic in vitro conditions. PA1178 – encoding OprH protein and PA1180 - PhoQ – two component system responsible for sensing Mg limitation. PA0113 - probable cytochrome c oxidase assembly factor. PA2566 – pyridine nucleotide-disulfide family oxidoreductase. PA4297 – the Flp assembly machinery. PA4302 and PA4304 (tadA, rcpA) are part of pathway encoding for Type IV pillus assembly, where the gene PA4297 is also required. PA4648 – unknown with export signal sequence. PA5208 – conserve hypothetical phosphate transport regulator (distant homolog of PhoU). (PDF) [file pone.0024235.s012.pdf]

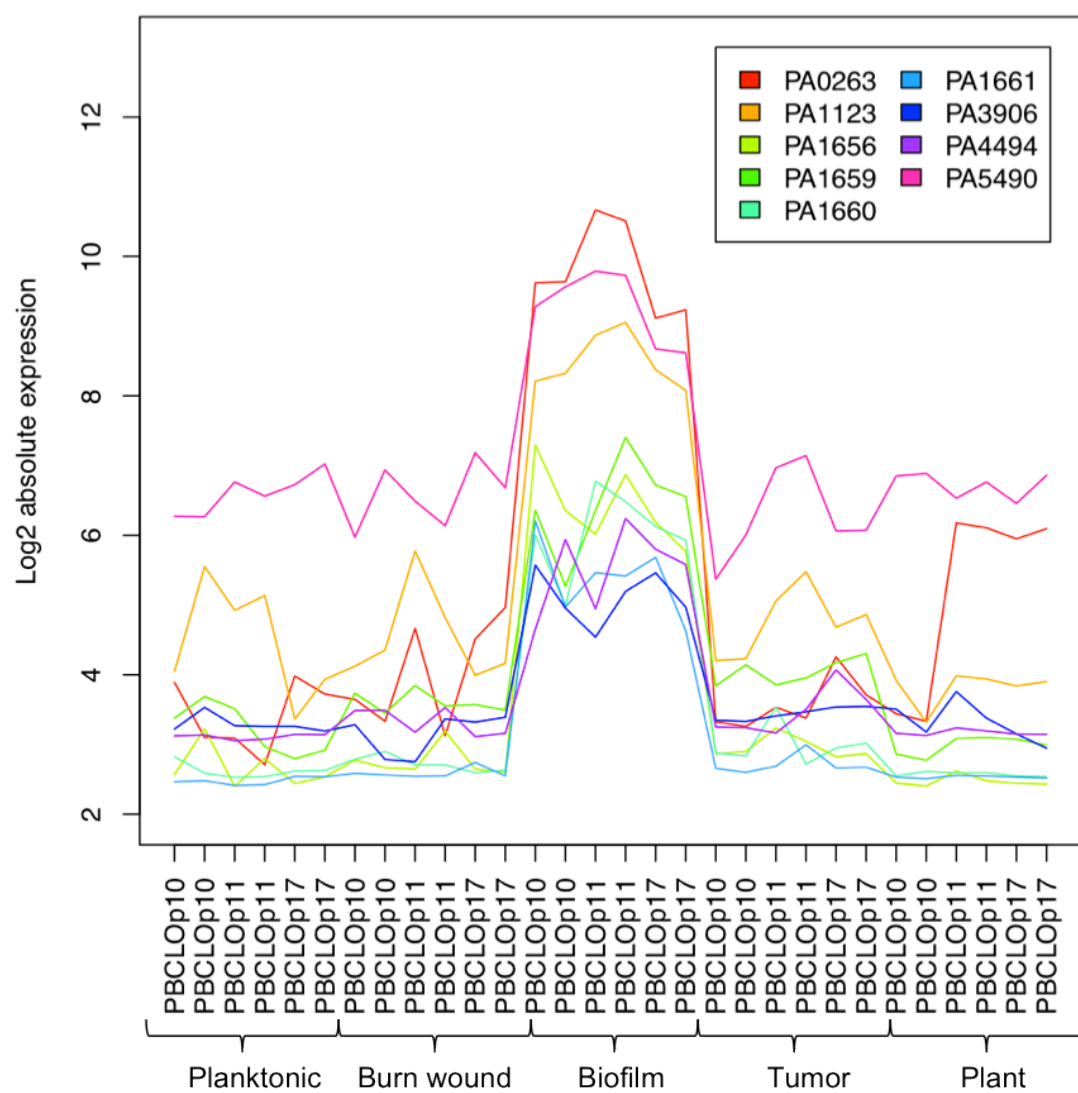

Figure S4

Supplement: Figure S4 — The genes that were expressed exclusively under the biofilm in vitro conditions. PA0263 – hcpC - encoding hemolisin co-regulated protein. PA1656, 59, 60, 61 – encoding putative type VI secretion system. PA4494 – putative two component system. PA5490 - cytochrome c4 precursor. PA1123 and PA3906 hypothetical unknown genes. (PDF) [file pone.0024235.s013.pdf]

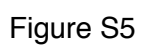

Figure S5

Supplement: Figure S5 — The genes that were expressed exclusively under the in vivo tumor infection. PA0415 – chpC - encoding putative chemotaxis protein. PA0518 – nirM, cytochrome c-551 precursor. PA1195 - N-Dimethylarginine dimethylaminohydrolase (Amino acid transport and metabolism). (PDF) [file pone.0024235.s014.pdf]

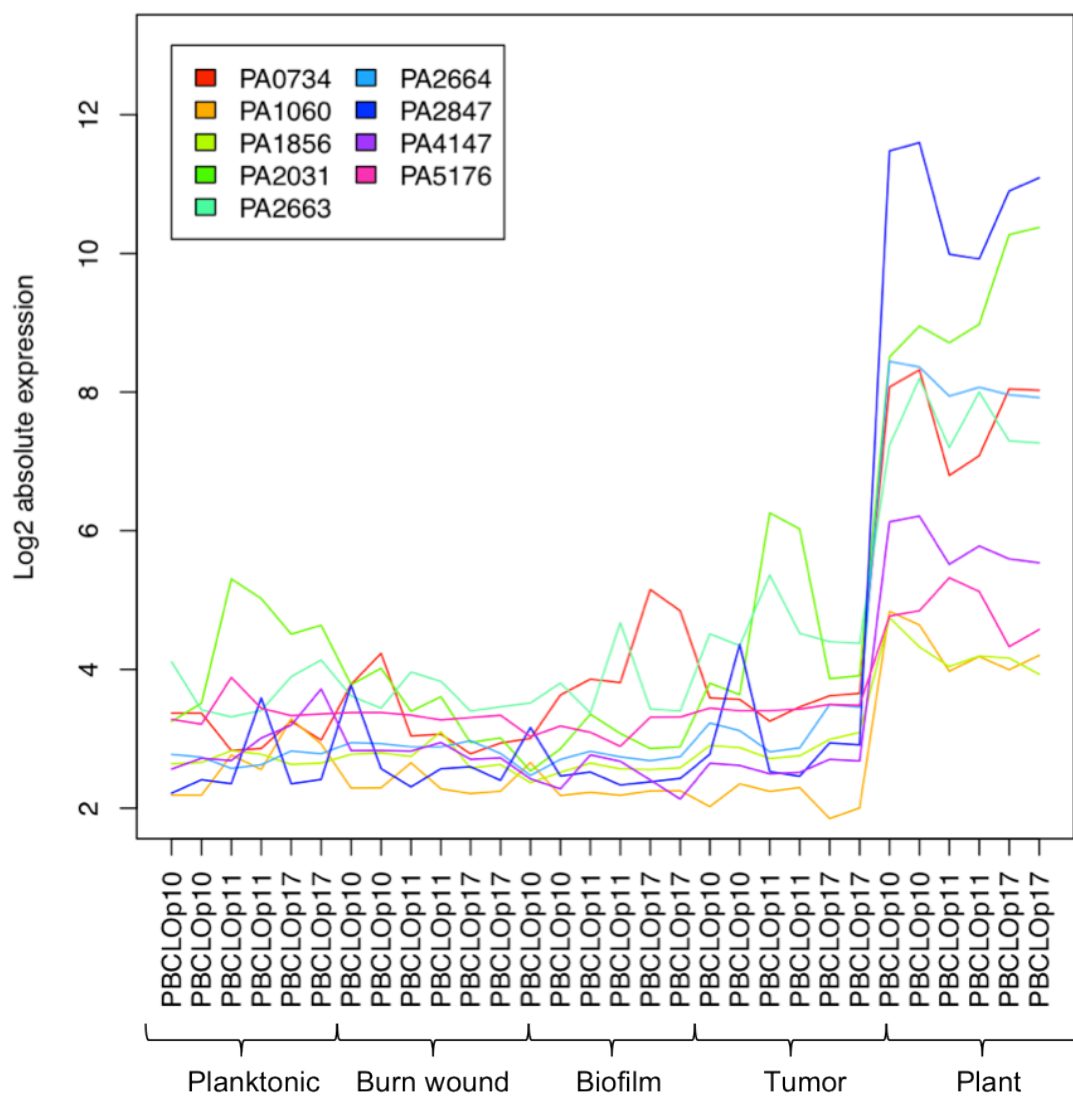

Figure S6

Supplement: Figure S6 — The genes that were expressed exclusively under the in vivo plant infections. PA0734 – encoding hypothetical unknown protein. PA1060 - predicted permease, DMT superfamily. PA1856 – encoding probable cytochrome oxidase subunit. PA2031 – hypothetical unknown. PA2663 encoding membrane protein of unknown function. PA2664 – fhp, flavohemoprotein, aerobic nitric oxide detoxification. PA2847 – predicted permease. PA4147 – acoR, transcriptional activator of acetoin/glycerol. PA5176 – conserved hypothetical gene. (PDF) [file pone.0024235.s015.pdf]

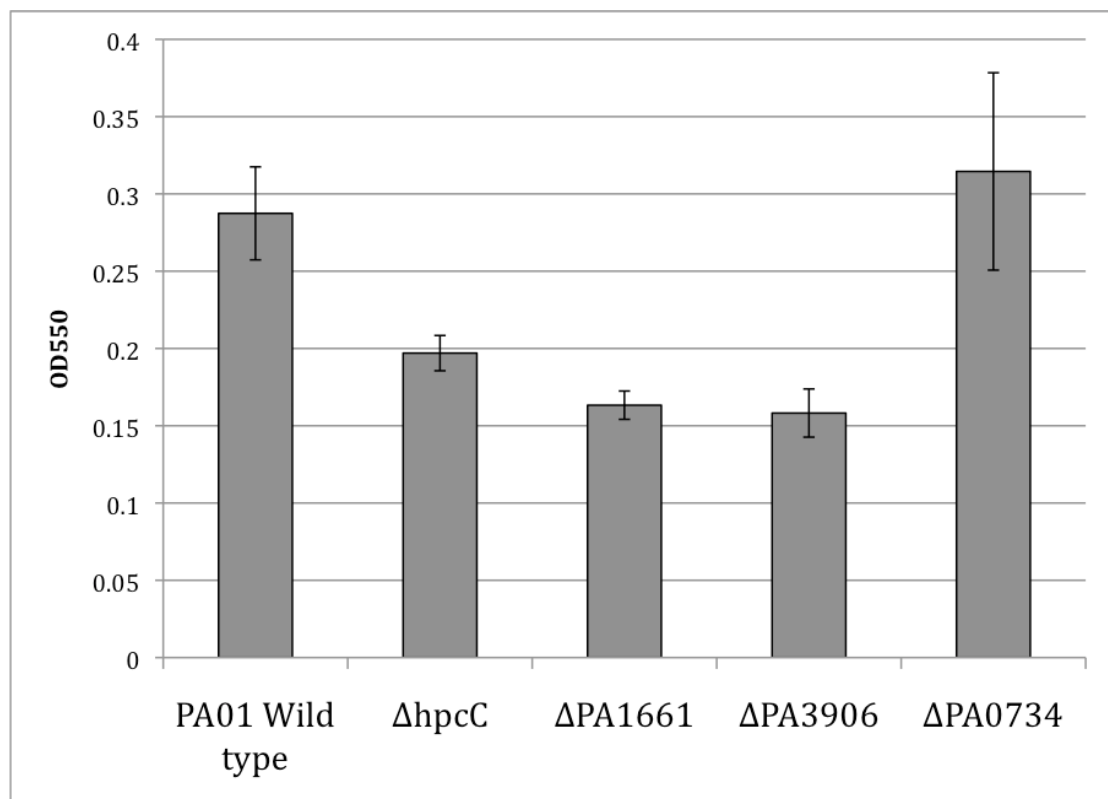

Figure S7

Supplement: Figure S7 — P. aeruginosa PAO1 wild type and mutant strains biofilm formation evaluation performed by crystal violet assay. An error bars were calculated from eight replicates and two independent experiments. (PDF) [file pone.0024235.s016.pdf]

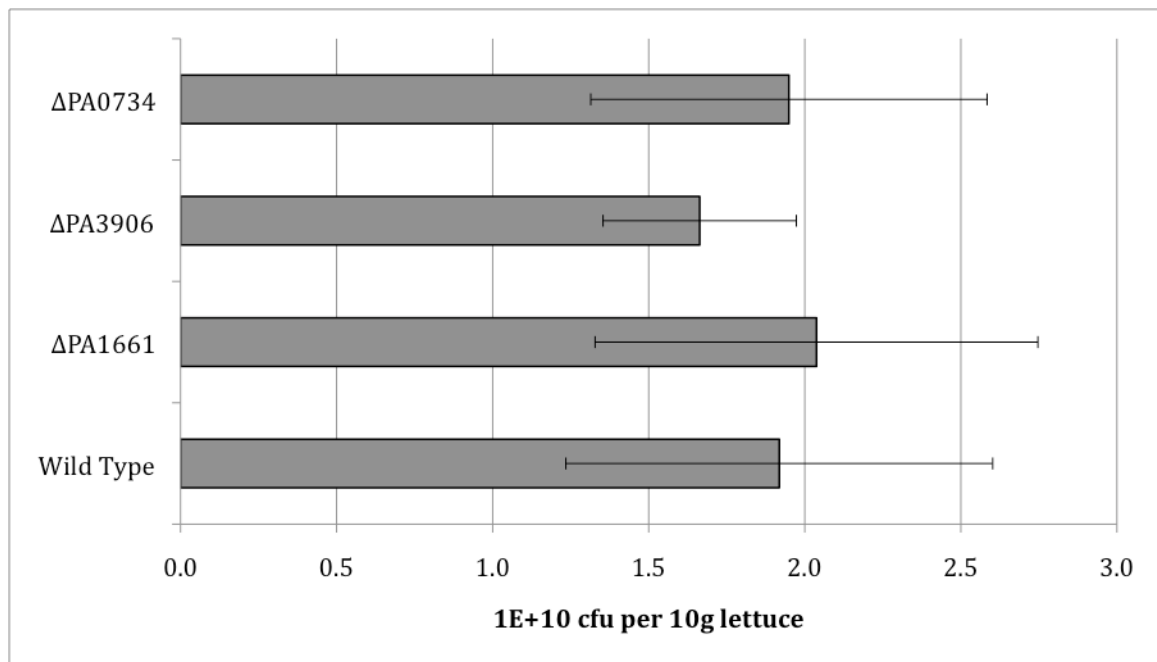

Figure S8

Supplement: Figure S8 — Virulence of the P. aeruginosa PAO1 wild type and mutant strain in the lettuce leaf assay. The number of bacterial cells (as colony forming units, cfu) present in 10 g of lettuce midrib 3 days post injection is shown. Error bars were calculated from three independent experiments. (PDF) [file pone.0024235.s017.pdf]
